# A Supportive Role of Mesenchymal Stem Cells on Insulin-Producing Langerhans Islets with a Specific Emphasis on The Secretome

**DOI:** 10.3390/biomedicines11092558

**Published:** 2023-09-18

**Authors:** Ronit Vogt Sionov, Ronit Ahdut-HaCohen

**Affiliations:** 1 The Institute of Biomedical and Oral Research (IBOR), Faculty of Dental Medicine, The Hebrew University of Jerusalem, Jerusalem 9112102, Israel; 2 Department of Medical Neurobiology, Institute of Medical Research, Hadassah Medical School, The Hebrew University of Jerusalem, Jerusalem 9112102, Israel; ronit.ahdut@mail.huji.ac.il; 3 Department of Science, The David Yellin Academic College of Education, Jerusalem 9103501, Israel

**Keywords:** β-cells, growth factors, insulin, Langerhans’ islets, mesenchymal stem cells

## Abstract

Type 1 Diabetes (T1D) is a chronic autoimmune disease characterized by a gradual destruction of insulin-producing β-cells in the endocrine pancreas due to innate and specific immune responses, leading to impaired glucose homeostasis. T1D patients usually require regular insulin injections after meals to maintain normal serum glucose levels. In severe cases, pancreas or Langerhans islet transplantation can assist in reaching a sufficient β-mass to normalize glucose homeostasis. The latter procedure is limited because of low donor availability, high islet loss, and immune rejection. There is still a need to develop new technologies to improve islet survival and implantation and to keep the islets functional. Mesenchymal stem cells (MSCs) are multipotent non-hematopoietic progenitor cells with high plasticity that can support human pancreatic islet function both in vitro and in vivo and islet co-transplantation with MSCs is more effective than islet transplantation alone in attenuating diabetes progression. The beneficial effect of MSCs on islet function is due to a combined effect on angiogenesis, suppression of immune responses, and secretion of growth factors essential for islet survival and function. In this review, various aspects of MSCs related to islet function and diabetes are described.

## 1. Introduction

Type 1 diabetes (T1D) or juvenile diabetes is a chronic autoimmune disease in which insulin-producing β-cells in the endocrine pancreas are gradually destroyed by immune cells, eventually leading to insufficient insulin production and uncontrollably fluctuating serum glucose levels [1,2,3,4]. Type 2 diabetes (T2D) is a metabolic disease where peripheral tissues, such as muscle and liver, have developed resistance to insulin signaling, reducing the ability of the tissues to take up glucose, eventually leading to hyperglycemia [4,5,6]. T2D can also develop when the β-cell mass decreases as a result of the cytotoxic effects of chronic hyperglycemia, chronic low-grade inflammation, excessive reactive oxygen species production, endoplasmic reticulum (ER) stress, and islet amyloid polypeptide deposition [7,8,9,10]. Glucose tolerance is often reduced in the elderly due to a combined effect of peripheral insulin resistance and impaired insulin secretion [11,12,13,14].

T1D is characterized by chronic inflammation and immune cell infiltration of the islets in a process termed insulitis [15,16,17,18,19,20,21]. Both hypoglycemia and hyperglycemia lead to health complications [22,23]. Hyperglycemia leads to macrovascular and microvascular complications, such as retinopathy, nephropathy, neuropathy, and cardiovascular diseases [24,25]. Chronic hyperglycemia also leads to alterations in islet cytoarchitecture with α-cell hyperplasia, β-cell transdifferentiation into glucagon-secreting cells, and deregulated hormone secretion [26]. T2D patients exhibit elevated glucagon secretion [26,27], and T1D patients secrete more glucagon during mixed-meal stimulation [28,29]. The elevated glucagon levels in T2D individuals may be due to α-cell resistance to insulin and somatostatin, whose function is to reduce glucagon secretion [26,30,31]. Thus, a vicious cycle is generated.

The destruction of β-cells is mediated by cytotoxic CD8^+^ T lymphocytes that mistakenly recognize β-cells as foreign bodies, but other immune cells also contribute to this process, including B lymphocytes that produce autoantibodies, and macrophages, dendritic cells, and neutrophils, which produce cytokines, chemokines, reactive oxygen and nitrogen species, and other bioactive molecules, and act as antigen-presenting cells [1,3,15,16,17,18,19,21,32,33,34,35,36,37]. Besides modulating immune responses, the combined production of inflammatory cytokines such as TNFα, IL-1β, and IFNγ, is detrimental to β-cells [35,38,39,40,41,42,43]. These cytokines cause mitochondrial dysfunction and endoplasmic reticulum stress, induce the expression of pro-apoptotic molecules, and activate apoptotic pathways in β-cells [3,43,44,45,46,47]. 

To maintain adequate blood glucose levels, T1D patients need exogenous insulin administration in the form of subcutaneous injections. Alternative treatments include islet cell transplantation or whole pancreas transplantation, which requires subsequent immunosuppressive therapy [48,49,50,51,52,53]. Up to 80% of the transplanted islets are lost before becoming integrated into tissue due to acute inflammatory responses and release of the pro-inflammatory cytokines IL-1β, TNFα, and IFNγ [9,48,54,55,56,57]. The success of islet transplantation is also challenged by allo-immune graft rejection and recurrence of autoimmunity [58,59,60]. Moreover, the supply of donor tissues is limited.

Mesenchymal stem cells (MSCs) are multipotent non-hematopoietic progenitor cells found in various tissues, including the bone marrow, adipose tissue, liver, and umbilical cord blood. They can differentiate into various cell types, including osteocytes, adipocytes, chondrocytes, endothelial cells, and myocytes [61]. Their low immunogenicity, together with immunosuppressive properties, has made MSCs a promising therapeutic tool for various autoimmune diseases, including T1D [48]. Several studies have shown that MSCs, by virtue of their immunomodulatory and pro-angiogenetic effects, can attenuate immune responses and enhance islet engraftment following transplantation [62,63]. This review focuses on the beneficial effects of MSCs on β-cell function, with a specific emphasis on the secretome. A brief introduction to insulin-producing β-cells and the hazardous effects of pro-inflammatory cytokines on β-cells proceeds the discussion on the different aspects of MSCs involved in preserving β-cell function.

## 2. Insulin-Producing β-Cells

Insulin-producing β-cells are the major cell type of Langerhans islets (around 60% of the islet cells), intermixed with other cell types, including glucagon-producing α-cells (around 30% of the islet cells), somatostatin-producing δ-cells (less than 10% of islet cells), pancreatic polypeptide-producing γ or PP-cells (less than 5% of islet cells), ghrelin-producing ε-cells, supportive pericytes and contractile smooth muscle cells [64,65,66,67]. The endocrine pancreas is not a single organ, but it is rather composed of millions of islets scattered throughout the exocrine pancreas [64,65], although some clusters of small islets have been found in the human pancreas [65]. These smaller islets consist of more β-cells and have a higher insulin content than the large islets [68,69]. Concerted regulation of insulin secretion and glucagon secretion is important for maintaining glucose homeostasis [64]. 

### 2.1. Vascularization of the Islets

The islets are highly infiltrated by blood vessels, enabling immediate sensation of changes in serum glucose levels as well as direct and prompt secretion of insulin and glucagon into the bloodstream as needed. The blood vessels also deliver oxygen required for β-cell function and survival [70]. Approximately 10% of the blood flow in the pancreas is delivered to the pancreatic islets despite comprising only 1–2% of the tissue mass. The smaller islets are frequently found clustered around the microcapillary beds of the endocrine pancreas [65,70], compensating for their lack of intra-islet capillaries [66,71]. In contrast, the larger islets are supplied by up to three arterioles [66,70,71]. The intra-islet endothelial cells, which are attracted by β-cells through the secretion of vascular endothelial growth factor (VEGF)-A [72], enhance insulin secretion and stimulate β-cell proliferation [73], among others, through the production of basement membrane proteins, such as laminins [74,75]. The production of the angiogenetic factor angiopoietin-1 (ANG1) by β-cells stabilizes the blood vessels in the islets, which indirectly affects insulin secretion and glucose homeostasis [76]. Gan et al. [77] emphasized the importance of extracellular matrix proteins in β-cell function. This research group observed enriched insulin granule fusion in culture β-cells that have adhered to the extracellular matrix, which was dependent on β1 integrin receptor activation [77]. The importance of the basement membrane in supporting β-cell insulin secretion is further exemplified by the improved islet function and β-cell function observed when seeded on various extracellular matrix components or on tissue decellularized extracellular matrices, especially of the lung [55,78,79,80,81]. The incorporation of laminin and collagen IV into islet alginated microcapsules protected the islets from cytokine-mediated cell death [82].

### 2.2. Innervation of the Islets

Moreover, the islets are highly innervated, and their function is affected by signals delivered by neurotransmitters of both the sympathetic and parasympathetic nervous systems [83,84,85,86]. The brain perceives glucose levels both directly and indirectly, transducing signals to regulate islet function [85,87,88,89]. Particular attention has been paid to the inhibitory neurotransmitter γ-aminobutyric acid (GABA) [90,91]. Long-term exposure of α-cells to GABA resulted in reduced glucagon secretion and transdifferentiation into β-like cells [92,93]. Treatment of human islets with GABA resulted in decreased α-cell content with a concomitant increased β-cell content [92].

### 2.3. Cell Communication within the Islets

There is also continuous communication between the cells within the islets, with mutual modulation of the activity of neighboring cells [84,94,95,96,97]. This communication is mediated by paracrine factors and juxtacrine mechanisms involving conduction of electric waves through gap junctions formed by connexin-36 (Cx36) [71,94,96,98,99,100,101]. Insulin signals α-cells to reduce glucagon secretion via the insulin receptor and the GABA-GABA-A receptor system [31,102,103,104]. Vice versa, glucagon has an impact on β-cell function [26]. Prevention of glucagon signaling using a neutralizing antibody to the glucagon receptor promoted β-cell survival and increased insulin secretion [105]. β- and δ-cells are electrically coupled through gap junctions [95]. Glucose-mediated depolarization of β-cells leads to coupled δ-cell depolarization with consequent secretion of somatostatin from δ-cells and somatostatin-mediated inhibition of α-cell glucagon secretion [95]. Glucagon secretion from α-cells is also regulated by β-cells in a juxtacrine manner, where ephrin ligands on the β-cells interact with EphA receptors on α-cells, resulting in reduced glucagon secretion [106,107]. The EphA-ephrin A system is also involved in β-cell to β-cell communication and regulates insulin secretion [108,109]. In this case, EphA receptor phosphorylation provides forward inward signals that inhibit insulin secretion, while glucose stimulation leads to dephosphorylation of EphA, allowing ephrin A reverse signaling that enhances insulin secretion [108,109]. The β-cell to β-cell communication ensures low insulin secretion during starvation while enhancing glucose-stimulated insulin secretion [108].

## 3. Destruction of β-Cells by Cytokines

The pro-inflammatory cytokines IFNγ, IL-1β, and TNFα secreted by various immune cells involved in islet inflammation (insulitis), induce apoptosis of β-cells, and together with other immune cell reactions, including FasL, perforin, granzyme, and the nitric oxide radical (NO·), contribute to the inflammatory-induced reduction in β cell mass [3,40,44,45,46,110,111,112,113,114,115,116,117,118]. The pro-inflammatory cytokines also induce chemokine production by the islet β-cells, which further exaggerates inflammation by attracting additional immune cells to the already inflamed site [115,119,120]. Each cytokine can act on its own, but the combination of two or three of them leads to large alterations in gene expression that ultimately impair β-cell survival and function [40,46,112,113,114,121,122,123,124,125]. Non-obese diabetic (NOD) mice lacking TNFα receptor 1 (TNFR1 or TNFRp55) or IL-1 receptor showed delayed onset of diabetes [117,126]. Blocking TNFα with Etanercept, a human tumor necrosis factor receptor (TNFR) p75 Fc fusion protein, resulted in lower A1C levels and increased insulin production in children with early-onset T1D, suggesting that this treatment may preserve β-cell function [127]. Quattrin et al. [128] used a neutralizing antibody to TNFα (Golimumab) in a clinical trial in children and young adults with early-onset T1D, which resulted in improved endogenous insulin secretion, but all patients still required exogenous insulin.

### 3.1. Signal Transduction Pathways Induced in Islets and β-Cells by Pro-Inflammatory Cytokines

IL-1β, IFNγ, and TNFα induce different as well as parallel signal transduction pathways, which act in concert to induce β-cell apoptosis. While IL-1β and TNFα activate the NFκB signaling pathway, IFNγ acts primarily through Janus kinase (JAK)-mediated activation of the transcription factor STAT1 [125,129,130,131,132,133,134]. NFκB-mediated signaling is pro-apoptotic in β-cells, whereas it induces anti-apoptotic pathways in most other cell types [41,132,133,135,136]. Inhibition of NFκB signaling protected β-cells from cytokine-induced apoptosis and increased islet survival after transplantation [135,136,137,138]. The cytokine-induced activation of NFκB reduced *PDX1*, *NKX2-2*, *SLC2A2*, *MAFA*, *GLUT2*, and insulin (*INS1*) gene expression while increasing *c-MYC* expression in β-cells [139,140]. Thus, these cytokines can contribute to the dedifferentiation of β-cells [139]. TNFα and IL-1β also activate the p38 and JNK mitogen-activated protein kinases (MAPKs) in β-cells [129]. Excessive p38 and JNK activation by IL-1β has been associated with β-cell apoptosis [141,142]. 

### 3.2. Gene Expression Altered in Islets and β-Cells by Pro-Inflammatory Cytokines

Several transcriptome and microarray analyses have been performed to pinpoint the genes affected in islets or β-cells in response to the pro-inflammatory cytokines [39,45,114,140,143,144,145,146,147,148]. Cytokine-induced genes relevant to insulitis and β-cell damage included inducible nitric oxide synthase (iNOS) [39,114,140,143,149,150,151,152,153], caspase 1 [39,154], cyclooxygenase (COX)-2 [149,150], monocyte chemoattractant protein (MCP)-1/chemokine (C-C motif) ligand 2 (CCL2) [143,155] and other chemokines (e.g., CCL5, CCL3, CXCL9, CXCL10, CXCL11, IL-6, and IL-8) [114,143,146]. The IL-1β-mediated induction of COX2 was found to depend on nitric oxide production [149]. The pro-inflammatory cytokines were found to activate both the extrinsic and the intrinsic apoptotic pathways in β-cells [40,156,157]. Cottet et al. [156] observed that TNFα, but not IL-1β, activates caspase 8 in a β-cell line. Gunnet et al. [40] showed that exposure of β-cells or islets to the three cytokines IFNγ, IL-1β, and TNFα resulted in the dephosphorylation of Bad, activation of Bax-dependent mitochondrial stress, cleavage and activation of caspase 9 and caspase 3. The p53 upregulated modulator of apoptosis (PUMA) and the pro-apoptotic Bim were found to be upregulated in human islets and mouse β-cells after exposure to IL-1β/IFNγ or TNFα/IFNγ [157,158]. PUMA and Bim act upstream of Bax/Bak and induce the translocation of these proteins to mitochondria [159]. Silencing of PUMA or Bim partially protected β-cells from TNFα/IFNγ-induced apoptosis [157]. 

The pro-inflammatory cytokines downregulate the anti-apoptotic Mcl-1 in β-cells, thereby further increasing the susceptibility of the β-cells to the pro-apoptotic molecules of the intrinsic apoptotic pathway [160]. IL-1β increased iNOS expression, which is further enhanced by IFNγ in both rat and human islets [143,161]. In addition, IL-1β induces the expression of Death protein 5 (DP5)/Harakiri (Hrk) in rat islets [143] and rat INS-1 β-cell line [134], and IL-1β together with IFNγ induces ER stress in β-cells with phosphorylation of eukaryotic initiation factor 2α (eIF2α), induction of activating transcription factor 4 (ATF4) and upregulation of CCAAT/enhancer-binding protein (C/EBP) homologous protein (CHOP) [47,162,163]. Moreover, TNF superfamily member 10* (TNFSF10; TRAIL)* expression was increased in human β cells after exposure to IL-1β and IFNγ [45]. While TNFα increases the expression of the antiapoptotic X-linked inhibitor of apoptosis (XIAP), IFNγ represses this induction [125]. 

Nakayasu et al. [39] performed a comprehensive analysis of protein changes occurring after treatment of human islets with IL-1β and IFNγ. This study showed that cytokine treatment affected proteins related to NFκB signaling, cytokine-cytokine receptor interactions, apoptosis, antigen processing and presentation, and extracellular matrix [39]. Notable, IL-1β and IFNγ upregulated the expression of several interleukins (e.g., IL-11, IL-1α, IL-1β, and IL-32), chemokines (e.g., CCL5, CCL8, CCL13, CSF1, CXCL2,3,5,6,8,9,10,11, and CX3L1), various caspases (e.g., caspases 1, 4, 5, 7, 8, and 10), the receptor-interacting serine/threonine protein kinase 2 (RIPK2), the anti-apoptotic protein PUMA and the inducible nitric oxide synthase (iNOS) responsible for nitric oxide production [39]. The anti-apoptotic growth factors thrombospondin 1, connective tissue growth factor (CTGF), and osteopontin (SPP1) were downregulated by IL-1β and IFNγ [39]. Osteopontin protects β-cells from cytotoxic effects and prevents hyperglycemia [164]. Nakayasu et al. [39] further showed that IL-1β and IFNγ downregulated growth/differentiation factor 15 (GDF15, formerly known as macrophage inhibitory cytokine 1 [MIC-1]). Treating human islets with GDF15 prevented the apoptosis induced by IL-1β and IFNγ, and administration of GDF15 to non-obese diabetic (NOD) mice prevented the development of diabetes [39]. In a transcriptome analysis, Eizirik et al. [146] observed a significant downregulation of growth differentiation factor 10 (GDF10), fibroblast growth factor 17 (FGF17), and transforming growth factor β2 (TGFβ2) in human islets treated with IL-1β and IFNγ. 

RIPK2 (RIP2, CARDIAK) is involved in transmitting signals from nucleotide-binding oligomerization domain 1 (NOD1), NOD2, and Toll-like receptors (TLRs) to NFκB, resulting in the induction of cytokine production [165]. Caspase 1 is involved in the processing of pro-IL-1β and pro-IL-18 into mature inflammatory cytokines and was therefore initially named IL-1 beta converting enzyme [166,167,168]. Caspase 1, together with the apoptosis-associated speck-like protein containing a CARD (ASC) and the nucleotide-binding oligomerization domain, leucine-rich repeat, and pyrin domain-containing protein (NLRP) 3, forms the inflammasome, which is activated by several endogenous and exogenous stimuli (e.g., pathogen-associated molecular patterns (PAMPs) and damage-associated molecular patterns (DAMPs) leading to the activation of caspase 1 [167,168,169,170,171]. It means that it is not sufficient that pro-caspase-1 is transcribed, but it must also be activated. Caspase 4, which is also upregulated by cytokines in β-cells, is involved in the activation of caspase 1 [172]. Mitochondrial DNA from diabetic mice and reactive oxygen species (ROS) can activate caspase 1 [173,174,175]. RIPK2 has been shown to be an activator of pro-caspase-1 [176], resulting in the induction of neuronal cell death among others through caspase 1-mediated cleavage of Bid to truncated Bid (tBid) [177]. Overexpressing RIPK2 in MCF7 breast carcinoma cells resulted in apoptosis that was mediated through its caspase recruitment domain (CARD) [178]. The RIPK2-Caspase 1 signaling pathway is also involved in pyroptosis, a kind of lytic cell death caused by inflammation, also known as gasdermin-dependent cell death [169,179,180,181,182,183,184]. Caspase 1 cleaves gasdermin D to release a pore-forming domain that forms pores in the plasma membrane, leading to cell lysis [182]. NLRP3 deficiency prevented the development of T1D and improved glucose tolerance and insulin sensitivity in mice [175], which was associated with diminished T-cell activation, T helper 1 (Th1) differentiation, T cell chemokine expression, and pathogenic T cell migration to pancreatic islets [185]. Polymorphisms in the NLRP1 and NLRP3 genes have been associated with a predisposition to T1D [168,186,187].

Dad1, which is downregulated by the cytokines, regulates N-linked glycosylation, binds to the anti-apoptotic Mcl-1, and inhibits apoptosis [188,189]. Deletion of Dad1 in mice leads to aberrant embryonic morphology, impaired mesodermal development, and excessive apoptosis, ultimately resulting in lethality by embryonic day 10.5 [190,191,192]. These studies suggest an important role of Dad1 as a survival factor. Notably, Dad1 was upregulated in primary rat β-cells exposed to 10 mM glucose and 20 mM glucose compared to those exposed to 5 mM glucose [193].

The upregulation of A20 may be a mechanism to protect the β-cells from apoptosis [194,195,196]. Overexpression of A20 in islets increased the survival rate of allogeneic islet transplants by preventing NFκB signaling [197]. TLR signaling in immune cells might have both pro- and anti-diabetogenic effects affected by the gut microbiota [198,199,200]. TLR4 deficiency reduces macrophage infiltration into the islets [199]. TLR4 levels are upregulated in pancreatic islets of obese mice, and TLR4 knockout mice become less obese when fed with a high-fat diet [199]. TLR4 deficient β-cells isolated from mice fed with a high-fat diet showed improved glucose-stimulated insulin secretion and expressed lower mRNA levels of IL-6, TNFα, and MCP-1 [199]. Thus, upregulation of TLR4 on β-cells in response to fatty acids leads to increased cytokine and chemokine production, which promotes macrophage infiltration of the islets with resulting β-cell dysfunction [199]. Burrows et al. [198] observed that deletion of the TLR-associated Innate immune adaptor myeloid differentiation primary response gene 88 (MyD88) in NOD mice led to T1D development in germ-free, but not in germ-exposed, environments. They further observed that knocking out the TIR-domain containing adapter inducing IFNβ (TRIF) in the MyD88 knockout NOD mice led to T1D development under normal germ exposed conditions [198]. These observations suggest that TRIF, which acts downstream to TLR4, induces microbiota-induced tolerogenic pathways [198]. However, knocking down TLR2 in the MyD88 knockout NOD mice led to reduced T1D incidences in germ-free conditions, suggesting that TLR2 delivers pro-diabetic signals [198]. TLR2 knockout mice were less prone to streptozotocin-induced diabetes [200]. In an overexpressing study using 293 embryonic kidney epithelial cells, activation of TLR2 was found to induce apoptosis through activation of the MyD88-Fas-associated death domain protein (FADD)-caspase 8 and caspase 1 pathway [201]. Further studies are required to understand the contribution of cytokine-induced TLR2 expression to β-cell viability and function.

### 3.3. Cytokines and Growth Factors Promoting β-Cell Survival and Preventing Pro-Inflammatory Cytokine-Induced Apoptosis

The pro-apoptotic effect of the pro-inflammatory cytokines on β-cells can be antagonized by the anti-inflammatory cytokines IL-4, IL-6, IL-10, and IL-13, which activate Signal transducer and activator of transcription 3 (STAT3; IL-6 and IL-10) and STAT6 (IL-4 and IL-13) signal transduction pathways [43,202,203,204,205,206]. IL-4 promotes the production of protective regulatory Th2 cells [207,208]. IL-10, TGFβ, and IL-33 can prevent β-cell damage by suppressing the immune system and inducing immune tolerance [209]. Growth hormone protected β-cells from the deleterious effects of cytokines by activating STAT5 with a concomitant increase in the Bcl-xL/Bax ratio [210]. The cytokines also down-regulate the expression of the anti-apoptotic Mcl-1 [160]. Overexpression of Bcl-xL or Mcl-1 protects β-cells from cytokine-induced apoptosis [160,211]. Other factors that can protect β-cells from cytokine-induced cell death include islet neogenesis-associated protein (INGAP) and its active pentadecapeptide core [212], n-3 polyunsaturated fatty acids (n-3 PUFAs) [213], insulin [214], insulin-like growth factor 1 (IGF1) [215], IGF2 [216,217], hepatocyte growth factor [218], osteopontin [219], stromal cell-derived factor 1 (SDF-1) [220], and neutral ceramidase [221] (Table 1). The importance of IGF2 production from pancreatic mesenchymal cells in β-cell survival was demonstrated in a conditional IGF2 mouse model, where IGF2 deletion resulted in both acinar and β-cell hypoplasia [222]. Co-transplantation of islets with neural crest stem cells increased β-cell proliferation and improved islet function [223], which has been related to the secretion of nerve growth factor (NGF) [224,225]. Inhibition of NGF signaling increased basal insulin secretion but impaired glucose-stimulated insulin secretion [224].

Glucose at normal levels promotes the expansion and survival of β-cells [193,296,381,382,383], but it can also act as a stressor that induces β-cell dysfunction through glucotoxicity [384,385,386,387,388]. Glucose-mediated stimulation of β-cell growth and survival depends on the activation of the insulin receptor and insulin receptor substrate 2 [389]. Glucose increases the expression of prolactin (PRLR), growth hormone (GHR), cholecystokinin A (CCKAR), and glucose-dependent insulinotropic polypeptide (GIPR) receptors in primary rat β-cells [193]. 

A transcriptome analysis of genes altered following glucose treatment of a human β-cell line showed a rapid upregulation of the proconvertase PCSK1 involved in the proteolytic conversion of pro-insulin to insulin [390]. A similar upregulation of PCSK1 was observed in mouse islets exposed to glucose [384]. Transcriptome analysis of mouse islets exposed to high glucose showed upregulation of genes associated with enhanced respiration, ER stress, and oxidative stress [384]. Among the highly upregulated genes by high glucose is thioredoxin interacting protein (TXNIP; thioredoxin-binding protein 2 (TBP2)), which inhibits the antioxidant activity of thioredoxin (TRX), resulting in intracellular oxidative stress [391]. TXNIP is involved in the glucotoxic effects leading to β-cell death [387,392]. A proteomic analysis of glucose-treated human β-cells showed enrichment in proteins involved in translation, glycolysis, TCA metabolism, and insulin secretion [390]. The mTOR signal pathway was shown to be involved in the glucose-induced effects in human β-cells [390,393]. Bertolini et al. [227] observed that glucose increases the expression of activin B and its receptor ALK7 but downregulates activin A in mouse islets. This might be a feedback mechanism as activin B decreases glucose-stimulated Ca^2+^ influx through ALK7, while activin A increases the glucose-stimulated Ca^2+^ influx [227]. Glucose stimulation of mouse islets also leads to a transient induction of growth differentiation factor 5 (GDF5, also known as BMP14) and the transcription factor mesenchyme homeobox 2 (MEOX2) (unpublished data). Overexpressing of PDX1 in MIN6 β-cell line induced expression of both GDF5 and MEOX2 (unpublished data), suggesting that the expression of these genes is regulated by PDX1, which is a master regulator of β-cells [394,395,396]. GDF5 has been shown to form heterodimers with BMP2 and BMP4 [397], both of which modulate β-cell differentiation during embryonic development and regulate glucose-induced insulin secretion in adult islets (Table 1). The mesenchyme homeobox 2 (MEOX2) regulates vertebrate limb myogenesis [398,399] and is expressed in the vertebrate embryo in regions of epithelial–mesenchymal interactions [400]. MEOX2 expression has previously been shown to be expressed in MIN6 β-cells [401,402]. Further studies are required to understand the role of GDF5 and MEOX2 in β-cell survival and function.

## 4. Mesenchymal Stem Cells

Mesenchymal stem cells, also called multipotent stromal cells (MSCs), are adherent, spindle-shaped, fibroblast-like cells that can be isolated from various tissues, including the bone marrow, adipose tissue, and umbilical cord [403,404]. MSCs lack any markers of hematopoietic cells (e.g., CD34, CD45, CD19 and HLA-DR) and the endothelial marker CD31, but express CD105 (SH2 or endoglin), CD71, CD73, CD44, CD29, stem cell antigen-1, and CD90 (Thy-1) [405,406,407,408]. MSCs do not express the major histocompatibility complex II (MHC-II) or the co-stimulatory molecules B7-1, B7-2, CD40, and CD40L required for T cell activation, such that these cells cannot activate T lymphocytes, and rather most studies show that MSCs actually suppress T-cell proliferation and activity, and increase the proportion of T regulatory cells [409,410,411,412,413,414,415,416]. MSCs exhibit general immunosuppressive activities that are beneficial in the treatment of various autoimmune diseases [417,418,419,420]. MSCs can induce a T helper 1 (Th1) to T helper 2 (Th2) shift with reduced IFNγ secretion and increased IL-4 production [410]. MSCs suppress IFNγ secretion from IL-2-stimulated NK cells [410] and inhibit IL-15-induced NK cell proliferation and their production of IFNγ, TNFα and IL-10 [421]. MSCs modulate the activity and polarization of macrophages [422,423], dendritic cells [410,424,425,426,427,428,429], and neutrophils [430,431,432], thus contributing to the homeostasis of the inflammatory microenvironment. There is also a crosstalk between MSCs and immune cells with mutual regulation [420].

MSCs are characterized by high self-renewability and multipotency with the ability to differentiate into various cell lineages, including osteoblasts of the bone, myoblasts of the muscle, chondrocytes of the cartilage, and adipocytes of the adipose tissue [61,403,408,433,434,435]. There are several lines of evidence that MSCs are formed from the differentiation of perivascular pericytes [407,434,436,437,438]. Tissue-specific MSC functions have been suggested, where the local microenvironment may influence their plasticity [439].

Many protocols have been developed to differentiate MSCs into functional insulin-producing β-cells by exposing the cells to chemical and biological factors or by genetic manipulation introducing the PDX1 gene, which is a master regulator in pancreas organogenesis [232,437,440,441,442,443,444,445,446,447,448,449,450,451,452,453,454,455,456,457,458,459]. A common dominator for the different differentiation protocols is the sequential exposure of MSCs to different combinations of growth factors (e.g., EGF, bFGF, betacellulin, activin A, HGF, extendin-4, insulin) chemical compounds (e.g., nicotinamide), and B27 supplement (containing among others insulin, biotin, vitamin E, Vitamin A, selenium, putrescine, transferrin, catalase, superoxide dismutase, triodo-L-thyronine, linoleic and linolenic acids) for different time periods [441,442]. Nicotinamide enhances the differentiation of human pancreatic cells and promotes the expression of insulin, glucagon, and somatostatin [460]. It also induces MAF1 and insulin promoter activity in a rat β-cell line [461]. Nicotinamide protects β-cells from oxidative stress, by virtue of its antioxidant properties [462]. Gao et al. [231] used a five-step protocol to differentiate β-cells from MSCs. This protocol included an initial induction using the demethylation agent 5-aza-2′-deoxycytidine, followed by incubation in a low glucose medium. This was followed by serial incubations with activin A, all-trans retinoic acid (ATRA), and bFGF together with B27, insulin, transferrin, selenite, and nicotinamide. Scuteri et al. [463], however, observed that incubating rat MSCs with rat islets was sufficient to differentiate the MSCs into PDX1-expressing and insulin-secreting cells, suggesting that factors secreted by islets (e.g., insulin) can affect the phenotype of the interacting MSCs. Although the direct effect of insulin as a single differentiation factor on MSCs has not yet been documented, insulin has been shown to increase glucose uptake and GLUT4 translocation in MSCs [464]. The ability of MSCs to adhere to the islets [463] and to home to the islets following transplantation, where it improves β-cell function, suggests a mutual interaction between the two cell types. Similar to MSCs, human liver stem-like cells (HLSC) have been shown to generate insulin-producing 3D spheroid structures in vitro that could restore normoglycemia in streptozotocin-induced diabetic mice [465,466]. 

MSCs have been shown to improve the medical conditions of a variety of immune-mediated diseases, including graft rejection, graft-versus-host disease, rheumatoid arthritis, systemic lupus erythromatosis, Crohn’s disease, colitis, osteoarthritis, multiple sclerosis, experimental autoimmune encephalomyelitis (EAE), and psoriasis [467,468,469,470,471,472,473,474,475]. Moreover, MSCs can promote wound healing and tissue regeneration [404,476,477,478]. Human MSCs have been shown in various settings to have a beneficial role in diabetes [479,480,481]. There is accumulating evidence that MSCs have beneficial effects on insulin-producing β-cells and islet survival, and co-transplantation of islets with MSCs increases the survival of the islet grafts, which will be further discussed below. MSC transplantation has the advantage of being well tolerated by the patients without any apparent toxicity [482,483,484,485,486,487,488,489,490], although some occasionally adverse effects have been noted, such as gastrointestinal and skin disorders [480].

After transplantation of MSCs, these cells can home to injured tissues and promote tissue regeneration, among others, by differentiating into various cellular phenotypes, providing cytokines, chemokines, growth factors, and other bioactive factors, enhancing the proliferation of stem cells and progenitors of the tissue and suppressing immune responses [404,434,468,469,478,491]. Since many of the MSC functions are caused by secretory molecules, A. I. Caplan suggested renaming the cells to “medicinal signaling cells” [434]. 

Using luciferase-expressed MSCs, Lin et al. [492] observed that intra-arterially injected MSCs specifically engraft to sites of injury caused by local irradiation of mice. Similarly, Chapel et al. [493] observed that green fluorescence protein (GFP)-labeled MSCs home to injured tissues in a model of total body irradiation of macaques (*Macaca fascicularis*). By transplanting MSCs from male rats into female rats, Boumaza et al. [494] found that MSCs can also be found in the pancreas. DiR-labeled human umbilical cord-derived MSCs were found to accumulate in the lung, liver, spleen, and pancreas for up to 7 days after intravenous injection into streptozotocin-induced diabetic mice [495]. The homing of the MSCs to the pancreas is believed to have a supportive role in islet regeneration and survival. 

### 4.1. In Vitro Evidence for β-Cell Supporting Roles of MSCs

Cultivation of human islets in vitro leads to loss of function, dedifferentiation, senescence, apoptosis, and necrosis [63,78,340,379,496,497,498]. The isolation process also reduces the number of viable islets. Single-cell transcriptional analysis of human islets obtained 3–6 days post-isolation detected insulin-positive cells with reduced expression of β-cell genes with concomitant elevated levels of progenitor markers, indicating that an early ex vivo dedifferentiation process has taken place [499]. There is, therefore, a need to develop proper culture conditions to maintain islet function both for in vitro studies and for islet preservation prior to islet transplantation. Several studies have shown that co-culture of islets with MSCs prevents the ex vivo loss of function of islets (Table 2) [463,500,501]. These observations suggest that MSCs provide factors that sustain β-cell function and survival and prevent the spontaneous dedifferentiation that usually occurs in culture. 

Yeung et al. [348] observed that human MSCs protected human islets from the destruction caused by the pro-inflammatory cytokines IFNγ, TNFα, and IL-1β. The MSC-mediated cytoprotection was attributed to the secretion of HGF and metalloproteinases 2 and 9 [348]. MMP-2 and MMP-9 have been shown to contribute to the immunosuppressive function of MSCs by reducing the surface expression of IL-2R (CD25) on T cells [502]. MMP9 knockout mice showed normal development of pancreata and islets but had an impaired response to glucose load in vivo, and MMP9 knockout islets secreted a reduced amount of insulin in response to glucose [503]. This suggests that extracellular matrix turnover is important for releasing paracrine factors from the matrix.

**Table 2:** In vitro evidence for β-cell supporting roles of MSCs.

In Vitro Effects of MSCs on Islet Function	References
Human MSCs cultures supported human islet function in an indirect co-culture system where the islets were separated from the MSC monolayer by a membrane. Islets exposed to MSC-secreted factors showed a decreased ADP/ATP ratio compared to islet monoculture, and their insulin-secretion function was improved. The conditioned medium of MSC contained HGF, TGFβ, IL-6, and VEGF-A. The co-culture medium had lower TNFα and IFNγ content than that of the islet monocultures.	[504]
Mouse islets cultured with human umbilical cord-derived MSCs showed increased viability, reduced ADP/ATP content, and increased glucose-stimulated insulin secretion. Islets cultured with MSCs showed increased expression of the anti-apoptotic XIAP (X-linked inhibitor of apoptosis protein).	[379]
Rat islets cultured in the absence of MSCs gradually lost their structural integrity and insulin-secretion function within the first three weeks. Rat islets incubated with rat MSCs showed preserved morphology and functional insulin secretion. The islets were surrounded by the MSCs. The MSCs prevented the production of TNFα and MCP-1 from the islet, while the TIMP-1 and VEGF levels were higher in the co-culture than in the islet monocultures.	[505]
Human islets were protected from IL-1β-induced cell death by human MSCs overexpressing hepatocyte growth factor (HGF) and interleukin-1 receptor antagonist (IL-1Ra).	[506]
Human bone marrow-derived MSCs protected human islets from apoptosis induced by the combined treatment of TNFα, IL-1β, and IFNγ.	[507]
Rat islets co-cultured 38 days together with rat adipose-derived MSCs or bone marrow (BM)-derived MSCs showed significant improvement in basal insulin secretion levels compared to islets cultivated alone. Indirect incubation of streptozotocin-damaged rat islets with rat bone-marrow-derived MSCs resulted in the survival of the islets.	[508,509]
Cultivation of islets on MSC monolayers retained their insulin-producing function and prevented the dedifferentiation of islets occurring in culture.	[63]
Rat MSCs prolonged the survival of rat islets in culture and increased glucose-stimulated insulin secretion in comparison to islets alone. MSCs in contact with pancreatic islets differentiated into cells that express PDX1 and secrete insulin. MSCs adhered to the pancreatic islets.	[463]
Treatment of streptozotocin-injured mouse islets in vitro with the conditioned medium from bone marrow-derived MSCs increased the activation of AKT and ERK1/2 in the islets. The MSC-conditioned medium increased proliferation of β-cells in injured islets in an AKT-dependent manner.	[510]
Adipose tissue-derived MSCs from mice reduced the secretion of IFNγ, IL-2, and IL-17, while increasing the secretion of TGFβ, IL-4, IL-10, and IL-13 by PHA- and islet lysate-induced T cell stimulation. The MSCs protected mouse islets from the deleterious effects of reactive splenocytes in a co-culture setting.	[511]
Human islets that have been grown together with human bone marrow-derived MSCs for 3 days showed improved glucose-stimulated insulin secretion, which was further improved by addition of theophylline. The beneficial effect of MSCs was dependent on direct contact between the islets and MSCs and required the intact microstructure of the islets. Neutralizing antibodies to N-Cadherin that is expressed in the MSCs inhibited the beneficial effects of MSCs.	[512]
Rat islets encapsulated in alginate microcapsules together with MSCs and extracellular matrix proteins of the rat pancreas showed improved insulin secretion when compared to islets alone.	[513]
Human umbilical cord-derived MSCs attenuated high glucose-induced oxidative stress of rat INS-1 β-cells. The MSCs protected rat INS-1 β-cells from high glucose-induced injury and prevented the high glucose-mediated impairment in glucose-stimulated insulin secretion. MSCs increase the expression of NRF2 (Nuclear factor erythroid 2-related factor 2) and HO-1 (heme oxygenase-1) in rat INS-1 β-cells, and the knockdown of NRF2 in INS-1 abolished the protective activity of MSCs.	[495]

### 4.2. In Vivo Evidence for β-Cell Supporting Roles of MSCs

The use of MSCs in treating T1D has been shown to be effective in regulating fibrosis and tissue regeneration [48]. Co-transplantation of MSCs with pancreatic islets is more effective than islet transplantation alone in controlling glucose serum levels in diabetic animal models (Table 3). Repeated bone marrow transplantations into mice with experimental diabetes restored normoglycemia and normalized the morphology of the pancreas [514]. Systemic administration of MSCs into diabetic mice or rats resulted in pancreatic islet regeneration, increased endogenous insulin production, reduced blood glucose levels, reduced pancreatic inflammatory processes, induction of regulatory T cells, and prevention of renal damage [494,515,516]. Although some studies showed that the paracrine function of MSCs contributes to the beneficial effects of MSCs, the efficiency of MSC conditioned medium is far less efficient than MSC cell transplantation (Table 3). The beneficial effects of MSCs were especially observed when MSCs were co-transplanted with islets into diabetic animals (Table 3).

Ianus et al. [517] observed that GFP-expressing bone marrow-derived cells that have been transplanted into lethally irradiated mice have populated Langerhans islets four to six weeks after transplantation. The GFP-positive cells isolated from the islets were found to express insulin, GLUT2, and various β-cell specific transcription factors, including PDX1 (pancreatic and duodenal homeobox 1; formerly known as Ipf1—Insulin promotor factor-1) and PAX6 (Paired box protein 6) [517]. These GFP-positive cells of the islets also responded to glucose-dependent and incretin (exendin 4)-dependent insulin secretion [517]. These findings indicate that bone marrow stem cells have the ability to differentiate into insulin-producing cells, which, in part, rely on the interaction with islets. Further studies with GFP-expressing bone marrow cell transplants into diabetic mice showed increased proliferation of insulin^-^ PDX1^+^ cells, NGN3^+^ cells, and insulin^+^ glucagon^+^ cells with stem cell characteristics in the islets [518], suggesting a pancreatic regeneration role of MSCs. The mobilization of transplanted bone marrow cells to the islets was essential for pancreatic regeneration [519].

**Table 3:** In vivo evidence for β-cell supporting roles of MSCs in animal models.

In Vivo Effects of MSCs on Islet Function in Animal Models	References
Bone marrow-derived stem cells reduced hyperglycemia in mice that have been made diabetic by streptozotocin. The stem cells induced proliferation of pancreatic β-cells and led to increased islet insulin content.	[520]
Transplantation of human MSCs into streptozotocin-induced diabetic mice caused an increase in pancreatic islets and β-cells producing mouse insulin with concomitant reduced blood glucose levels.	[521]
Human bone marrow stromal cells (MSCs) transfected with PDX1, NEUROD1, and NGN3 expressed insulin in vitro that was not regulated by glucose. Transplantation of these modified MSCs under the kidney capsule of streptozotocin-induced diabetic mice reduced blood sugar levels.	[522]
Bone marrow-derived MSCs transplanted into streptozotocin-induced diabetic rats reduced blood glucose levels and increased the rat body weight. Some of the transplanted MSCs have differentiated into insulin-producing cells. There were many small islets in the pancreas of the MSC-treated rats.	[523]
Transplantation of MSCs into streptozotocin-induced diabetic mice reduced the blood glucose levels, reaching nearly euglycemic values after a month, which lasted for more than 2 months. There was an increase in pancreatic islets concomitant with reduced albuminuria and normal morphology of the kidney glomeruli. This was in contrast to untreated diabetic mice that presented glomerular hyalinosis and mesangial expansion.	[515]
Treatment of diabetic NOD mice with MSCs from BALB/c mice reversed hyperglycemia in most of the mice.	[516]
Transplanting rat islets together with MSCs reduced the amount of islets required to get normoglycemia. Islets transplanted with MSCs showed a higher number of capillaries than those transplanted without MSCs.	[524]
Mouse MSCs prevent the rejection of allogeneic islet grafts by secreting matrix metalloproteinases-2 and -9 (MMP2 and MMP9), which reduce CD25 expression on responding T lymphocytes. MSCs prolonged the survival of allogeneic islet grafts.	[502]
Repeated injections of MSCs into streptozotocin-induced diabetic rats resulted in lower blood glucose levels increased insulin serum levels. Peripheral T cells of the MSC-treated diabetic rats showed a shift toward IL-10/IL-13 production and higher frequencies of CD4^+^/CD8^+^ Foxp3^+^ regulatory T cells.	[494]
Co-transplantation of syngeneic rat islets with rat bone marrow-derived MSCs into omental pouch of streptozotocin-induced diabetic rats resulted in sustained normoglycemia. Transplantation of allogenic rat islets together with MSCs together with short-term immunosuppression with cyclosporin A, increased islet graft survival and insulin expression, and induced normoglycemia. The transplantation of allogeneic islets with.MSCs led to reduced production of IFNγ and TNFα, while an increased secretion of IL-10 from T cells.	[525]
Mouse MSCs suppressed diabetogenic T cell proliferation via PD-L1 and suppressed the generation of inflammatory dendritic cells. MSC treatment of type 1 diabetes in NOD mice resulted in long-term reversal of hyperglycemia.	[526]
When Lewis rat islets were transplanted together with rat MSCs into streptozotocin-diabetic syngeneic recipients or NOD-mice, a lower number of islets were required to achieve diabetes reversal. The islets transplanted together with MSCs showed improved vascularization, and islets were surrounded by MSCs.	[527]
Transplantation of mouse islets that have been co-cultured with MSC-conditioned medium into diabetic mice led to lower blood glucose levels and increased blood vessel formation. The MSC-conditioned medium contained IL-6, IL-8, VEGF, HGF, and TGFβ.	[379]
Transplantation of mouse islets together with mouse bone marrow cells resulted in enhanced islet graft vascularization, reduced blood glucose level, and increased serum insulin level.	[528]
Transplantation of mouse islets with mouse adipose tissue-derived MSCs promoted islet graft survival and insulin function of the graft in streptozotocin-induced diabetic mice and reduced the islet mass required for reversal of hyperglycemia.	[529]
Co-transplantation of mouse islets with mouse MSCs led to better reduction of blood glucose levels in streptozotocin-induced diabetic mice in comparison to mice injected with islets alone. The islets co-transplanted with MSCs showed better vascularization. Transplantation of MSCs alone did not revert hyperglycemia in the diabetic mice.	[530]
Transplantation of human islets together with human bone marrow-derived mesenchymal stem cells (hBMSCs) overexpressing human HGF and human IL-1Ra under the kidney capsule of streptozotocin-induced diabetic non-obese diabetic/severe combined immunodeficient (NOD-SCID) reversed diabetes and reduced the number of islets required to achieving normoglycemia.	[506]
Adipose tissue-derived stem cells that have been pretreated with a mixture of hyaluronic acid, butyric acid, and retinoic acid improved the islet graft revascularization. The treated adipose tissue-derived stem cells produced higher levels of VEGF and HGF.	[531]
Human bone marrow mesenchymal stem cells (MSCs) overexpressing vascular endothelial growth factor (VEGF) reversed hyperglycemia induced by streptozotocin in NOD/SCID mice. This effect was related to a better survival of β-cells. The MSC overexpressing VEGF also differentiated into vessels and β-cell-like cells.	[532]
Transplantation of rat bone marrow-derived MSCs into the pancreas of streptozotocin-induced diabetic rats resulted in reduced blood glucose levels and increased body weight. Similar results were obtained when the MSCs were injected into the head of the pancreas, the tail of the pancreas, or the whole pancreas.	[533]
Human bone-marrow-derived MSCs expressing high aldehyde dehydrogenase (ALDH) activity improved systemic hyperglycemia in streptozotocin-treated NOD/SCID mice and augmented insulin secretion by increasing islet size and vascularization. In vitro expansion of human bone-marrow-derived MSCs prior to transplantation reduced the capacity to diminish blood glucose levels in streptozotocin-treated NOD/SCID mice at two-week intervals.	[534]
Infusion of MSCs into high-fat diet/streptozotocin-induced T2D diabetic rats ameliorated hyperglycemia, reduced insulin resistance in peripheral tissue, and promoted β-cell function when delivered at the early phase (7 days after diabetes induction) but not at the later phase (21 days after diabetes induction). The MSCs promoted the recovery of streptozotocin-induced liver and pancreas damage. MSC infusion increased GLUT4 expression and increased insulin receptor substrate 1 (IRS1) and Akt phosphorylation in skeletal muscle, adipose, and liver tissues.	[535]
Transplantation of human umbilical cord-derived MSCs that have been differentiated into insulin-producing cells into the liver of streptozotocin-induced diabetic mice resulted in reduced serum glucose levels.	[536]
Co-transplantation of human bone marrow-derived MSCs together with human islets into diabetic humanized NOD SCID gamma (NSG) mice was more efficient than islet transplantation alone in improving blood glucose and serum insulin and C-peptide levels. The MSCs increased the proportion of immunosuppressive regulator T cells and protected the islets from apoptosis induced by pro-inflammatory cytokines.	[507]
Rats that have been made diabetic by high-fat diet together with streptozotocin administration became normoglycemic after at least three times of infusion of bone marrow-derived MSCs. Serum concentrations of insulin and C-peptide were increased after the serial MSC infusions.	[537]
Transplantation of human bone marrow-derived MSCs that have been differentiated into insulin-producing cells into streptozotocin-induced diabetic nude mice maintained euglycemia for 3 months. Bone marrow-derived MSCs from both diabetic and healthy human subjects could be differentiated into insulin-producing cells.	[443]
Cytoprotection of rat pancreatic islets by human adipose-derived stem cells was increased when fibroblast growth factor-2 (FGF2) was incorporated into the fibrin gel used for the subcutaneous transplantation. Some of the stem cells had differentiated into insulin-producing cells.	[538]
Rat islets co-transplanted with adipose-derived mesenchymal stromal cells (MSCs) under the kidney capsule of streptozotocin-induced diabetic rats resulted in better recovery than islet transplants alone. The MSC co-transplanted with islets had differentiated into insulin-producing cells.	[508]
In vitro cultivation of mouse islets prior to transplantation into streptozotocin-indued diabetic mice reduces their ability to reverse diabetes. However, co-cultivation of freshly isolated islets on kidney-derived MSCs improved islet graft function.	[63]
Intravenous infusion of MSCs after intra-hepatic islet transplantation or co-transplantation of MSCs together with islets under the kidney capsule resulted in improved glucose homeostasis in a streptozotocin-induced diabetic mouse model. The co-transplantation with MSCs resulted in reduced islet apoptosis.	[539]
Injection of bone marrow-derived MSCs into streptozotocin-induced diabetic mice reduced blood glucose levels increased both the size and number of islets with a concomitant increase in β-cell mass.	[510]
Subcutaneous transplantation of rat islets that have been seeded on rat MSC sheets into streptozotocin-induced severe combined immunodeficiency (SCID) diabetic mice resulted in normoglycemia in the recipient mice.	[540]
Infusion of Wharton’s jelly-derived MSCs to NOD mice at the onset of diabetes led to normal glucose homeostasis within 6–8 days that lasted for 6 weeks. Infusion of Wharton’s jelly-derived MSCs to NOD mice prior to onset of diabetes resulted in an 8-week delay in the onset of diabetes. The MSC infusion resulted in higher fasting C-peptide levels, higher frequencies of CD4^+^CD25^+^Foxp3^+^ regulatory T lymphocytes, and lower levels of IL-2, IFNγ, and TNFα.	[541]
Intrapancreatic injection of allogeneic adipose tissue-derived MSCs into streptozotocin-induced diabetic mice decreased blood glucose levels and improved glucose tolerance. However, the MSCs only remained in the tissue for a few days.	[542]
Retro-orbital venous sinus injection of GFP-overexpressing undifferentiated Wharton’s jelly-derived MSCs into NOD mice resulted in lower blood glucose levels, improved glucose tolerance, and higher survival rates. Human C-peptide and insulin could be detected in the serum of the mice. The MSC treatment resulted in reduced levels of auto-aggressive T cells and increased levels of regulatory T cells. The splenocytes expressed lower mRNA levels of IFNγ, IL-1β, TNFα, MCP-1, while higher mRNA levels of IL-4, IL-10, IL-17, and FoxP3. Fluorescent islet-like cell clusters in the pancreas could be observed whose origin was from the human MSCs. The undifferentiated MSCs differentiated into insulin-producing cells in vivo.	[543]
Co-transplantation of allogeneic islets together with autologous MSCs into streptozotocin-induced diabetic mice delayed islet rejection and increased long-term graft function in 30% of the mice. The MSCs need to be transplanted together with the islets since systemic delivery of MSCs did not have any protective effect.	[544]
Four weeks after infusion of MSCs, these cells were found to accumulate in the pancreas of diabetic NOD mice. Infusion of MSCs reduced insulitis while increasing the amount of splenic regulatory T cells. Following MSC infusion, the plasma levels of IFNγ and TNFα were reduced, while those of TGFβ1 and IL-10 were increased. The beneficial effects of MSCs were improved by Liraglutide, a long-acting GLP-1 analog.	[545]
Streptozotocin-induced diabetic mice transplanted with a combination of syngeneic adipose tissue-derived MSCs and allogeneic islet grafts showed reduced blood glucose levels and decreased pro-inflammatory cytokine (IFNγ, IL-17A) levels, while increased regulatory cytokine (TGFβ, IL-4) levels in mononuclear blood cells.	[546]
Transplantation of betatrophin-overexpressing human adipose-derived MSCs into streptozotocin-induced diabetic mice had a better effect on blood glucose levels than regular adipose-derived MSCs. In vitro co-culture of betatrophin-overexpressing human adipose-derived MSCs with human islets induced islet cell proliferation and improved glucose-stimulated insulin secretion. The betatrophin-overexpressing human adipose-derived MSCs had stronger anti-inflammatory and anti-apoptotic effects on islets than the regular adipose-derived MSCs.	[547]
MSCs that have been exposed to hypoxia express higher levels of VEGF, IL-6, MCP1, and MMP9. These hypoxia-treated MSCs increased islet grafts in a model of streptozotocin-induced diabetes in mice with lowering of the blood glucose level.	[548]
Injection of adipose tissue-derived MSCs from healthy, T2D diabetic or *db*/*db* mice into high-fat diet and streptozotocin-induced T2D diabetic mice improved insulin sensitivity and reduced β-cell death.	[549]
Transplantation of rat islets together with rat MSCs into streptozotocin-induced diabetic rats increased serum insulin levels and reduced the number of islets required for reducing blood glucose level and survival. The TNFα serum level was significantly reduced in the diabetic rats following MSC—islet co-transplantation.	[550]
Adipose tissue-derived MSCs reduced the number of islets required to achieve normoglycemia. The MSCs increased islet revascularization and the expression of angiogenic factors such as HGF and angiopoietin-1.	[551]
Administration of human telomerase (hTERT)-overexpressing MSCs into 50% pancreatectomized NMRI nude mice resulted in increased proliferation of pancreatic β-cells. The MSCs increased EGF, GLUT2, INS1, and INS2 mRNA levels in the pancreas while reducing the mRNA levels of IFNγ and TNFα.	[552]
Repeated administration of the conditioned medium of 2D and 3D human umbilical cord-derived MSCs cultures into streptozotocin-induced diabetic rats increased the rate of β-cells in the islets and increased the serum insulin and C-peptide levels. However, the conditioned medium was not sufficient to reduce the blood glucose levels in the diabetic rats. The conditioned medium increased the percentage of splenic regulatory T cells.	[553]
Transplantation of insulin-producing cells (IPCs) differentiated from human HLA-A2-negative adipose tissue-derived MSCs into the kidney capsule of streptozotocin-induced diabetic humanized mice normalized the blood glucose levels with detectable circulating human, but not mouse, insulin.	[451]
Human umbilical cord-derived MSCs in diabetic mice supported β-cell function with better insulin secretion performance. There was an increase in both the size and number of islets, and the ratio of insulin-producing β-cells was increased.	[495]
The combined treatment of streptozotocin-induced diabetic rats with bone-marrow-derived MSCs and hesperetin (a citrus flavonoid) improved glucose, insulin, and C-peptide levels.	[554]

Most of the human studies involved the transplantation of MSCs alone or in combination with mononuclear cells (MNCs) to T1D or T2D patients, which showed promising beneficial effects in terms of improved glucose homeostasis and reduced insulin requirements (Table 4). Autologous MSC transplantation in recent onset T1D patients has been shown to improve glycated HbA1c and C-peptide levels, preserve β-cell function, and shift serum cytokine patterns from pro-inflammatory cytokines to anti-inflammatory cytokines [482,488]. These beneficial effects of MSCs on islets are combined effects of direct supportive effect of MSCs on islet function and regeneration, anti-inflammatory activities, vascularization, protection of islets from hypoxic damage, and differentiation of MSCs into insulin-producing cells [517,538,555,556,557]. So far, MSC transplantation has mainly been performed in diabetic patients without co-transplantation with islets (Table 4). It is expected that islet co-transplantation with MSCs should improve the outcome in humans, as has been shown in animal studies (Table 3). 

## 5. The Paracrine Function of MSCs

**Figure 1:**
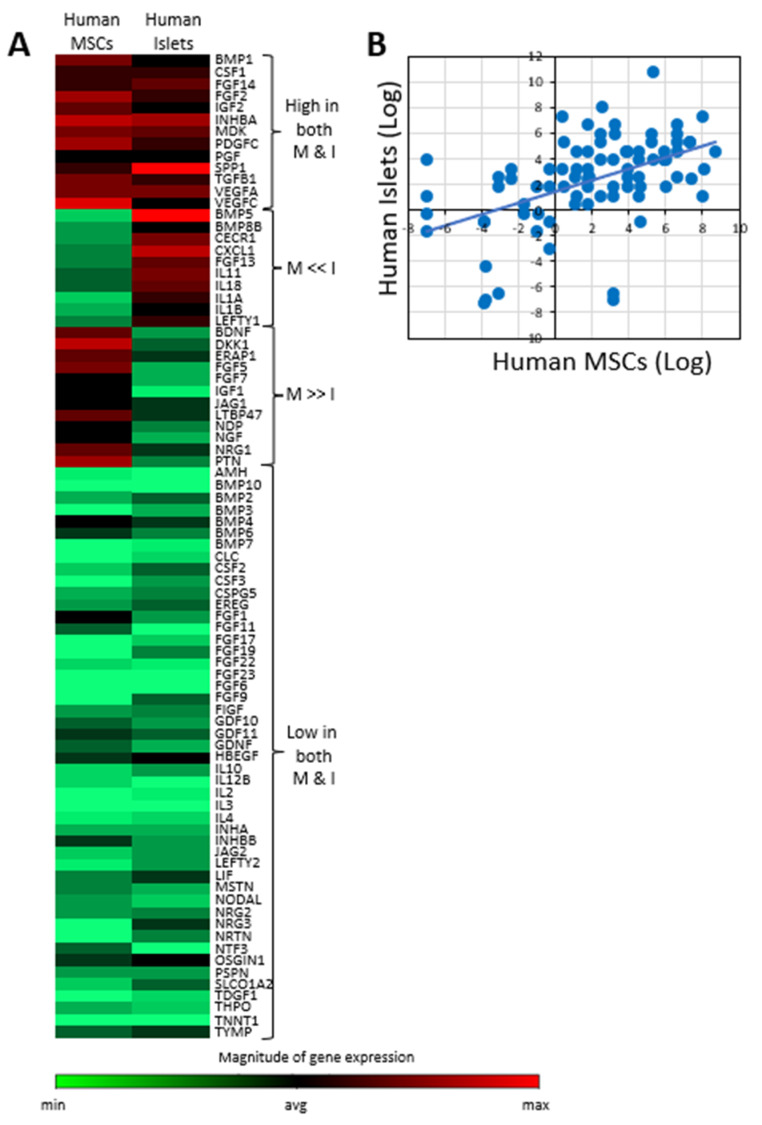
(**A**). Heatmap visualization of growth factor and cytokine mRNA expression in human bone marrow-derived MSCs (Lonza, Catalog number PT-2501, Walkersville, MD, USA) and human islets (obtained from PRODO Laboratories Inc., Irvine, CA, USA) using the Human growth factor RT Profiler PCR Array PAHS-041A (Qiagen, MD, USA). M = Mesenchymal stem cells. I = Islets. (**B**). A clusterogram of growth factor genes expressed in human islets versus human MSCs.

**Figure 2:**
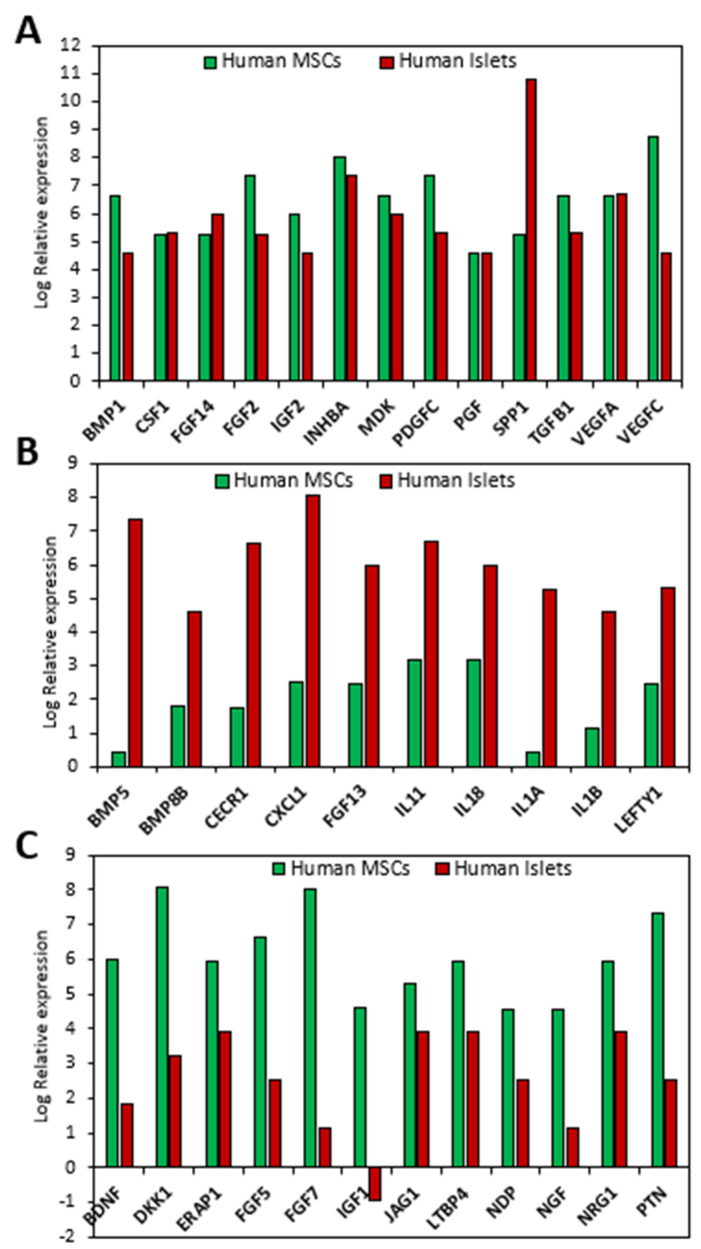
(**A**–**C**). mRNA expression in human bone marrow-derived MSCs and human islets for the indicated genes as determined using the Human growth factor RT Profiler PCR Array as shown in Figure 1.

Several proteomic studies have been performed to clarify the composition of the secretome of MSCs [580,581,582]. The complex secretome of MSCs consists, among others, of cytokines, chemokines, growth factors, extracellular matrix components, and extracellular vesicles [379,413,434,439,463,491,555,580,583,584,585,586,587,588,589,590,591,592,593,594] (Table 5, Figure 1 and Figure 2, and Supplementary Figure S1). Some of these factors are expressed in sub-population of MSCs, and their expression levels can be affected by interaction with other cell types, by cytokines, and by hypoxia [424,595,596,597,598,599,600]. Moreover, the secretome of rat adipose-derived MSCs differs from that of rat bone-marrow-derived MSCs [601], and there are differences in the MSC secretome between different species. Despite these differences, outstanding is the secretion of VEGF, angiopoietin-1, angiogenin, activin A, FGF7, HGF, TGFβ1, stromal cell-derived factor 1 (SDF1), platelet-derived growth factor (PDGF), MCP1, TSP1, TSG14, TIMP1, IL-8, IL-6, CXCL1, and IGFBP3 [379,494,509,511,524,581,584,585,602]. Rat adipose tissue-derived MSCs express higher levels of IL-1α, IL-6, CXCL1, CCL20, and CCL2 than rat bone-marrow-derived MSCs, while the latter express higher levels of Wnt1 inducible signaling pathway protein 2 (WISP2), osteomodulin (OMD), TGFβ2, and BMP4 [601].

Some of the MSC-produced factors support β-cell function and survival, as mentioned in Table 1. Trophic factors produced by MSCs with a beneficial effect on β-cell survival and function include VEGF [377,524,585], CNTF [270,603], HGF [348], von Willebrand factor [524,525,527,528,603], SDF-1 [220], and IL-6 [379]. SDF1 (CXCL12) has a positive effect on β-cell differentiation and survival besides causing immunosuppression and promoting wound repair [220,371,372,604]. MSCs promote angiogenesis by virtue of the secretion of bFGF and VEGF as well as certain cytokines such as IL-1, IL-6, and M-CSF (CSF1) [605]. The pro-angiogenic VEGF, which is highly expressed both in MSCs [379] and islets [263], has been shown to act as a survival factor for human islets [377]. Human islets that have been cultured in MSC-conditioned medium expressed higher levels of anti-apoptotic signal molecules (X-linked inhibitor of apoptosis protein (XIAP), Bcl-xL, Bcl-2, and heat shock protein-32 (HSP32)) and increased expression of vascular endothelial growth factor receptor 2 (VEGFR2) [379]. Altogether, the production of several different growth factors by MSCs may explain how co-administration of islets with MSCs can improve the efficiency of islet transplantation [63,606]. 

An important property of MSCs is their ability to survive under hypoxic conditions [598,607,608,609]. Exposure of MSCs to hypoxic conditions enhances the expression of VEGFA, PDGF, bFGF, IL-10, IL-6, IL-8, RANTES, MCP-1, TGFβ and MMP9 [548,599,600,608,610]. Analogously, stimulation of MSCs with TNFα increases their secretion of the pro-angiogenic cytokines IL-6 and IL-8 and the chemokines CXCL5, CXCL6, CXCL10, and MCP1 [582,611] as well as the growth factors VEGF, HGF and insulin-like growth factor I (IGF-I) [612]. IL-6, IL-8, and MCP1 are also involved in monocyte chemoattraction [582]. The production of HGF by adipose tissue-derived MSCs is stimulated by bFGF and EGF [613]. TNFα-stimulated MSCs promote endothelial progenitor cell homing and angiogenesis [611]. The production of heme oxygenase (HO)-1 by MSCs protects islets from injury caused by hypoxia and reoxygenation [614]. Pro-inflammatory cytokines reduce HO-1 expression in rat islets, which is prevented by the co-culture with human MSCs [615]. Thus, the cytoprotective and angiogenetic effects of MSCs are enhanced under hypoxia and inflammatory conditions.

The production of TGFβ1, indoleamine 2,3-dioxygenase (IDO), prostaglandin E_2_ (PGE_2_), IL-10, HGF, metalloproteinases, HO-1, tumor necrosis factor-induced protein 6 (TSG6), and nitric oxide (NOˑ) by MSCs has been associated with their immunosuppressive properties [62,348,502,616,617,618,619,620,621,622]. Some of these factors are induced in MSCs by inflammatory cytokines. For instance, IFNγ induces MSC expression of IDO, which catalyzes the conversion of tryptophan to kynurenine, resulting in tryptophan depletion and suppression of T lymphocyte function by metabolites of kynurenine [618,623]. Nitric oxide (NO·) production by MSCs, which is also induced by IFNγ, suppresses STAT5 phosphorylation and T cell proliferation [620]. The combined treatment of MSCs with both IFNγ and IL-1β induces a higher expression of IDO and PGE_2_ than each cytokine alone, resulting in better immunosuppressive activities as demonstrated in a murine colitis model [624]. IFNγ also induces Programmed death-ligand 1 (PD-L1, B7-H1, CD274) expression on MSCs that further suppresses T cell proliferation [625]. The secretion of the chemokines CCL2 and CXCL12 by MSCs contributes to the polarization of macrophages into IL-10-producing cells involved in anti-inflammatory responses [626]. PD-L1 and PD-L2 expression is upregulated in MSCs under inflammatory conditions, which contribute to immunosuppression by interacting with PD-1 receptors on T lymphocytes [442,627,628]. Stimulation of MSCs with IFNγ and TNFα induces the secretion of PD-L1 that suppresses the activation of CD4^+^ T cells and downregulates IL-2 secretion [628]. Other studies show that MSCs increase a subpopulation of CD4^+^ that produces IFNγ and IL-10, an effect that depends on IFNγ-stimulation of the MSCs [629].

Extracellular vesicles produced by MSCs have also been shown to contribute to their immunosuppressive activities [583,630,631]. Among others, these vesicles prevent antigen uptake by immature dendritic cells and the maturation of dendritic cells with reduced expression of the activation markers CD83, CD38, and CD80 and decreased secretion of the pro-inflammatory cytokines IL-6 and IL-12p70 while increased production of the anti-inflammatory cytokine TGFβ [632]. By using dendritic cells differentiated from CD14^+^ cells isolated from T1D patients, MSC-derived extracellular vesicles were found to induce regulatory dendritic cells, resulting in reduced IFNγ secretion by interacting T cells and the appearance of FOXP3^+^ regulatory T cells [425]. Favaro et al. [633] further showed that MSC-derived extracellular vesicles were internalized by peripheral blood mononuclear cells isolated from T1D patients and prevented T lymphocyte activation following stimulation with the islet antigen glutamic acid decarboxylase. The vesicles also resulted in a shift in the cytokine profile with increased levels of TGFβ, IL-10, IL-6, and PGE_2_ [633]. Exosomes from adipose tissue-derived MSCs ameliorated autoimmune reaction in a streptozotocin-induced T1D mouse model with elevated levels of IL-4, IL-10, and TGFβ and concomitantly reduced levels of IL-17 and IFNγ [634]. Treating obese mice fed on a high-fat diet with MSC-derived extracellular vesicles resulted in increased glucose uptake and alleviation of insulin resistance [635].

MSC-derived extracellular vesicles have also been shown to have potential therapeutic applications in regenerative medicine [630,631,636,637,638,639,640] and, as such, they have been incorporated in several clinical trials [630]. The vesicles carry with them many of the bioactive molecules produced by MSCs and have the advantage of being cell-free, non-replicating, and showing low immunogenicity [630,631]. The small size of the vesicles allows them to be taken up by recipient cells through pinocytosis, resulting in alterations in their functionality and activities [631]. Among human diseases that have been treated with MSC-derived extracellular vesicles include acute respiratory distress syndrome (ARDS), wounds, and inflammatory diseases such as Crohn’s disease, ulcerative colitis, and periodontitis [630]. MSC-derived extracellular vesicles have further been shown to ameliorate diabetic foot ischemia and ulcer [641], diabetic nephropathy [642,643,644], and other diabetic-related complications [645].

**Table 5:** The secretome of MSCs.

Secreted Factor	Effects Associated with the MSC Secreted Factors *	References
Differentiation factors e.g., Activin A, BMP4, BMP6, TSP1	Activin A expression in MSCs is required for their chondrogenic and osteogenic differentiation. Activin A production by MSCs induces neuronal development and neurite outgrowth. Activin A and betacellulin, which are produced by MSCs, are involved in β-cell differentiation during development and β-cell regeneration in adults. Treating human islets with activin A led to reduced expression of genes associated with β-cell maturity (e.g., PDX1, MAFA, GLUT2), while increased genes expressed in immature β-cells (e.g., MAFB). Bone morphogenic protein 4 and 6 (BMP4 and BMP6) secreted by MSCs can promote differentiation of adipose tissue-derived MSCs. BMP4 has also been used to differentiate MSCs into keratinocytes. BMP2 and BMP4 stimulate the chemotactic migration of human mesenchymal progenitor cells. The roles of BMP2, BMP4, and BMP6 in β-cell differentiation and function are described in Table 1. Thrombospondin-1 (TSP1) is responsible for the activation of the latent secreted form of TGFβ1. Treating TSP1 knockout mice with a peptide derived from TSP1 led to TGFβ1 activation and reversal of the pancreatic abnormalities observed in the TSP1 knockout mice.	[230,258,375,581,584,594,646,647,648,649,650,651,652,653]
Chemokines, e.g., CXCL1, CCL2 (MCP1), CCL5 (RANTES), CCL7, CXCL4, CXCL5, CXCL12 (SDF-1), CXCL16; CCL22, eotaxin 2 (CCL24) and eotaxin 3 (CCL26), CCL28, Fractalkine (CX3CL1)	MSCs express a whole series of chemokines, among them CCL2, CXCL1, and CXCL5 are outstanding. CCL2, which recruits macrophages, is expressed at low levels in resting MSCs, but it is highly upregulated by inflammatory cytokines such as TNFα. CCL2 secreted from MSCs promotes the polarization of macrophages to an M2 neuroprotective phenotype. CCL2 and CXCL12 (SDF1) form heterodimers that induce IL-10 expression in CCR2-expressing macrophages, thus mitigating gut injury caused by dextran sulfate sodium (DSS)-induced colitis in mice. Stroma-derived factor-1 (SDF1/CXCL12) secreted from MSCs contributes to tissue regeneration and repair. SDF1 (CXCL12) regulates mobilization of neutrophils from the bone marrow. The MSC-derived SDF-1 increases the phagocytic function of neutrophils, resulting in better clearance of bacteria. Chemokines are also involved in the migration of MSCs to wounds and injured tissues.	[584,586,590,594,604,626,654,655,656,657]
Cytokines, e.g., IL-1α, IL-1β, IL-4, IL-6, IL-8, IL-10, GM-CSF, G-CSF, M-CSF	Human bone marrow-, human umbilical cord- and cord blood-derived MSCs express several cytokines, with the most prominent ones being IL-6 and IL-8. IL-4 is secreted by human islets, human Wharton’s Jelly MSCs, and mouse bone marrow-derived MSCs. IL-4 receptor is expressed on human islet cells. IL-4 protects β-cells from IFNγ/IL-1β-induced apoptosis by activating the PI3K and JAK/STAT pathways. IL-6 protects MIN6 β-cells against the pro-apoptotic signals delivered by IL-1β, IFNγ, and TNFα. Lipopolysaccharide (LPS) stimulates the secretion of GM-CSF, G-CSF, and M-CSF from adipose tissue-derived MSCs, which contributes to hematopoiesis. TNFα stimulation of human adipose tissue-derived MSCs resulted in the upregulation of IL-6, IL-8, and MCP-1, which stimulate the migration of monocytes.	[203,205,379,544,581,582,584,590,613,658]
Growth and survival factors, e.g., EGF, FGF6, FGF7, bFGF (FGF2), HGF, IGF2, PDGF-AA, PDGF-AB, PDGF-BB, VEGF, BDNF, GDF15, TSP1, adiponectin, TGFβ, SCF	Human and mouse MSCs secrete a whole battery of growth factors. GDF15 secreted from human umbilical cord blood-derived MSCs promotes neurogenesis and increases amyloid β-clearance by microglial cells. Thrombospondin-1 (TSP1) secreted by MSCs attenuates amyloid β peptide-induced synaptic dysfunction. PDGF-BB stimulates the chemotactic migration of human mesenchymal progenitor cells. Deletion of insulin-like growth factor 2 (IGF2) in pancreatic mesenchymal-derived cells results in acinar and β-cell hypoplasia. The effects of the various growth factors on β-cell function are described in Table 1.	[222,544,581,584,590,594,595,604,653,659,660,661]
IGFBPs	Insulin-like growth factor binding proteins (IGFBP) 1,2, 3, 4, and 6 are expressed in MSCs. IGFBPs are involved in regulating the effects of IGFs on growth, development, and metabolism by binding to IGF1 and IGF2. The anti-diabetic effect of leptin is in part mediated through induction of IGFBP2. Overexpression of IGFBP2 reversed diabetes in insulin-resistant *ob*/*ob* mice and streptozotocin-induced diabetic mice. IGFBP2 overexpression improved hepatic insulin sensitivity in *ob*/*ob* mice. IGFBP1 increased the number of cells in mouse and human islets that co-express glucagon and insulin. IGFBP1 promoted β-cell regeneration in Zebrafish. High IGFBP1 levels in humans reduced their risk of developing T2D.	[581,584,594,662,663]
Neurotrophic factors, e.g., BDNF, CNTF, βNGF, GDNF, NT4, NRG1	Human MSCs express several neurotrophic factors, including BDNF, CNTF, βNGF, Glial derived neurotrophic factor (GDNF), and neurotrophin 4 (NT4). Rat adipose tissue-derived MSCs express higher levels of neuroregulin 1 (NRG1) than rat bone marrow-derived MSCs. MSCs promote neurite outgrowth within dorsal root ganglion explants, which seems to be a combined effect of several secreted neuro-regulatory molecules since the βNGF levels in the MSC supernatant is far below the concentration required for inducing neurite outgrowths. Islets which are highly innervated express receptors for nerve growth factors. The effects of the neurotrophic factors on β-cell function are described in Table 1.	[270,584,594,595,601,603]
Factors involved in tissue regeneration, e.g., bFGF, EGF, GM-CSF, IGF, TSG6 and TSG14	MSCs promote wound closure in mouse wound models. MSCs promote cutaneous wound repair in diabetic mice. Through secretion of trophic factors such as bFGF, EGF, GM-CSF, and IGF, MSCs can promote the survival of stem and progenitor cells in its vicinity, resulting in improved tissue repair. TSG6 and TSG14 (Pentraxin-3/PTX-3) are involved in tissue repair and wound healing. TSG6 mediates the anti-fibrotic effects of MSCs on macrophages. TSG6 suppresses TNFα secretion from activated macrophages and induces a switch from a high fibrotic to a low anti-fibrotic TGFβ1/TGFβ3 ratio. TGFβ1 secreted by macrophages upon phagocytosis of neutrophils, terminates inflammation and induces myofibroblast differentiation. Together with enhanced release of TIMP1, TGFβ1 leads to wound and tissue fibrosis. TGFβ3, on the other hand, has anti-scarring activity, reduces early extracellular matrix deposition, and increases IL-10 production by macrophages. MSCs can further promote wound healing in mice by undergoing trans-differentiation into different skin cell types, including keratinocytes.	[581,584,602,652,664,665,666,667,668,669,670]
Pro-angiogenetic factors, e.g., VEGFA, VEGFB, VEGFC, VEGFD, Angiopoietin-1, Angiopoietin-2, Angiogenin, IGF-1, Netrin-1, HGF, IL-6, IL-8, MCP-1, CXCL16, PDGF, MMP8 and MMP9	MSCs promote vascular remodeling and angiogenesis by secreting a variety of pro-survival and angiogenic factors such as VEGF, Angiopoietin-1, Angiogenin, IGF-1, Netrin-1, HGF, IL-6, IL-8, MCP-1, CXCL16, PDGF, MMP8 and MMP9. TNFα activates MSCs to secrete the pro-angiogenic cytokines IL-6 and IL-8. The conditioned medium from TNFα-activated MSCs stimulated blood perfusion and angiogenesis when injected into the ischemic hindlimb of a mouse model. MMP9 knockout mice showed similar islet mass and distribution as wild-type mice but had an impaired glucose response in vivo with lower serum insulin levels. MMP9 knockout islets also showed reduced glucose-stimulated insulin secretion in vitro. The vascular density of the MMP9 knockout islets is reduced, with the capillaries having fewer fenestrations. Angiopoietin-1 (ANG1) and angiopoietin-2 (ANG2) stimulate islet-like development from human induced pluripotent stem cells (iPSCs). Netrin-1 produced by human Wharton’s jelly MSCs, belongs to a family of laminin-like proteins that interact with DCC/Neogenin-1 and UNC5 receptors to stimulate or inhibit angiogenesis, depending on the context. Islet grafts co-transfected with MSCs showed increased vascularization and were surrounded by von Willebrand factor-expressing endothelial cells.	[238,379,503,524,527,528,529,581,584,585,586,591,594,608,611,671,672,673,674]
Immunosuppressive factors, e.g., HGF, PGE_2_, IDO, TGFβ1, TGFβ3, GILZ, Activin A, IL-6, IL-10, nitric oxide, HO-1, TSG6, TSG14, VEGF, STC-1, PD-L1, MMP2 and MMP9	The secretion of HGF by MSCs suppresses T-lymphocyte proliferation. The secretion of TGFβ1 by MSCs suppresses T-lymphocyte proliferation. Secretion of indoleamine-2,3-dioxygenase (IDO) by MSCs, which is upregulated by IFNγ, inhibits the proliferation of activated T and NK cells by converting tryptophane into kynurenine. Human MSCs express higher levels of IDO than mouse MSCs, while mouse MSCs express higher levels of iNOS than human MSCs. The immunosuppressive function of MSCs is induced by IFNγ. TNFα and IFNγ act synergistically to induce PGE_2_ production in MSCs, which in turn suppresses immune cells. PGE_2_ secreted by MSCs polarizes macrophages to an M2 phenotype, producing the immunosuppressive cytokine IL-10. PGE_2_ secreted from MSCs reduces TNFα secretion while increasing IL-10 secretion from dendritic cells. Human umbilical cord-derived MSCs produce activin A and PGE_2_, which co-operate in suppressing IFNγ production by NK cells. IL-6 produced by MSCs increases the secretion of PGE_2_ from MSCs. Wild-type, but not IL-6-deficient MSCs, could alleviate the clinical signs of collagen-induced arthritis in mice. The MSCs modulate the host response by inducing a switch from a Th1/Th17 towards a Th2 immune profile. MSC-derived IL-6 inhibits the differentiation of dendritic cells as well as preventing the proliferation of T cells. MSC-derived IL-10 inhibits Th17 cell differentiation. Stimulation of MSCs with IL-10 induces the secretion of sHLA-G, which has immunosuppressive activities. Nitric oxide production by MSCs suppresses T cell proliferation. The expression of chemokines leads to the attraction of T cells to the MSCs where they become suppressed by nitric oxide produced by MSCs. Heme oxygenase -1 (HO-1) produced by MSCs co-operates with iNOS to induce immunosuppression. GILZ (glucocorticoid-induced leucine zipper or TSC22D3) produced by bone-marrow MSCs promotes regulatory T cells. When MSCs are primed with IFNγ and TNFα, GILZ translocates to the nucleus, where it stimulates the transcription of iNOS and activin A. Activin A, in turn, represses Th17 cells and enhances IL-10 production. TGFβ has immunosuppressive activities and is a major contributor to the immunosuppressive function of MSCs. TGFβ acts together with HGF to mediate the immunosuppressive function of MSCs. The exposure of MSCs to IL-4 and/or IL-13 increases their production of TGFβ. Activin A promotes the TGFβ-mediated conversion of CD4^+^CD25^-^ T cells into Foxp3^+^ regulatory T cells. VEGF has anti-inflammatory activities on activated T cells while increasing the number of regulatory T cells. 3D MSC cultures secrete tumor necrosis factor-stimulated gene 6 (TSG6), and stanniocalcin 1 (STC1). Tumor necrosis factor-α-induced gene/protein 6 (TSG6) promotes wound healing and contributes to an immunosuppressed environment, among others, by inducing M1 to M2 macrophage polarization. TSG6 interacts with CD44 receptor on macrophages to reduce zymosan/TLR2-mediated nuclear translocation of NFκB. TSG6 is also involved in reducing inflammation by MSCs under condition of myocardial infarction in an animal model. TSG6 mediates the anti-inflammatory effects of human MSCs in a mouse model of LPS-induced lung inflammation. TSG6 mediates the anti-inflammatory activity of human MSCs in a mouse model of severe acute pancreatitis. TSG14 (also called penetraxin 3, PTX3) binds to apoptotic cells and prevents their recognition by dendritic cells. NLRP3-activated macrophages stimulate human MSCs to secrete stanniocalcin 1 (STC1), which, in turn, inhibits NLRP3 inflammasome activation and ROS production in macrophages. Knockdown of stanniocalcin 1 (STC1) in MSCs prevents the suppression of chimeric antigen receptor (CAR)-T cells caused by MSCs. In addition, stanniocalcin 1 (STC-1) has anti-apoptotic activity. Secretion of PD-L1 and PD-L2 from MSCs, which is induced by IFNγ and TNFα, causes T cell immunosuppression. MMP2 and MMP9 secreted by MSCs cause immunosuppression by reducing CD25 surface expression on responding T cells. Inhibition of MMP2 and MMP9 prevented the MSC-mediated attenuation of delayed-type hypersensitivity responses to allogeneic antigens and restored T cell responses to IL-2 in a mouse model. Inhibition of MMP2 and MMP9 prevented the MSC-mediated protection of allogeneic islet grafts in a model of streptozotocin-induced diabetic mice.	[409,410,427,442,502,581,584,594,596,597,618,619,620,621,622,627,628,666,675,676,677,678,679,680,681,682,683,684,685,686,687,688,689,690,691,692,693,694,695,696,697,698,699,700,701,702,703,704,705,706,707]
Antioxidant factors, e.g., HO-1	Heme oxygenase-1 (HO-1) produced by MSCs exhibits antioxidant properties and protects islets from hyperglycemia-induced oxidative stress. HO-1 acts via the Nuclear factor erythroid 2-related factor 2 (NRF2) signaling pathway.	[495]
Other factors secreted by MSCs	GIF or migration inhibitory factor (MIF) protects from ischemic injury and cellular apoptosis. Leptin is strongly induced in MSCs upon hypoxia. Leptin controls appetite and energy balance and regulates secondary metabolism, cell survival, migration, and angiogenesis. Endostatin is an internal fragment of collagen XVIII that inhibits angiogenesis. Serpin E and Serpin F are serine proteinase inhibitors that control the activities of proteases involved in inflammation, complement, coagulation, and fibrinolytic pathways. Amphiregulin binds to EGF receptor and stimulates cell growth, survival, and migration. Amphiregulin promotes tissue repair. Amphiregulin reduces ER stress in cultured islets. Amphiregulin is upregulated in islet-infiltrated regulatory T cells. Osteostatin M inhibits osteoclast activity and differentiation. RANKL (receptor activator of NFκB ligand) negatively regulates osteoblastic bone formation. TIMP1, TIMP2, and TIMP4 inhibit MMPs and thus regulate tissue repair.	[524,525,527,581,584,590,594,708,709,710]

* β-cell related functions are described in Table 1.

Dietrich et al. [590] studied the cytokine content in supernatants of mono- or co-culture of human islets and Wharton’s jelly MSCs. This study showed a higher expression of IL-1β, IL-17, IFNγ, IL-4, IL-10, IL-13, Granulocyte-macrophage colony-stimulating factor (GM-CSF), and leptin in the supernatant of the co-cultures than in islet or MSC monocultures [590]. They also observed that human islets secrete various chemokines, including CCL2, CCL3, CCL4, and GROα (CXCL1), and to a lesser extent, CCL5. Wharton’s jelly MSC monocultures secreted much higher levels of CCL4 than islets, while lower levels of CCL2 and GROα (CXCL1) than the islets [590]. Both the human islets and human MSCs produce adiponectin, which was not further upregulated in the co-cultures [590]. 

To better understand the protective activity of MSCs on islet function, it was intuitive to look for similarities and differences in the growth factor profile of human MSCs and human islets using a human growth factor RT Profiler PCR Array. This study showed that there are growth factor genes that are expressed in both MSCs and islets, while others are more prominent in one cell type in comparison to the other (Figure 1 and Figure 2). Among the genes expressed in both human MSCs and human islets, we could find *BMP1*, *CSF1*, *FGF2*, *FGF14*, *IGF2*, *INHBA*, *MDK*, *PDGFC*, *PGF*, *SPP1*, *TGFB1*, *VEGFA*, and *VEGFC* (Figure 1 and Figure 2A). Genes that are highly expressed in islets, with relatively low levels in MSCs include *BMP5*, *BMP8b*, *CECR1*, *CXCL1*, *FGF13*, and *LEFTY1* (Figure 1 and Figure 2B). The human islets also express the cytokines IL-11, IL-18, IL-1α, and IL-1β (Figure 1 and Figure 2B). On the other hand, genes predominantly expressed in human MSCs with relatively low expression of human islets include *BDNF*, *DKK*, *FGF5*, *FGF7*, *IGF1*, JAG1, *NGF*, NRG1, *PTN*, *LTBP4*, and *NDP* (Figure 1 and Figure 2C).

BMP1 is a procollagen C-proteinase that has been shown to promote osteogenesis of bone marrow-derived MSCs [711]. BMP1-like proteases are also involved in the activation of growth factors by cleaving BMP2, BMP4, GDF11, and TGFβ1 [712]. In addition, it cleaves both human and mouse IGFBP3, thereby reducing the ability of IGFBP3 to bind and block IGF1 [713]. The bone morphogenic protein BMP5 has previously been shown to be exclusively expressed in β-cells among islet cells [263,264,265]. BMP5 has been implicated in the development of fetal pancreatic epithelium [266].

The growth factor array showed that MSCs express several FGF genes including FGF2, FGF5, FGF7, and FGF14. Among them, FGF2 and FGF7 have been used in β-cell differentiation protocols (e.g., [235]). FGF2 (basic FGF) is a notochord factor that represses endodermal sonic hedgehog, thereby permitting the expression of the pancreatic genes PDX1 and INS (insulin) [300]. FGF7, also known as keratinocyte growth factor (KGF), has been shown to lead to ductal cell differentiation into β-cells [299], and it is included in the differentiation protocol of human pluripotent stem cells into β-cells [235]. FGF10 has been previously described as a mesenchymal factor that promotes the development of pancreatic epithelium [301]. FGF14 has also previously been shown to be produced by MSCs [714] and mouse islets [715] and might play a role in fine-tuning neuronal function [716]. 

VEGF expression is important for the highly developed vascularization in islets, which is crucial for the rapid endocrine responses to variances in glucose blood levels [378]. VEGF also acts as a survival factor for human islets [377]. VEGF has repeatedly been shown by other research groups to be expressed in both MSCs [581,585,594,599,605,612] and islets [72,717,718,719]. CSF1 (M-CSF), which supports the differentiation and survival of monocytes and macrophages, can induce the polarization of macrophages to a pro-angiogenic M2 phenotype [720,721].

Two INHBA (inhibin βA) subunits form the homodimeric activin A, which is a differentiation factor affecting β-cell differentiation, β-cell regeneration, and glucose-stimulated insulin secretion [227,228,229,230,722] (Table 1). In some studies, activin A and TGFβ1 have been included in the early steps of β-cell differentiation in vitro. However, at later differentiation stages, inhibition of the TGFβ/activin/nodal and BMP pathways was required for induction of *PDX1* and *INS* gene expression [226]. Follistatin, which is also expressed in islets [723], inhibits the activin A-mediated down-regulation of *PDX1*, *MAFA*, and *GLUT2* in a mouse β-cell line [648].

MSCs were found to produce several neurotrophic factors, including Midkine, BDNF, NGF, NRG1, and PTN (pleiotrophin). Taking into account the similarities between neuron and β-cell evolution despite being derived from different germ layers [724], it is likely that the MSC-secreted neurotrophic factors might have a beneficial role in insulin-producing β-cells. Midkine (neurite growth-promoting factor 2, MDK) is a heparin-binding cytokine that promotes the growth, survival, and migration of target cells such as neural precursor cells [725]. Pleiotrophin (PTN) is another heparin-binding cytokine with neurotrophic activities [725]. In mice at the mRNA level, PTN is especially expressed in immature β-cells with low GLUT2 expression [726]. The PTN peptide was detected in adult mouse islets with a predominant presence in β-cells [726]. In the embryonic pancreas, PTN appears in areas of blood vessel formation near differentiating ductal epithelium [727]. Inhibition of PTN expression in mouse embryonic pancreatic primordia explants prevented full maturation of endocrine precursors with impaired insulin and glucagon expression [727]. Further studies suggest a role for PTN in β-cell proliferation and regulation of glucose homeostasis [728,729]. BDNF has been shown to bind to the TrkB.T1 receptor on β-cells, resulting in increased GSIS [247]. There are also some lines of evidence that NGF may fine-tune insulin secretion through acting on the TrkA receptor expressed in β-cells [224,225]. β-cells might themselves transiently secrete NGF upon glucose stimulation [224,225].

Many differentiation protocols of embryonic stem cells (ESCs) and induced pluripotent stem cells (iPSCs) have included various growth factors such as GDF8, FGF7, FGF10, activin A, FGF2, BMP4, HGF, IGF1, Wnt3a, and FGF7 (KGF), in attempts to obtain insulin-producing β-cells [233,234,235,730,731,732,733,734,735,736,737,738,739,740,741]. The choice of growth factors has, in general, been based on the knowledge of growth factors required for normal pancreatic development [730,742,743]. Most of these studies have the limitation of low percentage of mature β-cells and short-term maintenance. Our observation that MSCs can sustain islet function in vitro suggests that MSCs ought to be included in the β-cell differentiation protocols.

## 6. Conclusions

**Figure 3:**
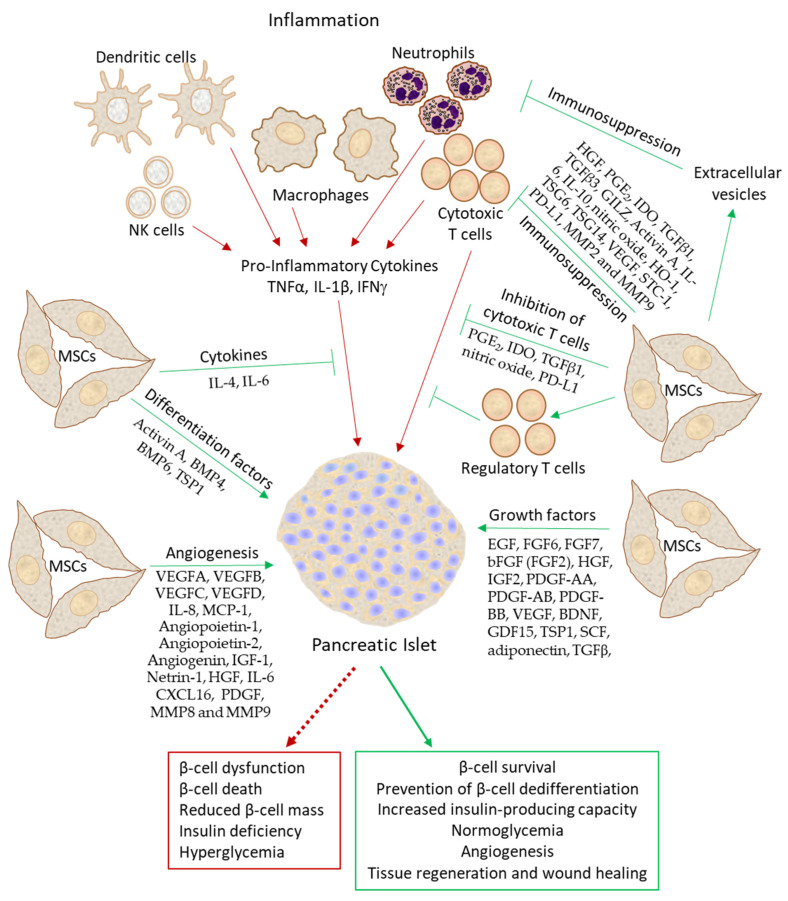
An illustration of the beneficial effects of MSCs on pancreatic β-cells. The red arrows show the deleterious effects of inflammation on β-cells, whereas the green arrows show the beneficial effects of MSCs on β-cells, including the prevention of the harmful effects of cytotoxic T cells and inflammatory cytokines.

In this review, we have addressed various aspects of immune-mediated β-cell destruction and cytokine-induced β-cell death leading to T1D diabetes, as well as the β-cell protective roles of MSCs and their potential clinical applications in regulating glucose homeostasis with reduced insulin requirement and even insulin independence in selected early onset diabetic people. Another important topic discussed is the MSC secretome of diverse cytokines, chemokines, growth factors, angiopoietic factors, and immunosuppressive factors, which collectively influence various aspects of diabetes pathogenesis, ultimately providing a microenvironment that promotes β-cell differentiation, growth, and survival, and protects the β-cells from the hazardous effects of immune cells and pro-inflammatory cytokines. The protective effects of MSCs on β-cells are a combination of β-cell differentiation, maintenance of mature β-cell functions, β-cell growth and survival, regulation of insulin secretion including glucose-induced insulin secretion, angiogenesis, protection against hypoxia-induced and cytokine-induced β-cell damage, and immunosuppression (Figure 3). An additional component is the ability of MSCs by themselves to differentiate into insulin-producing cells when encountering islets and their attraction to injured and inflamed areas. The human studies have so far focused on the transplantation of bone marrow monocytes and MSCs with or without concomitant immunosuppressive therapy, usually resulting in a transient beneficial effect with some diabetic people reaching long-term effects. Considering the data obtained from animal studies, it would be desirable to combine the MSCs with islet transplantation. However, the sparse amount of human islets available for this purpose is a limitation. The increased knowledge of the MSC secretome can be used in further studies to optimize the growth factor composition for preserving β-cell function and to increase the efficiency of in vitro β-cell differentiation. Another recommendation would be to use MSCs to preserve the ex vivo function of islets.

## Figures and Tables

**Table 1:** Growth factors supporting β-cell survival and function.

Growth Factor	Effect on β-Cell Function	References
Activin A	Activin A could be detected by immunostaining at an early embryonic stage of rat pancreatic development. Activin A is expressed in fetal rat pancreas and human β-cells. Activin A, which belongs to the transforming growth factor-β (TGF-β) family, stimulates insulin secretion in cultured human and rat pancreatic islets when incubated in the presence of glucose. Activin A phosphorylates SMAD2 and SMAD3. Activin A, in combination with betacellulin, lowered the serum glucose concentration of newborn Sprague-Dawley rats that have been made diabetic with streptozotocin. The number of islets was increased by the combined treatment of activin A and betacellulin. Activin A and TGFβ1 have been included in the early steps of β-cell differentiation of stem cells in vitro. However, at later differentiation stages, inhibition of the TGFβ/activin/nodal and BMP pathways was required for induction of *PDX1* and *INS* gene expression. The activin B receptor ALK7 was found to be a negative regulator of pancreatic β-cells. When TGFβ signaling was disrupted in adult mouse β-cells by conditional overexpression of SMAD7 in PDX1^+^ cells, the mice developed diabetes, which could be reversed upon resumption of islet TGFβ signaling. However, when SMAD7 was activated in PDX1^+^ mouse β-cells during embryonic development, β-cell hypoplasia and neonatal lethality occurred.	[226,227,228,229,230,231,232,233,234,235,236,237]
ANG1 and ANG2	Angiopoietin 1 (ANG1) and angiopoietin 2 (ANG2) promote the generation of all hormone-producing cells of the islets (including insulin, glucagon, somatostatin, and pancreatic polypeptide) from human induced pluripotent stem cells (iPSCs). Both systemic ANG1 knockout mice and β-cell-specific ANG1 knockout mice showed reduced serum insulin levels and glucose intolerance, suggesting an essential role of angiopoietin-1 for normal islet function.	[76,238]
BDNF	Brain-derived neurotrophic factor (BDNF) reduces glucagon secretion while increasing glucose-induced insulin secretion from mouse islets. BDNF receptor TrkB.T1 is expressed on β-cells. TrkB.T1 knockout mice showed impaired glucose tolerance and insulin secretion. BDNF is secreted from differentiated human muscle cells and thus might be a mediator to increase glucose metabolism during exercise. Repeated administration of BDNF to obese diabetic *db*/*db* mice reduces blood glucose concentration. Repeated BDNF treatment of *db*/*db* mice increases the islet insulin content. BDNF increases the hepatic glucokinase levels and improves the response to insulin in obese insulin-resistant rats. BDNF suppresses hepatic glucose production. BDNF reduces food intake.	[239,240,241,242,243,244,245,246,247]
BMP2, BMP4, BMP5 and BMP6	Both Bone morphogenetic protein 2 (BMP2) and BMP4 are expressed in islets. BMP4 is expressed in islet ε-cells, with very low levels in α, β, γ, or δ-cells. During mouse pancreatic development, BMP4, BMP6, BMP7, and TGFβ1 were detected in E13.5, E15.5, and E17.5 fetal mouse pancreas, while BMP2, BMP5 and activin A were detected only at the later E17.5 stage. BMP4, BMP5, and BMP6 promoted the formation of cystic colonies from dissociated E15.5 mouse pancreatic cells, which required the presence of laminin 1. The activity of these BMPs was antagonized by activin A and TGFβ1. BMP2 is upregulated by pro-inflammatory cytokines in rodent and human islets. BMP2 and BMP4 are upregulated in animal models of T1D and T2D. BMP5 is exclusively expressed in β-cells. Both BMP2 and BMP4 play important roles during β-cell development. The BMP signaling acts within a timely temporal window during embryonic pancreatic development to either promote or prevent β-cell differentiation. In a Zebrafish model, BMP signaling is required for the formation of ventral pancreatic cells, but when exposed to BMP signaling, they do not develop into β-cells. Inhibition of BMP signaling at the late embryonic stage by using the BMP receptor inhibitor dorsomorphin resulted in increased β-cell neogenesis near the extrapancreatic duct. Thus, BMP signaling is required in specific time windows during β-cell differentiation. BMP4 induces the expression of Inhibitor of DNA binding (Id) proteins that bind to the basic helix-loop-helix (bHLH) transcription factor NeuroD, required for embryonic differentiation of islets. Thus, BMP4 blocks the differentiation of endocrine progenitor cells while promoting their expansion. The WNT ligand WNT5A improves β-cell differentiation, among others, by cooperating with Gremlin 1 to inhibit the BMP pathway during β-cell maturation. BMP2 and BMP4 inhibit basal and growth factor-stimulated proliferation of primary β-cells from adult rats and mice. Glucose-induced insulin secretion was impaired in adult rodents and human islets pre-treated with BMP4. The islet pericytes are important for β-cell function, among others, through production of BMP4. Transcription factor 12 (TCF12) of the canonical Wnt signaling is required for BMP4 secretion from pancreatic pericytes. Inactivation of pericytic TCF12 in mice results in impaired glucose tolerance, comprising β-cell function and glucose-stimulated insulin secretion. Exogenously added BMP4 rescues the impaired glucose-induced insulin secretion. In a transgenic mouse model, BMP4 promotes the expression of core β cell genes and enhances glucose-stimulated insulin secretion and glucose clearance. The BMP4 receptor BMPR1A (BMP type 1a receptor or ALK3) is expressed on β-cells, and mice with attenuated BMPR1A signaling in β-cells develop diabetes due to impaired insulin secretion. The β-cells from these mice express lower levels of genes involved in insulin gene expression and show impaired glucose-stimulated insulin secretion. High glucose increases the expression of BMP2 and BMP4 in human aortic endothelial cells, resulting in vascular inflammation. Noggin, a bone morphogenic protein inhibitor, which is often co-upregulated with BMPs, reduces serum glucose levels in *db*/*db* T2D mice. BMP4 is one of the growth factors used to differentiate human embryonic stem cells and human induced pluripotent stem cells into definitive endoderm cells. Treatment of leptin-deficient *ob*/*ob* mice (T2D model) with BMP6 led to reduced blood glucose and lipid levels while increasing plasma insulin levels.	[134,146,248,249,250,251,252,253,254,255,256,257,258,259,260,261,262,263,264,265,266]
CCK	Cholecystokinin (CCK) is a gut hormone that is also expressed in islets. CCK is upregulated in obesity. CCK production and secretion is induced in β-cells by GLP-1. Loss of CCK results in increased β-cell apoptosis in obese mice. CCK promotes β-cell survival in rodent models of diabetes. CCK reduces cytokine-mediated apoptosis of β-cells and human and mouse islets.	[267,268,269]
CNTF	Ciliary neurotrophic factor (CNTF) reduces glucose-induced insulin secretion of rat islets. CNTF promotes islet cell survival by upregulating Connexin 36, PAX4, and Bcl-2. CNTF, together with EGF, protects mice from alloxan-induced hyperglycemia by increasing the number of β-cells.	[270,271]
CTGF	Connective tissue growth factor (CTGF) is highly expressed in islet vasculature and interacts with TGFβ and Wnt signaling pathways. CTGF contributes to β-cell proliferation and development in mice. CTGF is expressed in endothelial cells, pancreatic ducts, and embryonic β cells. Inactivation of endothelial CTGF leads to decreased islet vascularity and decreased embryonic β-cell proliferation. Overexpression of CTGF in β-cells promotes proliferation of immature embryonic β-cells.	[272,273,274,275]
EGFs	Exposure of human islets to epidermal growth factor (EGF) leads to dedifferentiation into duct-like epithelial structures. Dedifferentiation is important for the EGF-induced proliferation, for later to undergo redifferentiation. EGF receptor (EGFR) signaling is essential for proper fetal development of islets and for sufficient β-cell mass post-partum. EGFR signaling is required for β-cell mass expansion during high-fat diet and during pregnancy in mice. Combined treatment of human islets with EGF and gastrin in vitro increased the number of β-cells as well as the insulin content of the cultured islets. EGF as well as TGFα transiently induced phosphorylation of ERK1/2, GSK3 and AKT (PKB) in the INS-1 rat β-cell line. EGF and gastrin restored normoglycemia and induced islet regeneration in alloxan-induced diabetic mice. Treatment of alloxan-induced diabetic mice with EGF and ciliary neurotrophic factor (CNTF) led to an increased number of insulin-positive cells and normalization of blood glucose levels. Treatment of rat exocrine pancreatic cells with EGF and leukemia inhibitory factor (LIF) resulted in transdifferentiation into insulin-producing cells expressing C-peptide, PDX1, and GLUT2. Transgenic mice overexpressing EGF and keratinocyte growth factor (KGF) under the insulin promoter showed profound alterations in pancreatic morphology with an enlargement of the islets, profound intra-islet fibrosis, and appearance of intra-islet duct cells. Heparin-binding epidermal growth factor (EGF)-like growth factor (HB-EGF) stimulates β-cell proliferation of rat and human islets. Heparin-binding epidermal growth factor (EGF)-like growth factor (HB-EGF) mRNA levels are increased in β-cells in response to glucose. Epiregulin, a member of the EGF-related growth factor family, stimulates proliferation of rat β-cell lines through activation of EGFR/ErbB1. Epiregulin increases glucose uptake in *Lep^ob^* mice by binding to leptin receptors, resulting in translocation of the glucose transporter GLUT4 to the cell surface. Betacellulin, a member of the epidermal growth factor family, is produced by proliferating pancreatic β-cells and induces the differentiation of a pancreatic acinar cell line into insulin-secreting cells, an effect enhanced by activin A. In a dorsal embryonic pancreas culture from day 11.5 embryos, betacellulin enhances branching morphogenesis and increases PDX1 and insulin production while it inhibits the production of amylase and glucagon. Betacellulin increases the number of β-cells in islets of streptozotocin-treated mice. Betacellulin ameliorates hyperglycemia in obese leptin receptor-deficient *db*/*db* mice (T2D model).	[226,271,276,277,278,279,280,281,282,283,284,285,286,287,288,289,290,291,292,293,294,295,296]
FGFs	Fibroblast growth factors (FGFs) are required for β-cell differentiation during pancreatic development. FGF receptor (FGFR) 1 and 2, and the ligands FGF1, FGF2 (basic FGF, bFGF), FGF4, FGF5, FGF7 (keratinocyte growth factor, KGF), and FGF10 are expressed in adult mouse β-cells. FGF7/KGF leads to ductal cell differentiation into β-cells. FGF7/KGF promotes β-cell regeneration by stimulating duct cell proliferation. Overexpression of FGF7/KGF in acinar tissue leads to islet hyperplasia. Mice overexpressing FGF7/KGF under the proximal elastase promoter showed an increased number of islets and increased islet size at the average. Overexpression of FGF7/KGF in β-cells under the insulin promoter caused the islets to become fibrotic. FGF7/KGF treatment of mice that have been transplanted with human fetal pancreatic cells led to an increased number of β-cells in the graft. FGF7/KGF serum level increases with age. FGF10 promotes the development of pancreatic epithelium. FGF21 increased insulin levels in normal rat islets but did not potentiate glucose-induced insulin secretion. However, in islets from diabetic rats, FGF21 increased both islet insulin content and glucose-induced insulin secretion. FGFR1c signaling is required for the expression of pro-insulin convertases. Mice with defective FGFR1c signaling developed diabetes with age and showed a lower β-cell mass. FGF2 and FGF7/KGF have been used in β-cell differentiation protocols. FGF2 (basic FGF) is a notochord factor that represses endodermal sonic hedgehog (SHH), thereby permitting the expression of the pancreatic genes PDX1 and INS (insulin).	[164,235,297,298,299,300,301,302,303]
GDF11	Growth differentiation factor 11 (GDF11) is expressed in embryonic islet progenitor cells that express neurogenin 3 (NGN3), and promotes β-cell differentiation during pancreas development. GDF11-deficient mice have an increased number of NGN^+^ islet progenitor cells, reduced β-cell numbers, and impaired β-cell maturation.	[304,305]
GDF15(MIC-1)	Growth/differentiation factor 15 (GDF15/MIC-1) expression is suppressed under inflammatory conditions and in patients with T1D diabetes. Obese people have increased plasma GDF15 concentrations, with the highest concentrations observed in T2D patients. T2D individuals show increased GDF15 (MIC-1) serum levels. IL-1β and IFNγ suppress GDF15 mRNA in the islets. Exogenously added GDF15 protects the islets from IL-1β and IFNγ-induced apoptosis. GDF15 prevents insulitis and decreases incidence of diabetes in NOD mice. GDF15 improves insulin sensitivity in mice fed with a high-fat diet. GDF15 decreases body weight and increases insulin sensitivity in *ob*/*ob* mice, which is attributed to elevated oxidative metabolism and lipid mobilization in liver, muscle, and adipose tissue. GDF15 is an anti-inflammatory cytokine that decreases TNFα production in lipopolysaccharide-activated macrophages. Especially high expression of GDF15 is found in the acinar and ductal cells of the exocrine pancreas. GDF15 is upregulated by the ER stress CHOP transcription factor and the Th2 cytokines IL-4 and IL-13. GDF15 is required for the IL-13-induced improvement of glucose intolerance in mice fed with a high-fat diet.	[39,252,306,307,308,309,310,311,312,313]
GH	Growth hormone (GH) stimulates proliferation and insulin production of β-cells through a mechanism involving the activation of Janus kinase 2 (JAK2)/ Signal transducer and activator of transcription 5 (STAT5) signaling pathway. GH activation of STAT5 protects β-cells from cytokine (IFNγ, TNFα, IL-1β)-induced apoptosis. Growth hormone and IGF-1 synergistically increase the survival and proliferation of β-cells.	[210,314,315,316,317]
GIP	Gastric inhibitory polypeptide (GIP; also termed glucose-dependent insulinotropic polypeptide) is an incretin that is released by enteroendocrine K-cells after meal ingestion and promotes survival and proliferation of β-cells, as well as potentiating insulin secretion. Activation of the GIP receptor leads to stimulation of adenylyl cyclase and Ca^2+^-independent phospholipase A2 and activation of PKA and PKB. GIP increases the expression of the anti-apoptotic Bcl-2 and decreases the expression of the pro-apoptotic Bax. T2D patients do not respond to the insulinotropic activity of GIP.	[318,319,320,321,322,323,324,325,326]
GLP-1	Glucagon-like polypeptide-1 (GLP-1) is an insulinotropic intestinal-derived incretin that is released after meal ingestion and promotes survival and proliferation of rodent β-cells. GLP-1 enhances insulin secretion and reduces glucagon secretion. GLP-1 increases cholecystokinin (CCK) production in β-cells, which protects them from cytokine-induced apoptosis. Exendin-4 is a GLP-1 analog resistant to cleavage by dipeptidyl peptidase 4 (DPP-IV) and acts as a GLP-1 receptor agonist to increase cAMP intracellular levels and improve glycemic control in T2D patients. GLP-1 and exendin-4 stimulate β-cell neogenesis in streptozotocin-treated newborn rats. Exendin-4 promotes the differentiation and maturation of human fetal pancreatic cells. Exendin-4 protects against cytokine-induced β-cell death by increasing connexin 36 gap junction levels on the plasma membrane.	[269,318,319,320,321,325,327,328,329,330,331,332,333,334,335]
HGF	Hepatocyte growth factor (HGF) promotes fetal β-cell proliferation and proliferation of mature β-cells in pregnancy. Fetal pancreas-derived fibroblasts express HGF, which stimulates β-cell proliferation and islet cluster formation. HGF, together with activin A, induces the differentiation of a pancreatic acinar cell line into insulin-secreting cells. HGF increases islet engraftment and islet transplant performance in diabetic rodents. The beneficial effects of HGF seem to be both due to the protection of β-cells from cell death and promotion of their proliferation. The HGF-induced proliferation of β-cells was increased by low glucose concentrations (3–6 mM). HGF activates the JAK2/STAT5 and phosphatidylinositol-3’-kinase (PI3K)/AKT pathways. Cultivation of human islets in fibrin gel together with HGF preserved β-cell function and increased engraftment in a mouse model. Overexpression of HGF in the β-cell of adult transgenic mice results in increased β-cell mass, β-cell proliferation, and β-cell survival. These mice were more resistant to the diabetogenic effects of streptozotocin. Injection of an HGF-expressing plasmid prior to streptozotocin treatment of mice attenuated diabetes development with a slower increase in blood glucose levels and maintenance of higher serum insulin levels. These effects seem to be due to protection of the islets from the β-cytotoxic effects of streptozotocin. The HGF treatment increased pro-survival Akt kinase activation and Bcl-xL expression in the islets of the mice. Disruption of the HGF/c-Met signaling in mice results in increased β-cell death and early onset of diabetes in a mice model of multiple low-dose streptozotocin administration.	[336,337,338,339,340,341,342,343,344,345,346,347,348,349]
IGF1 and IGF2	Insulin-like growth factor (IGF1) is important for maintaining normal glucose homeostasis. IGF1 promotes β-cell survival and proliferation and decreases β-cell apoptosis. Treatment of rat islets with IGF1 prevented IL-1β-induced nitric oxide production and the consequent reduction in islet cell death. IGF1 activates ERK1/2, GSK3 and the PI3K/AKT signaling pathway in INS-1 rat β-cells. Mice with specific deficiency of the IGF1 receptor in β-cells showed normal β-cell mass but had reduced expression of GLUT2 and glucokinase, resulting in defective glucose-stimulated insulin secretion and impaired glucose tolerance. IGF2 is a survival signal for β-cells. IGF2 is expressed in fetal and neonatal rat islet cells but declines rapidly 2 weeks after birth, which is associated with increased neonatal islet cell death. Transgenic mice overexpressing IGF2 led to increased islet mass and prevented the apoptosis of islet cells seen in normal mice between postnatal days 11 and 16. IGF2 prevents cytokine (IFNγ, IL-1β, TNFα)-induced islet cell death.	[215,216,217,296,350,351,352]
INGAP	Islet neogenesis-associated protein (INGAP) protects β-cells from cytokine-induced apoptosis. INGAP, which is expressed in pancreatic acinar cells, can stimulate islet production from pancreatic progenitor cells. Transgenic mice overexpressing INGAP in pancreatic acinar cells are resistant to hyperglycemia induced by streptozotocin.	[212,353,354,355]
NGF	Nerve growth factor (NGF) is expressed in the pancreatic vasculature, and its TrkA receptor is found on β-cells. High glucose concentration increases NGF secretion and activates the TrkA receptor, resulting in increased insulin secretion both in mouse and human islets. NGF augments glucose-induced insulin secretion. Tissue-specific deletion of NGF or TrkA receptor in mice impaired glucose tolerance and insulin secretion. It is proposed that NRG fine-tunes insulin secretion by maintaining low basal insulin secretion while increasing glucose-stimulated insulin secretion.	[224,225]
NRGs	Neuroregulin (NRG)1α is expressed in β-cells, while NRG1β and NRG3 are mainly found in α-cells. NRG3 increased the proliferation of a rat insulinoma cell line. NRG4 increased the insulin secretion from a rat insulinoma cell line. NRG4 is an adipokine that increases glucose metabolism in peripheral organs.	[356,357]
OPN	Osteopontin (OPN) protects islets and β-cells from IL-1β-mediated cytotoxicity and streptozotozin-induced β-cell death by negatively regulating nitric oxide production. Osteopontin inhibits the synthesis of inducible nitric oxide synthase (iNOS). IL-1β and IFNγ treatment of human islets led to downregulation of osteopontin (SPP1), while streptozotocin treatment of mice led to an upregulation of osteopontin. The increased osteopontin expression may be a feedback mechanism to counterbalance the effects of pro-inflammatory cytokines. Osteopontin improved glucose-stimulated insulin secretion in mildly diabetic rat islets and in human islets from cadaver diabetic donors but not of islets from cadaver normoglycemic donors. Osteopontin serum level increases with age. High glucose and incretins stimulate islet osteopontin secretion.	[39,164,219,326,358,359]
PDGF-AA	Platelet-derived growth factor AA (PDGF-AA) isoform promotes β-cell proliferation in human juvenile β-cells that express PDGF receptor, while it does not affect adult human β-cells that do not express the PDGF receptor. PDGF-AA promotes proliferation of β-cells and improves their insulin-secretion function. The serum and tissue level of PDGF-AA, which is produced by osteoblasts, decreases with age.	[360,361]
PIGF	Placental growth factor (PIGF) is expressed in β-cells of adult mouse pancreas. PIGF levels are increased in β-cells during pregnancy. Knocking down PIGF in β-cells resulted in reduced β-cell proliferation and impaired glucose tolerance in pregnant mice.	[362]
PL-I	Placental lactogen I (PL-I) promotes β-cell proliferation, increases the β-cell mass, and results in hypoglycemia in a transgenic model overexpression of this gene.	[363]
Prolactin	Prolactin stimulates proliferation and insulin production of β-cells. Prolactin activates the JAK2/STAT5 pathway and phosphatidylinositol-3’-kinase (PI3K). Prolactin upregulates the expression of Survivin, which promotes β-cell proliferation.	[314,315,364,365]
PTHrP	Parathyroid hormone-related protein (PTHrP) induces insulin expression by activating MAP kinase-specific phosphatase-1 that dephosphorylates JNK. PTHrP is present in the pancreatic islet. An N-terminal PTHrP peptide (1–36) stimulates β-cell proliferation and preserves β-cell function in adult mice.	[366,367,368,369,370]
SDF-1/CXCL12	Stromal cell-derived factor 1 (SDF-1; also known as C-X-C motif chemokine 12 (CXCL12)) promotes β-cell development and survival. It is expressed in developing β-cells and during adult β-cell regeneration but repressed in terminally differentiated mature β-cells. SDF-1 enhances glucose-stimulated insulin secretion by human pluripotent stem cells and induces β-cell specific genes in these stem cells. SDF-1 protects islets from cytokine-induced apoptosis. SDF-1 causes immunosuppression.	[220,371,372]
TSP1	Thrombospondin 1 (TSP1) protects β-cells from lipotoxicity by activating the PKR-like ER kinase (PERK)—nuclear factor erythroid-2-related factor-2 (NRF2) signaling pathway involved in producing a protective antioxidant defense response. The anti-apoptotic growth factor thrombospondin is downregulated by IL-1β and IFNγ in human islets. TSP1-deficient mice are glucose intolerant despite having an increased β-cell mass. Their islets showed decreased glucose-stimulated insulin release, insulin biosynthesis, and glucose oxidation rate. One of the positive effects of TSP1 on pancreatic islet morphology is mediated by the activation of TGFβ1. TSP1 is mainly expressed in the endothelium of the normal islet. TSP1-deficient mice are glucose intolerant despite showing increased β-cell mass.	[39,373,374,375]
VEGF	Microvasculature plays an important role for intact islet function. Human islets produce several VEGF isoforms and express VEGF receptors 1, 2, and 3 as well as the co-receptor Neuropilin 1. Vascular endothelial growth factor (VEGF) affects both vascularization and innervation of the pancreatic islets. VEGF production by pancreatic islets is essential for islet vascularization and function. Mice with reduced VEGF-A expression in β-cells showed impaired glucose-stimulated insulin secretion. The highly developed vascularization in islets is essential for the endocrine responses to variances in glucose homeostasis. VEGF also acts as a survival factor for human islets. VEGF prevents human islet death induced by serum starvation. Transplantation of VEGF-overexpressing mouse islets into streptozotocin-induced syngeneic diabetic mice improved glycemic control already at day 1 post-transplantation, suggesting increased survival of the islet graft. The immunosuppressive drug rapamycin reduces islet VEGF secretion as well as islet viability and insulin release. Transplantation of rat islets together with vascular endothelial cells carrying a VEGF-expressing plasmid into diabetic rats restored blood glucose and insulin levels more efficiently than islets alone.	[72,376,377,378,379,380]

**Table 4:** Evidence for beneficial effects of hematopoietic stem cells and MSCs in diabetic patients.

Effects of Stem Cell Treatment in Diabetic Patients	References
In newly diagnosed T1D patients who received high-dose immunosuppression followed by autologous nonmyeloablative hematopoietic stem cell transplantation (AHST), 14 out of 15 patients became insulin-free for a period of 6–35 months. Patients with diabetic ketoacidosis failed to benefit from autologous nonmyeloablative hematopoietic stem cell transplantation. Adverse effects were observed in one patient who developed acute culture-negative bilateral pneumonia, and 2 patients appeared with late endocrine dysfunction (hypothyroidism or hypogonadism).	[558]
Intraportal administration of human adipose-tissue-derived, insulin-producing MSCs together with unfractionated cultured bone marrow to five insulinopenic T1D patients resulted in a 30–50% reduction in insulin requirement with a 4–26-fold increase in serum C-peptide levels, as analyzed after 1.5 to 3.5 months after treatment.	[485]
Autologous nonmyeloablative hematopoietic stem cell transplantation (HSCT; prepared by cyclophosphamide and granulocyte colony-stimulating factor (G-CSF)-mediated mobilization into the blood) to 23 newly diagnosed T1D patients without prior ketoacidosis resulted in insulin-free condition in 20 of the individuals. 12 patients remained insulin-free for 14–52 months, and of these, 8 patients relapsed and required low insulin doses. Patients who benefited from HSCT experienced an increase in C-peptide levels. Two patients developed bilateral nosocomial pneumonia, 3 patients developed late endocrine dysfunction, and 9 patients developed oligospermia.	[559]
A case report of a man with early-onset T1D who received 4 doses of cyclophosphamide along with anti-thymocyte globulin followed by autologous hematopoietic stem cell transplantation. This treatment led to normoglycemia that lasted more than 5 months after transplantation.	[560]
Co-transplantation of human adipose tissue-derived insulin-secreting mesenchymal stem cells and cultured human bone marrow into 11 male T1D patients resulted in reduced exogenous insulin requirement, increased serum C-peptide levels, and no diabetic ketoacidosis. The differentiation of adipose tissue-derived MSCs into insulin-producing cells was done by exposure the cells to nicotinamide, activin A, exendin 4, pentagastrin, HGF, B-27, and N-2 supplements.	[484]
Eight patients with early diagnosed T1D underwent immunoablation (high-dose cyclophosphamide and anti-thymocyte globulin) and hematopoietic stem cell transplantation (mobilized with cyclophosphamide and G-CSF). All patients became insulin-free after transplantation. One of them required a low insulin dose after 7 months, and six of the patients received the anti-diabetic drug acarbose (an alpha-glucosidase inhibitor) for better glycemic control.	[561]
Human placenta-derived MSCs were infused three times into 10 T2D patients at one-month intervals. The daily insulin requirement was reduced, and the serum C-peptide levels were increased along with reduced glycosylated hemoglobin. Also, the renal and cardiac functions were improved after MSC infusion.	[562]
Autologous hematopoietic stem cell transplantation (AHSCT) into 13 newly diagnosed T1D patients, among them 10 with diabetic ketoacidosis, resulted in lower insulin requirement in 11 of the individuals, accompanied by decreased IL-1, IL-17, and TNFα serum levels, increased C-peptide concentration and reduced glycosylated hemoglobin. Three patients become insulin-free for 7–54 months.	[563]
28 T1D patients underwent autologous nonmyeloablative hematopoietic stem cell transplantation after pretreatment consisting of a combination of cyclophosphamide and anti-thymocyte globulin. Insulin independence was achieved in 15 out of 28 T1D patients over a period of 4 to 42 months, with a much higher response rate in patients without diabetic ketoacidosis (70.6% responding) than those suffering from diabetic ketoacidosis (27.3% responding).	[564]
A case report of autologous hematopoietic stem cell transplantation (obtained by cyclophosphamide and G-CSF mobilization to blood) that was delivered to T1D patients with diabetic ketoacidosis together with subcutaneous administration of G-CSF to increase the peripheral blood neutrophil count. The insulin requirement was gradually reduced, and reached insulin independence after 27 days, which lasted more than 70 months. The treatment with cyclophosphamide was accompanied by nausea, vomiting, fever, alopecia, and leukopenia.	[565]
15 Newly onset T1D patients who were treated with Wharton’s jelly-derived MSCs showed a gradual reduction in blood glucose levels, increased fasting C-peptide levels, and reduced insulin requirement that lasted for more than 24 months. 4 of the 15 patients became insulin independent. 2 patients were non-responders. The HbA1c level was gradually reduced. The transplantation with Wharton’s jelly-derived MSCs does not require previous immunosuppression with cyclophosphamide and anti-thymocyte globulin, which is of great advantage.	[489]
Intrahepatic autologous bone marrow stem cell transplantation (stimulated with filgrastim (G-CSF)) was performed in two recently diagnosed T1D patients with ketoacidosis. These two patients showed increased levels of C-peptide and reduced blood glucose and HbA1c levels for at least 12 months of follow-up. The serum levels of anti-islet antibodies were strongly reduced by the stem cell transplantation.	[557]
Repeated transfusion of umbilical cord-derived MSCs resulted in increased C-peptide levels and increased number of regulatory T cells in a subgroup of T2D patients.	[566]
Autologous bone marrow-derived stem cell transplantation was introduced twice to 11 T2D patients. Nine out of the 11 (82%) reached a reduction of more than 50% in insulin requirement up to 12 months, with increased stimulated C-peptide serum levels.	[567]
Transplantation of hematopoietic stem cells to 65 newly onset T1D accompanied with immunosuppression therapy (anti-thymocyte globulin and cyclophosphamide) resulted in insulin independence in 59% of the patients for the first 6 months, and 32% remained insulin-independent after 48 months. All treated individuals showed a decrease in serum HbA1c levels and an increase in serum C-peptide levels.	[568]
Infusion of in vitro-generated donor bone marrow-derived hematopoietic stem cells to a 5-year-old T1D patient improved glucose homeostasis for 6 months. Infusion of in vitro-generated donor bone marrow-derived hematopoietic stem cells, together with autologous adipose tissue-derived MSCs that have been differentiated into insulin-producing cells, to a 9-year-old T1D patient resulted in stable blood glucose levels with reduced insulin requirement.	[569]
Two treatment groups were enrolled in this study. Group 1 included 10 T1D patients who received autologous MSC therapy from their own adipose tissue and bone marrow, while Group 2 included 10 T1D patients who received allogeneic MSC therapy obtained from healthy, compatible, non-diabetic volunteer donors. The adipose-derived MSCs were differentiated into insulin-producing cells using the protocol involving nicotinamide, activin A, exendin, pentagastrin, HGF, B27, and N2. Both groups showed reduced insulin requirement, reduced serum HbA1c levels, increased C-peptide serum levels, and reduced fasting blood glucose levels. Some of these parameters were significantly better in the autologous than in the allogeneic group. The MSC treatment also prevented episodes of diabetic ketoacidosis. These data show that adipose tissue-derived MSCs from T1D patients can be differentiated into insulin-producing cells that can be used to treat diabetes of the same individual.	[570]
Early onset T1D patients receiving autologous MSC transplantation showed preserved or increased C-peptide blood levels in response to a mixed-meal tolerance test (MMTT), indicative of preserved β-cell function.	[488]
T1D patients transplanted with umbilical cord-derived MSCs together with autologous bone marrow mononuclear cell stem cells showed increased serum insulin levels and reduced HbA1c levels. The fasting glucose level was reduced concomitant with a reduced insulin requirement.	[487]
24 T1D patients underwent immunoablation and autologous hematopoietic stem cell transplantation (AHSCT). 20 of the patients remained insulin-free for at least 9.5 months, with four remaining insulin-free for 34–80 months. No severe complications were observed, except for one patient who died of pseudomonas sepsis in the course of neutropenia after transplantation.	[571]
16 T1D patients got autologous hematopoietic stem cell transplantation following immunosuppressive regimens. 7 patients achieved insulin independence, six showed reduced insulin requirement, while 3 did not respond.	[572]
4 T1D patients with ketoacidosis were treated with bone marrow-derived MSCs. Two of the patients showed reduced insulin requirement, and one became insulin-independent for 3 months. The fourth patient remained stable on the same insulin dose for 4 years.	[573]
The immune responses after autologous hematopoietic stem cell transplantation were studied in 18 T1D patients. Patients who have received autologous hematopoietic stem cell transplantation showed increased fasting C-peptide levels, reduced serum HbA1c, and reduced insulin requirement. The Th1 cells from the peripheral blood of the stem cell transplanted patients secreted lower levels of IL-2, IL-12p40, and IFNγ. Stem cell transplantation reduced the proportion of Th17 cells while increasing the amount of regulatory T cells.	[556]
6 out of 10 T2D patients transplanted with either bone marrow-derived MSCs or mononuclear cells (MNCs) showed reduced insulin requirement. MSCs increased peripheral insulin sensitivity.	[574]
20 T1D patients received autologous hematopoietic stem cell transplantation. 14 of the patients developed insulin independence for 1.5 to 48 months. Thereafter, they returned to regular insulin use.	[575]
Transplantation of autologous MSCs to 5 T1D patients resulted in lower insulin requirements and increased leptin serum levels.	[576]
27 T1D patients received infusion of allogeneic umbilical cord-derived MSCs with a repeated MSC infusion after 3 months. 11 out of the 27 T1D patients maintained clinical remission after 1 year. 3 of the MSC transplanted T1D patients became transiently insulin independent. There was also a transient decrease in HbA1c serum levels.	[577]
11 T1D patients received autologous bone marrow-derived MSCs. Early transplantation of MSCs reduced the number of grade II hypoglycemic events. Early MSC transplantation increased the serum levels of IL-4 and increased the proportion of regulatory T cells. These authors found that early MSC transplantation has better clinical outcomes than late MSC transplantation.	[482]
A subgroup of T2D patients that got repeated infusion of umbilical cord-derived MSCs showed reduced HbA1c level and increased glucose infusion rate (GIR), with no overall improvement in islet β-cell function.	[578]
6 T1D children received autologous hematopoietic stem cell transplantation without immunoablation. The MSC transplantation resulted in lower blood glucose levels and HbA1c levels. There was also a decrease in the levels of auto-antibodies against islet cells (ICA), glutamic acid-decarboxylase (GAD), and islet antigen-related tyrosine phosphatase 2 (IA2).	[579]
A Phase I/II study treating recent onset T1D patients with allogeneic Wharton’s jelly MSCs showed that MSCs prevented the drop in serum C-peptide levels and prevented the increase in insulin requirement seen in the placebo group.	[490]

## Data Availability

Raw data are available upon reasonable request.

## Supplementary Materials

The following supporting information can be downloaded at: https://www.mdpi.com/article/10.3390/biomedicines11092558/s1.

## References

[1] Burrack A.L., Martinov T., Fife B.T. (2017). T cell-mediated β cell destruction: Autoimmunity and alloimmunity in the context of type 1 diabetes. Front. Endocrinol..

[2] Toren E., Burnette K.S., Banerjee R.R., Hunter C.S., Tse H.M. (2021). Partners in crime: β-cells and autoimmune responses complicit in type 1 diabetes pathogenesis. Front. Immunol..

[3] Eizirik D.L., Szymczak F., Mallone R. (2023). Why does the immune system destroy pancreatic β-cells but not α-cells in type 1 diabetes?. Nat. Rev. Endocrinol..

[4] Eizirik D.L., Pasquali L., Cnop M. (2020). Pancreatic β-cells in type 1 and type 2 diabetes mellitus: Different pathways to failure. Nat. Rev. Endocrinol..

[5] Hameed I., Masoodi S.R., Mir S.A., Nabi M., Ghazanfar K., Ganai B.A. (2015). Type 2 diabetes mellitus: From a metabolic disorder to an inflammatory condition. World J. Diabetes.

[6] Tinajero M.G., Malik V.S. (2021). An update on the epidemiology of type 2 diabetes: A global perspective. Endocrinol. Metab. Clin. N. Am..

[7] Quan W., Jo E.K., Lee M.S. (2013). Role of pancreatic β-cell death and inflammation in diabetes. Diabetes Obes. Metab..

[8] Weir G.C., Bonner-Weir S. (2013). Islet β cell mass in diabetes and how it relates to function, birth, and death. Ann. N. Y. Acad. Sci..

[9] Dalle S., Abderrahmani A., Renard E. (2023). Pharmacological inhibitors of β-cell dysfunction and death as therapeutics for diabetes. Front. Endocrinol..

[10] Rohm T.V., Meier D.T., Olefsky J.M., Donath M.Y. (2022). Inflammation in obesity, diabetes, and related disorders. Immunity.

[11] Chang A.M., Halter J.B. (2003). Aging and insulin secretion. Am. J. Physiol.-Endocrinol. Metab..

[12] Gumbiner B., Polonsky K.S., Beltz W.F., Wallace P., Brechtel G., Fink R.I. (1989). Effects of aging on insulin secretion. Diabetes.

[13] Møller N., Gormsen L., Fuglsang J., Gjedsted J. (2003). Effects of ageing on insulin secretion and action. Horm. Res. Paediatr..

[14] Bellary S., Kyrou I., Brown J.E., Bailey C.J. (2021). Type 2 diabetes mellitus in older adults: Clinical considerations and management. Nat. Rev. Endocrinol..

[15] Damond N., Engler S., Zanotelli V.R.T., Schapiro D., Wasserfall C.H., Kusmartseva I., Nick H.S., Thorel F., Herrera P.L., Atkinson M.A. (2019). A map of human type 1 diabetes progression by imaging mass cytometry. Cell Metab..

[16] Wang Y.J., Traum D., Schug J., Gao L., Liu C., Atkinson M.A., Powers A.C., Feldman M.D., Naji A., Chang K.M. (2019). Multiplexed in situ imaging mass cytometry analysis of the human endocrine pancreas and immune system in type 1 diabetes. Cell Metab..

[17] Coppieters K.T., Dotta F., Amirian N., Campbell P.D., Kay T.W., Atkinson M.A., Roep B.O., von Herrath M.G. (2012). Demonstration of islet-autoreactive CD8 T cells in insulitic lesions from recent onset and long-term type 1 diabetes patients. J. Exp. Med..

[18] Babon J.A., DeNicola M.E., Blodgett D.M., Crèvecoeur I., Buttrick T.S., Maehr R., Bottino R., Naji A., Kaddis J., Elyaman W. (2016). Analysis of self-antigen specificity of islet-infiltrating T cells from human donors with type 1 diabetes. Nat. Med..

[19] Lundberg M., Seiron P., Ingvast S., Korsgren O., Skog O. (2017). Insulitis in human diabetes: A histological evaluation of donor pancreases. Diabetologia.

[20] Apaolaza P.S., Balcacean D., Zapardiel-Gonzalo J., Rodriguez-Calvo T. (2023). The extent and magnitude of islet T cell infiltration as powerful tools to define the progression to type 1 diabetes. Diabetologia.

[21] Campbell-Thompson M.L., Atkinson M.A., Butler A.E., Chapman N.M., Frisk G., Gianani R., Giepmans B.N., von Herrath M.G., Hyöty H., Kay T.W. (2013). The diagnosis of insulitis in human type 1 diabetes. Diabetologia.

[22] Pratiwi C., Mokoagow M.I., Made Kshanti I.A., Soewondo P. (2020). The risk factors of inpatient hypoglycemia: A systematic review. Heliyon.

[23] Papachristoforou E., Lambadiari V., Maratou E., Makrilakis K. (2020). Association of glycemic indices (hyperglycemia, glucose variability, and hypoglycemia) with oxidative stress and diabetic complications. J. Diabetes Res..

[24] Stolar M. (2010). Glycemic control and complications in type 2 diabetes mellitus. Am. J. Med..

[25] Fowler M.J. (2011). Microvascular and macrovascular complications of diabetes. Clin. Diabetes.

[26] Martínez M.S., Manzano A., Olivar L.C., Nava M., Salazar J., D’Marco L., Ortiz R., Chacín M., Guerrero-Wyss M., Cabrera de Bravo M. (2021). The role of the α cell in the pathogenesis of diabetes: A World beyond the mirror. Int. J. Mol. Sci..

[27] Dunning B.E., Gerich J.E. (2007). The role of alpha-cell dysregulation in fasting and postprandial hyperglycemia in type 2 diabetes and therapeutic implications. Endocr. Rev..

[28] Brown R.J., Sinaii N., Rother K.I. (2008). Too much glucagon, too little insulin: Time course of pancreatic islet dysfunction in new-onset type 1 diabetes. Diabetes Care.

[29] Sherr J., Tsalikian E., Fox L., Buckingham B., Weinzimer S., Tamborlane W.V., White N.H., Arbelaez A.M., Kollman C., Ruedy K.J. (2014). Evolution of abnormal plasma glucagon responses to mixed-meal feedings in youth with type 1 diabetes during the first 2 years after diagnosis. Diabetes Care.

[30] Omar-Hmeadi M., Lund P.E., Gandasi N.R., Tengholm A., Barg S. (2020). Paracrine control of α-cell glucagon exocytosis is compromised in human type-2 diabetes. Nat. Commun..

[31] Elliott A.D., Ustione A., Piston D.W. (2015). Somatostatin and insulin mediate glucose-inhibited glucagon secretion in the pancreatic α-cell by lowering cAMP. Am. J. Physiol. Endocrinol. Metab..

[32] Wiedeman A.E., Muir V.S., Rosasco M.G., DeBerg H.A., Presnell S., Haas B., Dufort M.J., Speake C., Greenbaum C.J., Serti E. (2020). Autoreactive CD8^+^ T cell exhaustion distinguishes subjects with slow type 1 diabetes progression. J. Clin. Investig..

[33] Reddy S., Zeng N., Al-Diery H., Jung D., Yeu C., Joret M.O., Merrilees M.J., Wu F. (2015). Analysis of peri-islet CD45-positive leucocytic infiltrates in long-standing type 1 diabetic patients. Diabetologia.

[34] Lehuen A., Diana J., Zaccone P., Cooke A. (2010). Immune cell crosstalk in type 1 diabetes. Nat. Rev. Immunol..

[35] Rabinovitch A., Suarez-Pinzon W.L. (1998). Cytokines and their roles in pancreatic islet β-cell destruction and insulin-dependent diabetes mellitus. Biochem. Pharmacol..

[36] Gonzalez-Duque S., Azoury M.E., Colli M.L., Afonso G., Turatsinze J.V., Nigi L., Lalanne A.I., Sebastiani G., Carré A., Pinto S. (2018). Conventional and neo-antigenic peptides presented by β cells are targeted by circulating naïve CD8^+^ T cells in type 1 diabetic and healthy donors. Cell Metab..

[37] van Lummel M., van Veelen P.A., de Ru A.H., Janssen G.M., Pool J., Laban S., Joosten A.M., Nikolic T., Drijfhout J.W., Mearin M.L. (2016). Dendritic cells guide islet autoimmunity through a restricted and uniquely processed peptidome presented by high-risk HLA-DR. J. Immunol..

[38] Suk K., Kim S., Kim Y.H., Kim K.A., Chang I., Yagita H., Shong M., Lee M.S. (2001). IFN-γ/TNF-α synergism as the final effector in autoimmune diabetes: A key role for STAT1/IFN regulatory factor-1 pathway in pancreatic β cell death. J. Immunol..

[39] Nakayasu E.S., Syed F., Tersey S.A., Gritsenko M.A., Mitchell H.D., Chan C.Y., Dirice E., Turatsinze J.V., Cui Y., Kulkarni R.N. (2020). Comprehensive proteomics analysis of stressed human islets identifies GDF15 as a target for type 1 diabetes intervention. Cell Metab..

[40] Grunnet L.G., Aikin R., Tonnesen M.F., Paraskevas S., Blaabjerg L., Størling J., Rosenberg L., Billestrup N., Maysinger D., Mandrup-Poulsen T. (2009). Proinflammatory cytokines activate the intrinsic apoptotic pathway in β-cells. Diabetes.

[41] Melloul D. (2008). Role of NF-κB in β-cell death. Biochem. Soc. Trans..

[42] Prause M., Berchtold L.A., Urizar A.I., Trauelsen M.H., Billestrup N., Mandrup-Poulsen T., Størling J. (2016). TRAF2 mediates JNK and STAT3 activation in response to IL-1β and IFNγ and facilitates apoptotic death of insulin-producing β-cells. Mol. Cell. Endocrinol..

[43] Russell M.A., Morgan N.G. (2014). The impact of anti-inflammatory cytokines on the pancreatic β-cell. Islets.

[44] Eizirik D.L., Colli M.L., Ortis F. (2009). The role of inflammation in insulitis and β-cell loss in type 1 diabetes. Nat. Rev. Endocrinol..

[45] Ramos-Rodríguez M., Raurell-Vila H., Colli M.L., Alvelos M.I., Subirana-Granés M., Juan-Mateu J., Norris R., Turatsinze J.V., Nakayasu E.S., Webb-Robertson B.M. (2019). The impact of proinflammatory cytokines on the β-cell regulatory landscape provides insights into the genetics of type 1 diabetes. Nat. Genet..

[46] Gurzov E.N., Eizirik D.L. (2011). Bcl-2 proteins in diabetes: Mitochondrial pathways of β-cell death and dysfunction. Trends Cell Biol..

[47] Brozzi F., Nardelli T.R., Lopes M., Millard I., Barthson J., Igoillo-Esteve M., Grieco F.A., Villate O., Oliveira J.M., Casimir M. (2015). Cytokines induce endoplasmic reticulum stress in human, rat and mouse β cells via different mechanisms. Diabetologia.

[48] Jayasinghe M., Prathiraja O., Perera P.B., Jena R., Silva M.S., Weerawarna P.S.H., Singhal M., Kayani A.M.A., Karnakoti S., Jain S. (2022). The role of mesenchymal stem cells in the treatment of type 1 diabetes. Cureus.

[49] Shapiro A.M., Lakey J.R., Ryan E.A., Korbutt G.S., Toth E., Warnock G.L., Kneteman N.M., Rajotte R.V. (2000). Islet transplantation in seven patients with type 1 diabetes mellitus using a glucocorticoid-free immunosuppressive regimen. N. Engl. J. Med..

[50] Sutherland A., Marson L. (2021). Pancreas transplantation. Nephrol. Dial. Transplant..

[51] Ricordi C., Strom T.B. (2004). Clinical islet transplantation: Advances and immunological challenges. Nat. Rev. Immunol..

[52] Brennan D.C., Kopetskie H.A., Sayre P.H., Alejandro R., Cagliero E., Shapiro A.M., Goldstein J.S., DesMarais M.R., Booher S., Bianchine P.J. (2016). Long-term follow-up of the Edmonton protocol of islet transplantation in the United States. Am. J. Transplant..

[53] Shapiro A.M., Pokrywczynska M., Ricordi C. (2017). Clinical pancreatic islet transplantation. Nat. Rev. Endocrinol..

[54] Yan L.L., Ye L.P., Chen Y.H., He S.Q., Zhang C.Y., Mao X.L., Li S.W. (2022). The influence of microenvironment on survival of intraportal transplanted islets. Front. Immunol..

[55] Llacua L.A., Faas M.M., de Vos P. (2018). Extracellular matrix molecules and their potential contribution to the function of transplanted pancreatic islets. Diabetologia.

[56] Chen C., Rong P., Yang M., Ma X., Feng Z., Wang W. (2020). The role of interleukin-1β in destruction of transplanted islets. Cell Transplant..

[57] Marfil-Garza B.A., Imes S., Verhoeff K., Hefler J., Lam A., Dajani K., Anderson B., O’Gorman D., Kin T., Bigam D. (2022). Pancreatic islet transplantation in type 1 diabetes: 20-year experience from a single-centre cohort in Canada. Lancet Diabetes Endocrinol..

[58] Gibly R.F., Graham J.G., Luo X., Lowe W.L., Hering B.J., Shea L.D. (2011). Advancing islet transplantation: From engraftment to the immune response. Diabetologia.

[59] Bruni A., Bornstein S., Linkermann A., Shapiro A.M.J. (2018). Regulated cell death seen through the lens of islet transplantation. Cell Transplant..

[60] Kale A., Rogers N.M. (2023). No time to die-How islets meet their demise in transplantation. Cells.

[61] Pittenger M.F., Mackay A.M., Beck S.C., Jaiswal R.K., Douglas R., Mosca J.D., Moorman M.A., Simonetti D.W., Craig S., Marshak D.R. (1999). Multilineage potential of adult human mesenchymal stem cells. Science.

[62] Shrestha M., Nguyen T.T., Park J., Choi J.U., Yook S., Jeong J.H. (2021). Immunomodulation effect of mesenchymal stem cells in islet transplantation. Biomed. Pharmacother..

[63] Rackham C.L., Dhadda P.K., Chagastelles P.C., Simpson S.J., Dattani A.A., Bowe J.E., Jones P.M., King A.J. (2013). Pre-culturing islets with mesenchymal stromal cells using a direct contact configuration is beneficial for transplantation outcome in diabetic mice. Cytotherapy.

[64] Da Silva Xavier G. (2018). The cells of the islets of Langerhans. J. Clin. Med..

[65] Ionescu-Tirgoviste C., Gagniuc P.A., Gubceac E., Mardare L., Popescu I., Dima S., Militaru M. (2015). A 3D map of the islet routes throughout the healthy human pancreas. Sci. Rep..

[66] Bosco D., Armanet M., Morel P., Niclauss N., Sgroi A., Muller Y.D., Giovannoni L., Parnaud G., Berney T. (2010). Unique arrangement of alpha- and beta-cells in human islets of Langerhans. Diabetes.

[67] Almaça J., Weitz J., Rodriguez-Diaz R., Pereira E., Caicedo A. (2018). The pericyte of the pancreatic islet regulates capillary diameter and local blood flow. Cell Metab..

[68] Farhat B., Almelkar A., Ramachandran K., Williams S.J., Huang H.H., Zamierowksi D., Novikova L., Stehno-Bittel L. (2013). Small human islets comprised of more β-cells with higher insulin content than large islets. Islets.

[69] Huang H.H., Novikova L., Williams S.J., Smirnova I.V., Stehno-Bittel L. (2011). Low insulin content of large islet population is present in situ and in isolated islets. Islets.

[70] Peiris H., Bonder C.S., Coates P.T., Keating D.J., Jessup C.F. (2014). The β-cell/EC axis: How do islet cells talk to each other?. Diabetes.

[71] Bonner-Weir S., Sullivan B.A., Weir G.C. (2015). Human islet morphology revisited: Human and rodent islets are not so different after all. J. Histochem. Cytochem..

[72] Brissova M., Shostak A., Shiota M., Wiebe P.O., Poffenberger G., Kantz J., Chen Z., Carr C., Jerome W.G., Chen J. (2006). Pancreatic islet production of vascular endothelial growth factor-A is essential for islet vascularization, revascularization, and function. Diabetes.

[73] Eberhard D., Kragl M., Lammert E. (2010). ‘Giving and taking’: Endothelial and β-cells in the islets of Langerhans. Trends Endocrinol. Metab..

[74] Nikolova G., Jabs N., Konstantinova I., Domogatskaya A., Tryggvason K., Sorokin L., Fässler R., Gu G., Gerber H.P., Ferrara N. (2006). The vascular basement membrane: A niche for insulin gene expression and β cell proliferation. Dev. Cell.

[75] Otonkoski T., Banerjee M., Korsgren O., Thornell L.E., Virtanen I. (2008). Unique basement membrane structure of human pancreatic islets: Implications for beta-cell growth and differentiation. Diabetes Obes. Metab..

[76] Park H.S., Kim H.Z., Park J.S., Lee J., Lee S.P., Kim H., Ahn C.W., Nakaoka Y., Koh G.Y., Kang S. (2019). β-cell-derived angiopoietin-1 regulates insulin secretion and glucose homeostasis by stabilizing the islet microenvironment. Diabetes.

[77] Gan W.J., Do O.H., Cottle L., Ma W., Kosobrodova E., Cooper-White J., Bilek M., Thorn P. (2018). Local integrin activation in pancreatic β cells targets insulin secretion to the vasculature. Cell Rep..

[78] Sionov R.V., Finesilver G., Sapozhnikov L., Soroker A., Zlotkin-Rivkin E., Saad Y., Kahana M., Bodaker M., Alpert E., Mitrani E. (2015). β cells secrete significant and regulated levels of insulin for long periods when seeded onto acellular micro-scaffolds. Tissue Eng. Part A.

[79] Abualhassan N., Sapozhnikov L., Pawlick R.L., Kahana M., Pepper A.R., Bruni A., Gala-Lopez B., Kin T., Mitrani E., Shapiro A.M. (2016). Lung-derived microscaffolds facilitate diabetes reversal after mouse and human intraperitoneal islet transplantation. PLoS ONE.

[80] Llacua A., de Haan B.J., Smink S.A., de Vos P. (2016). Extracellular matrix components supporting human islet function in alginate-based immunoprotective microcapsules for treatment of diabetes. J. Biomed. Mater. Res. A.

[81] Weber L.M., Hayda K.N., Anseth K.S. (2008). Cell-matrix interactions improve β-cell survival and insulin secretion in three-dimensional culture. Tissue Eng. Part A.

[82] Llacua L.A., de Haan B.J., de Vos P. (2018). Laminin and collagen IV inclusion in immunoisolating microcapsules reduces cytokine-mediated cell death in human pancreatic islets. J. Tissue Eng. Regen. Med..

[83] Thorens B. (2011). Brain glucose sensing and neural regulation of insulin and glucagon secretion. Diabetes Obes. Metab..

[84] Caicedo A. (2013). Paracrine and autocrine interactions in the human islet: More than meets the eye. Semin. Cell Dev. Biol..

[85] Faber C.L., Deem J.D., Campos C.A., Taborsky G.J., Morton G.J. (2020). CNS control of the endocrine pancreas. Diabetologia.

[86] Hampton R.F., Jimenez-Gonzalez M., Stanley S.A. (2022). Unravelling innervation of pancreatic islets. Diabetologia.

[87] Alonge K.M., Porte D., Schwartz M.W. (2023). Distinct roles for brain and pancreas in basal and postprandial glucose homeostasis. Diabetes.

[88] Richardson T.M., Saunders D.C., Haliyur R., Shrestha S., Cartailler J.P., Reinert R.B., Petronglo J., Bottino R., Aramandla R., Bradley A.M. (2023). Human pancreatic capillaries and nerve fibers persist in type 1 diabetes despite β cell loss. Am. J. Physiol. Endocrinol. Metab..

[89] Thorens B. (2014). Neural regulation of pancreatic islet cell mass and function. Diabetes Obes. Metab..

[90] Jin Z., Korol S.V. (2023). GABA signalling in human pancreatic islets. Front. Endocrinol..

[91] Yi Z., Waseem Ghani M., Ghani H., Jiang W., Waseem Birmani M., Ye L., Bin L., Cun L.G., Lilong A., Mei X. (2020). Gimmicks of gamma-aminobutyric acid (GABA) in pancreatic β-cell regeneration through transdifferentiation of pancreatic α- to β-cells. Cell Biol. Int..

[92] Ben-Othman N., Vieira A., Courtney M., Record F., Gjernes E., Avolio F., Hadzic B., Druelle N., Napolitano T., Navarro-Sanz S. (2017). Long-term GABA administration induces alpha cell-mediated beta-like cell neogenesis. Cell.

[93] Tian J., Dang H., Chen Z., Guan A., Jin Y., Atkinson M.A., Kaufman D.L. (2013). γ-Aminobutyric acid regulates both the survival and replication of human β-cells. Diabetes.

[94] Lammert E., Thorn P. (2020). The role of the islet niche on β cell structure and function. J. Mol. Biol..

[95] Briant L.J.B., Reinbothe T.M., Spiliotis I., Miranda C., Rodriguez B., Rorsman P. (2018). δ-cells and β-cells are electrically coupled and regulate α-cell activity via somatostatin. J. Physiol..

[96] Meissner H.P. (1976). Electrophysiological evidence for coupling between β cells of pancreatic islets. Nature.

[97] Overton D.L., Mastracci T.L. (2022). Exocrine-endocrine crosstalk: The influence of pancreatic cellular communications on organ growth, function and disease. Front. Endocrinol..

[98] Benninger R.K., Piston D.W. (2014). Cellular communication and heterogeneity in pancreatic islet insulin secretion dynamics. Trends Endocrinol. Metab..

[99] Benninger R.K., Head W.S., Zhang M., Satin L.S., Piston D.W. (2011). Gap junctions and other mechanisms of cell-cell communication regulate basal insulin secretion in the pancreatic islet. J. Physiol..

[100] Ravier M.A., Güldenagel M., Charollais A., Gjinovci A., Caille D., Söhl G., Wollheim C.B., Willecke K., Henquin J.C., Meda P. (2005). Loss of connexin36 channels alters β-cell coupling, islet synchronization of glucose-induced Ca^2+^ and insulin oscillations, and basal insulin release. Diabetes.

[101] Loppini A., Braun M., Filippi S., Pedersen M.G. (2015). Mathematical modeling of gap junction coupling and electrical activity in human β-cells. Phys. Biol..

[102] Kawamori D., Kurpad A.J., Hu J., Liew C.W., Shih J.L., Ford E.L., Herrera P.L., Polonsky K.S., McGuinness O.P., Kulkarni R.N. (2009). Insulin signaling in α cells modulates glucagon secretion in vivo. Cell Metab..

[103] Kawamori D., Akiyama M., Hu J., Hambro B., Kulkarni R.N. (2011). Growth factor signalling in the regulation of α-cell fate. Diabetes Obes. Metab..

[104] Xu E., Kumar M., Zhang Y., Ju W., Obata T., Zhang N., Liu S., Wendt A., Deng S., Ebina Y. (2006). Intra-islet insulin suppresses glucagon release via GABA-GABAA receptor system. Cell Metab..

[105] Wang M.Y., Dean E.D., Quittner-Strom E., Zhu Y., Chowdhury K.H., Zhang Z., Zhao S., Li N., Ye R., Lee Y. (2021). Glucagon blockade restores functional β-cell mass in type 1 diabetic mice and enhances function of human islets. Proc. Natl. Acad. Sci. USA.

[106] Hutchens T., Piston D.W. (2015). EphA4 receptor forward signaling inhibits glucagon secretion from α-cells. Diabetes.

[107] Hughes J.W., Ustione A., Lavagnino Z., Piston D.W. (2018). Regulation of islet glucagon secretion: Beyond calcium. Diabetes Obes. Metab..

[108] Konstantinova I., Nikolova G., Ohara-Imaizumi M., Meda P., Kucera T., Zarbalis K., Wurst W., Nagamatsu S., Lammert E. (2007). EphA-Ephrin-A-mediated β cell communication regulates insulin secretion from pancreatic islets. Cell.

[109] Volta F., Scerbo M.J., Seelig A., Wagner R., O’Brien N., Gerst F., Fritsche A., Häring H.U., Zeigerer A., Ullrich S. (2019). Glucose homeostasis is regulated by pancreatic β-cell cilia via endosomal EphA-processing. Nat. Commun..

[110] Wang C., Guan Y., Yang J. (2010). Cytokines in the progression of pancreatic β-cell dysfunction. Int. J. Endocrinol..

[111] Cieślak M., Wojtczak A., Cieślak M. (2015). Role of pro-inflammatory cytokines of pancreatic islets and prospects of elaboration of new methods for the diabetes treatment. Acta Biochim. Pol..

[112] Eizirik D.L., Mandrup-Poulsen T. (2001). A choice of death—The signal-transduction of immune-mediated β-cell apoptosis. Diabetologia.

[113] Berchtold L.A., Prause M., Størling J., Mandrup-Poulsen T. (2016). Cytokines and pancreatic β-cell apoptosis. Adv. Clin. Chem..

[114] Ortis F., Naamane N., Flamez D., Ladrière L., Moore F., Cunha D.A., Colli M.L., Thykjaer T., Thorsen K., Orntoft T.F. (2010). Cytokines interleukin-1β and tumor necrosis factor-α regulate different transcriptional and alternative splicing networks in primary β-cells. Diabetes.

[115] Kaminitz A., Stein J., Yaniv I., Askenasy N. (2007). The vicious cycle of apoptotic β-cell death in type 1 diabetes. Immunol. Cell Biol..

[116] Kreuwel H.T., Morgan D.J., Krahl T., Ko A., Sarvetnick N., Sherman L.A. (1999). Comparing the relative role of perforin/granzyme versus Fas/Fas ligand cytotoxic pathways in CD8^+^ T cell-mediated insulin-dependent diabetes mellitus. J. Immunol..

[117] Kägi D., Ho A., Odermatt B., Zakarian A., Ohashi P.S., Mak T.W. (1999). TNF receptor 1-dependent β cell toxicity as an effector pathway in autoimmune diabetes. J. Immunol..

[118] Savinov A.Y., Tcherepanov A., Green E.A., Flavell R.A., Chervonsky A.V. (2003). Contribution of Fas to diabetes development. Proc. Natl. Acad. Sci. USA.

[119] Burke S.J., Stadler K., Lu D., Gleason E., Han A., Donohoe D.R., Rogers R.C., Hermann G.E., Karlstad M.D., Collier J.J. (2015). IL-1β reciprocally regulates chemokine and insulin secretion in pancreatic β-cells via NF-κB. Am. J. Physiol. Endocrinol. Metab..

[120] Collier J.J., Sparer T.E., Karlstad M.D., Burke S.J. (2017). Pancreatic islet inflammation: An emerging role for chemokines. J. Mol. Endocrinol..

[121] Eizirik D.L., Moore F., Flamez D., Ortis F. (2008). Use of a systems biology approach to understand pancreatic β-cell death in Type 1 diabetes. Biochem. Soc. Trans..

[122] Mandrup-Poulsen T., Bendtzen K., Dinarello C.A., Nerup J. (1987). Human tumor necrosis factor potentiates human interleukin 1-mediated rat pancreatic β-cell cytotoxicity. J. Immunol..

[123] Pukel C., Baquerizo H., Rabinovitch A. (1988). Destruction of rat islet cell monolayers by cytokines. Synergistic interactions of interferon-gamma, tumor necrosis factor, lymphotoxin, and interleukin 1. Diabetes.

[124] Rabinovitch A., Sumoski W., Rajotte R.V., Warnock G.L. (1990). Cytotoxic effects of cytokines on human pancreatic islet cells in monolayer culture. J. Clin. Endocrinol. Metab..

[125] Kim K.A., Lee M.S. (2009). Recent progress in research on β-cell apoptosis by cytokines. Front. Biosci. (Landmark Ed.).

[126] Thomas H.E., Irawaty W., Darwiche R., Brodnicki T.C., Santamaria P., Allison J., Kay T.W. (2004). IL-1 receptor deficiency slows progression to diabetes in the NOD mouse. Diabetes.

[127] Mastrandrea L., Yu J., Behrens T., Buchlis J., Albini C., Fourtner S., Quattrin T. (2009). Etanercept treatment in children with new-onset type 1 diabetes: Pilot randomized, placebo-controlled, double-blind study. Diabetes Care.

[128] Quattrin T., Haller M.J., Steck A.K., Felner E.I., Li Y., Xia Y., Leu J.H., Zoka R., Hedrick J.A., Rigby M.R. (2020). Golimumab and β-cell function in youth with new-onset type 1 diabetes. N. Engl. J. Med..

[129] Cnop M., Welsh N., Jonas J.C., Jörns A., Lenzen S., Eizirik D.L. (2005). Mechanisms of pancreatic β-cell death in type 1 and type 2 diabetes: Many differences, few similarities. Diabetes.

[130] Ortis F., Pirot P., Naamane N., Kreins A.Y., Rasschaert J., Moore F., Théâtre E., Verhaeghe C., Magnusson N.E., Chariot A. (2008). Induction of nuclear factor-κB and its downstream genes by TNF-α and IL-1β has a pro-apoptotic role in pancreatic β cells. Diabetologia.

[131] Ortis F., Miani M., Colli M.L., Cunha D.A., Gurzov E.N., Allagnat F., Chariot A., Eizirik D.L. (2012). Differential usage of NF-κB activating signals by IL-1β and TNF-α in pancreatic beta cells. FEBS Lett..

[132] Meyerovich K., Fukaya M., Terra L.F., Ortis F., Eizirik D.L., Cardozo A.K. (2016). The non-canonical NF-κB pathway is induced by cytokines in pancreatic β cells and contributes to cell death and proinflammatory responses in vitro. Diabetologia.

[133] Ortis F., Cardozo A.K., Crispim D., Störling J., Mandrup-Poulsen T., Eizirik D.L. (2006). Cytokine-induced proapoptotic gene expression in insulin-producing cells is related to rapid, sustained, and nonoscillatory nuclear factor-kappaB activation. Mol. Endocrinol..

[134] Moore F., Naamane N., Colli M.L., Bouckenooghe T., Ortis F., Gurzov E.N., Igoillo-Esteve M., Mathieu C., Bontempi G., Thykjaer T. (2011). STAT1 is a master regulator of pancreatic β-cell apoptosis and islet inflammation. J. Biol. Chem..

[135] Eldor R., Yeffet A., Baum K., Doviner V., Amar D., Ben-Neriah Y., Christofori G., Peled A., Carel J.C., Boitard C. (2006). Conditional and specific NF-κB blockade protects pancreatic β cells from diabetogenic agents. Proc. Natl. Acad. Sci. USA.

[136] Eldor R., Baum K., Abel R., Sever D., Melloul D. (2009). The ToI-β transgenic mouse: A model to study the specific role of NF-κB in β-cells. Diabetes Res. Clin. Pract..

[137] Rink J.S., Chen X., Zhang X., Kaufman D.B. (2012). Conditional and specific inhibition of NF-κB in mouse pancreatic β cells prevents cytokine-induced deleterious effects and improves islet survival posttransplant. Surgery.

[138] Heimberg H., Heremans Y., Jobin C., Leemans R., Cardozo A.K., Darville M., Eizirik D.L. (2001). Inhibition of cytokine-induced NF-kappaB activation by adenovirus-mediated expression of a NF-kappaB super-repressor prevents β-cell apoptosis. Diabetes.

[139] Cardozo A.K., Heimberg H., Heremans Y., Leeman R., Kutlu B., Kruhøffer M., Ørntoft T., Eizirik D.L. (2001). A comprehensive analysis of cytokine-induced and nuclear factor-kappa B-dependent genes in primary rat pancreatic β-cells. J. Biol. Chem..

[140] Stancill J.S., Kasmani M.Y., Khatun A., Cui W., Corbett J.A. (2022). Cytokine and nitric oxide-dependent gene regulation in islet endocrine and nonendocrine cells. Function.

[141] Ammendrup A., Maillard A., Nielsen K., Aabenhus Andersen N., Serup P., Dragsbaek Madsen O., Mandrup-Poulsen T., Bonny C. (2000). The c-Jun amino-terminal kinase pathway is preferentially activated by interleukin-1 and controls apoptosis in differentiating pancreatic beta-cells. Diabetes.

[142] Saldeen J., Lee J.C., Welsh N. (2001). Role of p38 mitogen-activated protein kinase (p38 MAPK) in cytokine-induced rat islet cell apoptosis. Biochem. Pharmacol..

[143] Cardozo A.K., Kruhøffer M., Leeman R., Orntoft T., Eizirik D.L. (2001). Identification of novel cytokine-induced genes in pancreatic β-cells by high-density oligonucleotide arrays. Diabetes.

[144] Kutlu B., Cardozo A.K., Darville M.I., Kruhøffer M., Magnusson N., Ørntoft T., Eizirik D.L. (2003). Discovery of gene networks regulating cytokine-induced dysfunction and apoptosis in insulin-producing INS-1 cells. Diabetes.

[145] Magnusson N.E., Cardozo A.K., Kruhøffer M., Eizirik D.L., Ørntoft T.F., Jensen J.L. (2005). Construction and validation of the APOCHIP, a spotted oligo-microarray for the study of β-cell apoptosis. BMC Bioinform..

[146] Eizirik D.L., Sammeth M., Bouckenooghe T., Bottu G., Sisino G., Igoillo-Esteve M., Ortis F., Santin I., Colli M.L., Barthson J. (2012). The human pancreatic islet transcriptome: Expression of candidate genes for type 1 diabetes and the impact of pro-inflammatory cytokines. PLoS Genet..

[147] Ylipaasto P., Kutlu B., Rasilainen S., Rasschaert J., Salmela K., Teerijoki H., Korsgren O., Lahesmaa R., Hovi T., Eizirik D.L. (2005). Global profiling of coxsackievirus- and cytokine-induced gene expression in human pancreatic islets. Diabetologia.

[148] Lopes M., Kutlu B., Miani M., Bang-Berthelsen C.H., Størling J., Pociot F., Goodman N., Hood L., Welsh N., Bontempi G. (2014). Temporal profiling of cytokine-induced genes in pancreatic β-cells by meta-analysis and network inference. Genomics.

[149] Corbett J.A., Kwon G., Turk J., McDaniel M.L. (1993). IL-1β induces the coexpression of both nitric oxide synthase and cyclooxygenase by islets of Langerhans: Activation of cyclooxygenase by nitric oxide. Biochemistry.

[150] Kwon G., Corbett J.A., Hauser S., Hill J.R., Turk J., McDaniel M.L. (1998). Evidence for involvement of the proteasome complex (26S) and NFκB in IL-1β-induced nitric oxide and prostaglandin production by rat islets and RINm5F cells. Diabetes.

[151] Kwon G., Corbett J.A., Rodi C.P., Sullivan P., McDaniel M.L. (1995). Interleukin-1β-induced nitric oxide synthase expression by rat pancreatic β-cells: Evidence for the involvement of nuclear factor kappa B in the signaling mechanism. Endocrinology.

[152] Darville M.I., Eizirik D.L. (1998). Regulation by cytokines of the inducible nitric oxide synthase promoter in insulin-producing cells. Diabetologia.

[153] Flodström M., Welsh N., Eizirik D.L. (1996). Cytokines activate the nuclear factor kappa B (NF-κB) and induce nitric oxide production in human pancreatic islets. FEBS Lett..

[154] Karlsen A.E., Pavlovic D., Nielsen K., Jensen J., Andersen H.U., Pociot F., Mandrup-Poulsen T., Eizirik D.L., Nerup J. (2000). Interferon-gamma induces interleukin-1 converting enzyme expression in pancreatic islets by an interferon regulatory factor-1-dependent mechanism. J. Clin. Endocrinol. Metab..

[155] Chen M.C., Schuit F., Eizirik D.L. (1999). Identification of IL-1β-induced messenger RNAs in rat pancreatic β cells by differential display of messenger RNA. Diabetologia.

[156] Cottet S., Dupraz P., Hamburger F., Dolci W., Jaquet M., Thorens B. (2002). cFLIP protein prevents tumor necrosis factor-α-mediated induction of caspase-8-dependent apoptosis in insulin-secreting betaTc-Tet cells. Diabetes.

[157] Barthson J., Germano C.M., Moore F., Maida A., Drucker D.J., Marchetti P., Gysemans C., Mathieu C., Nuñez G., Jurisicova A. (2011). Cytokines tumor necrosis factor-α and interferon-γ induce pancreatic β-cell apoptosis through STAT1-mediated Bim protein activation. J. Biol. Chem..

[158] Gurzov E.N., Germano C.M., Cunha D.A., Ortis F., Vanderwinden J.M., Marchetti P., Zhang L., Eizirik D.L. (2010). p53 up-regulated modulator of apoptosis (PUMA) activation contributes to pancreatic β-cell apoptosis induced by proinflammatory cytokines and endoplasmic reticulum stress. J. Biol. Chem..

[159] Ren D., Tu H.C., Kim H., Wang G.X., Bean G.R., Takeuchi O., Jeffers J.R., Zambetti G.P., Hsieh J.J., Cheng E.H. (2010). BID, BIM, and PUMA are essential for activation of the BAX- and BAK-dependent cell death program. Science.

[160] Allagnat F., Cunha D., Moore F., Vanderwinden J.M., Eizirik D.L., Cardozo A.K. (2011). Mcl-1 downregulation by pro-inflammatory cytokines and palmitate is an early event contributing to β-cell apoptosis. Cell Death Differ..

[161] Eizirik D.L., Sandler S., Welsh N., Cetkovic-Cvrlje M., Nieman A., Geller D.A., Pipeleers D.G., Bendtzen K., Hellerström C. (1994). Cytokines suppress human islet function irrespective of their effects on nitric oxide generation. J. Clin. Investig..

[162] Pirot P., Ortis F., Cnop M., Ma Y., Hendershot L.M., Eizirik D.L., Cardozo A.K. (2007). Transcriptional regulation of the endoplasmic reticulum stress gene CHOP in pancreatic insulin-producing cells. Diabetes.

[163] Allagnat F., Fukaya M., Nogueira T.C., Delaroche D., Welsh N., Marselli L., Marchetti P., Haefliger J.A., Eizirik D.L., Cardozo A.K. (2012). C/EBP homologous protein contributes to cytokine-induced pro-inflammatory responses and apoptosis in β-cells. Cell Death Differ..

[164] Seiron P., Stenwall A., Hedin A., Granlund L., Esguerra J.L.S., Volkov P., Renström E., Korsgren O., Lundberg M., Skog O. (2021). Transcriptional analysis of islets of Langerhans from organ donors of different ages. PLoS ONE.

[165] Trindade B.C., Chen G.Y. (2020). NOD1 and NOD2 in inflammatory and infectious diseases. Immunol. Rev..

[166] Fantuzzi G., Dinarello C.A. (1999). Interleukin-18 and interleukin-1β: Two cytokine substrates for ICE (caspase-1). J. Clin. Immunol..

[167] Martinon F., Burns K., Tschopp J. (2002). The inflammasome: A molecular platform triggering activation of inflammatory caspases and processing of proIL-β. Mol. Cell.

[168] Pearson J.A., Wong F.S., Wen L. (2021). Inflammasomes and type 1 diabetes. Front. Immunol..

[169] Strowig T., Henao-Mejia J., Elinav E., Flavell R. (2012). Inflammasomes in health and disease. Nature.

[170] Schroder K., Tschopp J. (2010). The inflammasomes. Cell.

[171] Zheng D., Liwinski T., Elinav E. (2020). Inflammasome activation and regulation: Toward a better understanding of complex mechanisms. Cell Discov..

[172] Sollberger G., Strittmatter G.E., Kistowska M., French L.E., Beer H.D. (2012). Caspase-4 is required for activation of inflammasomes. J. Immunol..

[173] Carlos D., Costa F.R., Pereira C.A., Rocha F.A., Yaochite J.N., Oliveira G.G., Carneiro F.S., Tostes R.C., Ramos S.G., Zamboni D.S. (2017). Mitochondrial DNA activates the NLRP3 inflammasome and predisposes to type 1 diabetes in murine model. Front. Immunol..

[174] Tschopp J., Schroder K. (2010). NLRP3 inflammasome activation: The convergence of multiple signalling pathways on ROS production?. Nat. Rev. Immunol..

[175] Zhou R., Tardivel A., Thorens B., Choi I., Tschopp J. (2010). Thioredoxin-interacting protein links oxidative stress to inflammasome activation. Nat. Immunol..

[176] Thome M., Hofmann K., Burns K., Martinon F., Bodmer J.L., Mattmann C., Tschopp J. (1998). Identification of CARDIAK, a RIP-like kinase that associates with caspase-1. Curr. Biol..

[177] Zhang W.H., Wang X., Narayanan M., Zhang Y., Huo C., Reed J.C., Friedlander R.M. (2003). Fundamental role of the Rip2/caspase-1 pathway in hypoxia and ischemia-induced neuronal cell death. Proc. Natl. Acad. Sci. USA.

[178] McCarthy J.V., Ni J., Dixit V.M. (1998). RIP2 is a novel NF-κB-activating and cell death-inducing kinase. J. Biol. Chem..

[179] Bergsbaken T., Fink S.L., Cookson B.T. (2009). Pyroptosis: Host cell death and inflammation. Nat. Rev. Microbiol..

[180] Cookson B.T., Brennan M.A. (2001). Pro-inflammatory programmed cell death. Trends Microbiol..

[181] Liu X., Zhang Z., Ruan J., Pan Y., Magupalli V.G., Wu H., Lieberman J. (2016). Inflammasome-activated gasdermin D causes pyroptosis by forming membrane pores. Nature.

[182] Anderson M.J., den Hartigh A.B., Fink S.L. (2023). Molecular mechanisms of pyroptosis. Methods Mol. Biol..

[183] Miao E.A., Leaf I.A., Treuting P.M., Mao D.P., Dors M., Sarkar A., Warren S.E., Wewers M.D., Aderem A. (2010). Caspase-1-induced pyroptosis is an innate immune effector mechanism against intracellular bacteria. Nat. Immunol..

[184] Al Mamun A., Ara Mimi A., Wu Y., Zaeem M., Abdul Aziz M., Aktar Suchi S., Alyafeai E., Munir F., Xiao J. (2021). Pyroptosis in diabetic nephropathy. Clin. Chim. Acta.

[185] Hu C., Ding H., Li Y., Pearson J.A., Zhang X., Flavell R.A., Wong F.S., Wen L. (2015). NLRP3 deficiency protects from type 1 diabetes through the regulation of chemotaxis into the pancreatic islets. Proc. Natl. Acad. Sci. USA.

[186] Pontillo A., Brandao L., Guimaraes R., Segat L., Araujo J., Crovella S. (2010). Two SNPs in NLRP3 gene are involved in the predisposition to type-1 diabetes and celiac disease in a pediatric population from northeast Brazil. Autoimmunity.

[187] Sun X., Xia Y., Liu Y., Wang Y., Luo S., Lin J., Huang G., Li X., Xie Z., Zhou Z. (2019). Polymorphisms in NLRP1 gene are associated with type 1 diabetes. J. Diabetes Res..

[188] Makishima T., Yoshimi M., Komiyama S., Hara N., Nishimoto T. (2000). A subunit of the mammalian oligosaccharyltransferase, DAD1, interacts with Mcl-1, one of the Bcl-2 protein family. J. Biochem..

[189] Makishima T., Nakashima T., Nagata-Kuno K., Fukushima K., Iida H., Sakaguchi M., Ikehara Y., Komiyama S., Nishimoto T. (1997). The highly conserved DAD1 protein involved in apoptosis is required for N-linked glycosylation. Genes Cells.

[190] Hong N.A., Flannery M., Hsieh S.N., Cado D., Pedersen R., Winoto A. (2000). Mice lacking Dad1, the defender against apoptotic death-1, express abnormal N-linked glycoproteins and undergo increased embryonic apoptosis. Dev. Biol..

[191] Brewster J.L., Martin S.L., Toms J., Goss D., Wang K., Zachrone K., Davis A., Carlson G., Hood L., Coffin J.D. (2000). Deletion of Dad1 in mice induces an apoptosis-associated embryonic death. Genesis.

[192] Nishii K., Tsuzuki T., Kumai M., Takeda N., Koga H., Aizawa S., Nishimoto T., Shibata Y. (1999). Abnormalities of developmental cell death in Dad1-deficient mice. Genes Cells.

[193] Martens G.A., Pipeleers D. (2009). Glucose, regulator of survival and phenotype of pancreatic β cells. Vitam. Horm..

[194] Liuwantara D., Elliot M., Smith M.W., Yam A.O., Walters S.N., Marino E., McShea A., Grey S.T. (2006). Nuclear factor-kappaB regulates β-cell death: A critical role for A20 in beta-cell protection. Diabetes.

[195] Heyninck K., De Valck D., Vanden Berghe W., Van Criekinge W., Contreras R., Fiers W., Haegeman G., Beyaert R. (1999). The zinc finger protein A20 inhibits TNF-induced NF-kappaB-dependent gene expression by interfering with an RIP- or TRAF2-mediated transactivation signal and directly binds to a novel NF-kappaB-inhibiting protein ABIN. J. Cell Biol..

[196] Grey S.T., Arvelo M.B., Hasenkamp W., Bach F.H., Ferran C. (1999). A20 inhibits cytokine-induced apoptosis and nuclear factor kappaB-dependent gene activation in islets. J. Exp. Med..

[197] Zammit N.W., Walters S.N., Seeberger K.L., O’Connell P.J., Korbutt G.S., Grey S.T. (2019). A20 as an immune tolerance factor can determine islet transplant outcomes. JCI Insight.

[198] Burrows M.P., Volchkov P., Kobayashi K.S., Chervonsky A.V. (2015). Microbiota regulates type 1 diabetes through Toll-like receptors. Proc. Natl. Acad. Sci. USA.

[199] Nackiewicz D., Dan M., He W., Kim R., Salmi A., Rütti S., Westwell-Roper C., Cunningham A., Speck M., Schuster-Klein C. (2014). TLR2/6 and TLR4-activated macrophages contribute to islet inflammation and impair β cell insulin gene expression via IL-1 and IL-6. Diabetologia.

[200] Kim H.S., Han M.S., Chung K.W., Kim S., Kim E., Kim M.J., Jang E., Lee H.A., Youn J., Akira S. (2007). Toll-like receptor 2 senses β-cell death and contributes to the initiation of autoimmune diabetes. Immunity.

[201] Aliprantis A.O., Yang R.B., Weiss D.S., Godowski P., Zychlinsky A. (2000). The apoptotic signaling pathway activated by Toll-like receptor-2. EMBO J..

[202] Russell M.A., Cooper A.C., Dhayal S., Morgan N.G. (2013). Differential effects of interleukin-13 and interleukin-6 on Jak/STAT signaling and cell viability in pancreatic β-cells. Islets.

[203] Kaminski A., Welters H.J., Kaminski E.R., Morgan N.G. (2009). Human and rodent pancreatic β-cells express IL-4 receptors and IL-4 protects against β-cell apoptosis by activation of the PI3K and JAK/STAT pathways. Biosci. Rep..

[204] Rütti S., Howald C., Arous C., Dermitzakis E., Halban P.A., Bouzakri K. (2016). IL-13 improves β-cell survival and protects against IL-1β-induced β-cell death. Mol. Metab..

[205] Choi S.E., Choi K.M., Yoon I.H., Shin J.Y., Kim J.S., Park W.Y., Han D.J., Kim S.C., Ahn C., Kim J.Y. (2004). IL-6 protects pancreatic islet beta cells from pro-inflammatory cytokines-induced cell death and functional impairment in vitro and in vivo. Transpl. Immunol..

[206] Paula F.M., Leite N.C., Vanzela E.C., Kurauti M.A., Freitas-Dias R., Carneiro E.M., Boschero A.C., Zoppi C.C. (2015). Exercise increases pancreatic β-cell viability in a model of type 1 diabetes through IL-6 signaling. FASEB J..

[207] Cameron M.J., Arreaza G.A., Zucker P., Chensue S.W., Strieter R.M., Chakrabarti S., Delovitch T.L. (1997). IL-4 prevents insulitis and insulin-dependent diabetes mellitus in nonobese diabetic mice by potentiation of regulatory T helper-2 cell function. J. Immunol..

[208] Gallichan W.S., Balasa B., Davies J.D., Sarvetnick N. (1999). Pancreatic IL-4 expression results in islet-reactive Th2 cells that inhibit diabetogenic lymphocytes in the nonobese diabetic mouse. J. Immunol..

[209] Lu J., Liu J., Li L., Lan Y., Liang Y. (2020). Cytokines in type 1 diabetes: Mechanisms of action and immunotherapeutic targets. Clin. Transl. Immunol..

[210] Jensen J., Galsgaard E.D., Karlsen A.E., Lee Y.C., Nielsen J.H. (2005). STAT5 activation by human GH protects insulin-producing cells against interleukin-1β, interferon-γ and tumour necrosis factor-α-induced apoptosis independent of nitric oxide production. J. Endocrinol..

[211] Perez-Serna A.A., Dos Santos R.S., Ripoll C., Nadal A., Eizirik D.L., Marroqui L. (2023). BCL-XL overexpression protects pancreatic β-cells against cytokine- and palmitate-induced apoptosis. Int. J. Mol. Sci..

[212] Nano E., Petropavlovskaia M., Rosenberg L. (2021). Islet neogenesis associated protein (INGAP) protects pancreatic β cells from IL-1β and IFNγ-induced apoptosis. Cell Death Discov..

[213] Wei D., Li J., Shen M., Jia W., Chen N., Chen T., Su D., Tian H., Zheng S., Dai Y. (2010). Cellular production of n-3 PUFAs and reduction of n-6-to-n-3 ratios in the pancreatic β-cells and islets enhance insulin secretion and confer protection against cytokine-induced cell death. Diabetes.

[214] Atkinson M.A., Maclaren N.K., Luchetta R. (1990). Insulitis and diabetes in NOD mice reduced by prophylactic insulin therapy. Diabetes.

[215] Mabley J.G., Belin V., John N., Green I.C. (1997). Insulin-like growth factor I reverses interleukin-1beta inhibition of insulin secretion, induction of nitric oxide synthase and cytokine-mediated apoptosis in rat islets of Langerhans. FEBS Lett..

[216] Petrik J., Arany E., McDonald T.J., Hill D.J. (1998). Apoptosis in the pancreatic islet cells of the neonatal rat is associated with a reduced expression of insulin-like growth factor II that may act as a survival factor. Endocrinology.

[217] Hill D.J., Strutt B., Arany E., Zaina S., Coukell S., Graham C.F. (2000). Increased and persistent circulating insulin-like growth factor II in neonatal transgenic mice suppresses developmental apoptosis in the pancreatic islets. Endocrinology.

[218] Mellado-Gil J., Rosa T.C., Demirci C., Gonzalez-Pertusa J.A., Velazquez-Garcia S., Ernst S., Valle S., Vasavada R.C., Stewart A.F., Alonso L.C. (2011). Disruption of hepatocyte growth factor/c-Met signaling enhances pancreatic beta-cell death and accelerates the onset of diabetes. Diabetes.

[219] Arafat H.A., Katakam A.K., Chipitsyna G., Gong Q., Vancha A.R., Gabbeta J., Dafoe D.C. (2007). Osteopontin protects the islets and beta-cells from interleukin-1 beta-mediated cytotoxicity through negative feedback regulation of nitric oxide. Endocrinology.

[220] Sui M., Li T., Lu H., Li Y., Huang J., Zhang P., Wang S., Zeng L. (2023). SOCS3 inhibits the mesenchymal stromal cell secretory factor SDF-1-mediated improvement of islet function in non-obese diabetic mice. Stem Cell Res. Ther..

[221] Zhu Q., Jin J.F., Shan X.H., Liu C.P., Mao X.D., Xu K.F., Liu C. (2008). Chronic activation of neutral ceramidase protects β-cells against cytokine-induced apoptosis. Acta Pharmacol. Sin..

[222] Hammerle C.M., Sandovici I., Brierley G.V., Smith N.M., Zimmer W.E., Zvetkova I., Prosser H.M., Sekita Y., Lam B.Y.H., Ma M. (2020). Mesenchyme-derived IGF2 is a major paracrine regulator of pancreatic growth and function. PLoS Genet..

[223] Olerud J., Kanaykina N., Vasylovska S., King D., Sandberg M., Jansson L., Kozlova E.N. (2009). Neural crest stem cells increase beta cell proliferation and improve islet function in co-transplanted murine pancreatic islets. Diabetologia.

[224] Pingitore A., Caroleo M.C., Cione E., Castañera Gonzalez R., Huang G.C., Persaud S.J. (2016). Fine tuning of insulin secretion by release of nerve growth factor from mouse and human islet β-cells. Mol. Cell Endocrinol..

[225] Houtz J., Borden P., Ceasrine A., Minichiello L., Kuruvilla R. (2016). Neurotrophin signaling is required for glucose-induced insulin secretion. Dev. Cell.

[226] Nostro M.C., Sarangi F., Ogawa S., Holtzinger A., Corneo B., Li X., Micallef S.J., Park I.H., Basford C., Wheeler M.B. (2011). Stage-specific signaling through TGFβ family members and WNT regulates patterning and pancreatic specification of human pluripotent stem cells. Development.

[227] Bertolino P., Holmberg R., Reissmann E., Andersson O., Berggren P.O., Ibáñez C.F. (2008). Activin B receptor ALK7 is a negative regulator of pancreatic β-cell function. Proc. Natl. Acad. Sci. USA.

[228] Florio P., Luisi S., Marchetti P., Lupi R., Cobellis L., Falaschi C., Sugino H., Navalesi R., Genazzani A.R., Petraglia F. (2000). Activin A stimulates insulin secretion in cultured human pancreatic islets. J. Endocrinol. Investig..

[229] Totsuka Y., Tabuchi M., Kojima I., Shibai H., Ogata E. (1988). A novel action of activin A: Stimulation of insulin secretion in rat pancreatic islets. Biochem. Biophys. Res. Commun..

[230] Li L., Yi Z., Seno M., Kojima I. (2004). Activin A and betacellulin: Effect on regeneration of pancreatic β-cells in neonatal streptozotocin-treated rats. Diabetes.

[231] Gao Y., Zhang R., Dai S., Zhang X., Li X., Bai C. (2019). Role of TGF-β/Smad pathway in the transcription of pancreas-specific genes during β cell differentiation. Front. Cell Dev. Biol..

[232] Czubak P., Bojarska-Junak A., Tabarkiewicz J., Putowski L. (2014). A modified method of insulin producing cells’ generation from bone marrow-derived mesenchymal stem cells. J. Diabetes Res..

[233] D’Amour K.A., Agulnick A.D., Eliazer S., Kelly O.G., Kroon E., Baetge E.E. (2005). Efficient differentiation of human embryonic stem cells to definitive endoderm. Nat. Biotechnol..

[234] Hogrebe N.J., Maxwell K.G., Augsornworawat P., Millman J.R. (2021). Generation of insulin-producing pancreatic β cells from multiple human stem cell lines. Nat. Protoc..

[235] Pagliuca F.W., Millman J.R., Gürtler M., Segel M., Van Dervort A., Ryu J.H., Peterson Q.P., Greiner D., Melton D.A. (2014). Generation of functional human pancreatic β cells in vitro. Cell.

[236] Furukawa M., Eto Y., Kojima I. (1995). Expression of immunoreactive activin A in fetal rat pancreas. Endocr. J..

[237] Smart N.G., Apelqvist A.A., Gu X., Harmon E.B., Topper J.N., MacDonald R.J., Kim S.K. (2006). Conditional expression of Smad7 in pancreatic beta cells disrupts TGF-β signaling and induces reversible diabetes mellitus. PLoS Biol..

[238] Karanth S.S., Sun S., Bi H., Ye K., Jin S. (2021). Angiopoietins stimulate pancreatic islet development from stem cells. Sci. Rep..

[239] Hanyu O., Yamatani K., Ikarashi T., Soda S., Maruyama S., Kamimura T., Kaneko S., Hirayama S., Suzuki K., Nakagawa O. (2003). Brain-derived neurotrophic factor modulates glucagon secretion from pancreatic α cells: Its contribution to glucose metabolism. Diabetes Obes. Metab..

[240] Yamanaka M., Itakura Y., Inoue T., Tsuchida A., Nakagawa T., Noguchi H., Taiji M. (2006). Protective effect of brain-derived neurotrophic factor on pancreatic islets in obese diabetic mice. Metabolism.

[241] Tonra J.R., Ono M., Liu X., Garcia K., Jackson C., Yancopoulos G.D., Wiegand S.J., Wong V. (1999). Brain-derived neurotrophic factor improves blood glucose control and alleviates fasting hyperglycemia in C57BLKS-Lepr^db^/lepr^db^ mice. Diabetes.

[242] Nakagawa T., Tsuchida A., Itakura Y., Nonomura T., Ono M., Hirota F., Inoue T., Nakayama C., Taiji M., Noguchi H. (2000). Brain-derived neurotrophic factor regulates glucose metabolism by modulating energy balance in diabetic mice. Diabetes.

[243] Ono M., Ichihara J., Nonomura T., Itakura Y., Taiji M., Nakayama C., Noguchi H. (1997). Brain-derived neurotrophic factor reduces blood glucose level in obese diabetic mice but not in normal mice. Biochem. Biophys. Res. Commun..

[244] Rozanska O., Uruska A., Zozulinska-Ziolkiewicz D. (2020). Brain-derived neurotrophic factor and diabetes. Int. J. Mol. Sci..

[245] Kuroda A., Yamasaki Y., Matsuhisa M., Kubota M., Nakahara I., Nakatani Y., Hoshi A., Gorogawa S., Umayahara Y., Itakura Y. (2003). Brain-derived neurotrophic factor ameliorates hepatic insulin resistance in Zucker fatty rats. Metabolism.

[246] Meek T.H., Wisse B.E., Thaler J.P., Guyenet S.J., Matsen M.E., Fischer J.D., Taborsky G.J., Schwartz M.W., Morton G.J. (2013). BDNF action in the brain attenuates diabetic hyperglycemia via insulin-independent inhibition of hepatic glucose production. Diabetes.

[247] Fulgenzi G., Hong Z., Tomassoni-Ardori F., Barella L.F., Becker J., Barrick C., Swing D., Yanpallewar S., Croix B.S., Wess J. (2020). Novel metabolic role for BDNF in pancreatic β-cell insulin secretion. Nat. Commun..

[248] Sakhneny L., Rachi E., Epshtein A., Guez H.C., Wald-Altman S., Lisnyansky M., Khalifa-Malka L., Hazan A., Baer D., Priel A. (2018). Pancreatic pericytes support β-cell function in a Tcf7l2-dependent manner. Diabetes.

[249] Sakhneny L., Mueller L., Schonblum A., Azaria S., Burganova G., Epshtein A., Isaacson A., Wilson H., Spagnoli F.M., Landsman L. (2021). The postnatal pancreatic microenvironment guides β cell maturation through BMP4 production. Dev. Cell.

[250] Goulley J., Dahl U., Baeza N., Mishina Y., Edlund H. (2007). BMP4-BMPR1A signaling in β cells is required for and augments glucose-stimulated insulin secretion. Cell Metab..

[251] Sasson A., Rachi E., Sakhneny L., Baer D., Lisnyansky M., Epshtein A., Landsman L. (2016). Islet pericytes are required for β-cell maturity. Diabetes.

[252] Segerstolpe Å., Palasantza A., Eliasson P., Andersson E.M., Andréasson A.C., Sun X., Picelli S., Sabirsh A., Clausen M., Bjursell M.K. (2016). Single-cell transcriptome profiling of human pancreatic islets in health and type 2 diabetes. Cell Metab..

[253] Bruun C., Christensen G.L., Jacobsen M.L., Kanstrup M.B., Jensen P.R., Fjordvang H., Mandrup-Poulsen T., Billestrup N. (2014). Inhibition of β cell growth and function by bone morphogenetic proteins. Diabetologia.

[254] Boström K.I., Jumabay M., Matveyenko A., Nicholas S.B., Yao Y. (2011). Activation of vascular bone morphogenetic protein signaling in diabetes mellitus. Circ. Res..

[255] Koga M., Engberding N., Dikalova A.E., Chang K.H., Seidel-Rogol B., Long J.S., Lassègue B., Jo H., Griendling K.K. (2013). The bone morphogenic protein inhibitor, noggin, reduces glycemia and vascular inflammation in *db*/*db* mice. Am. J. Physiol. Heart Circ. Physiol..

[256] Sorescu G.P., Sykes M., Weiss D., Platt M.O., Saha A., Hwang J., Boyd N., Boo Y.C., Vega J.D., Taylor W.R. (2003). Bone morphogenic protein 4 produced in endothelial cells by oscillatory shear stress stimulates an inflammatory response. J. Biol. Chem..

[257] Nett P.C., Ortmann J., Celeiro J., Haas E., Hofmann-Lehmann R., Tornillo L., Terraciano L.M., Barton M. (2006). Transcriptional regulation of vascular bone morphogenetic protein by endothelin receptors in early autoimmune diabetes mellitus. Life Sci..

[258] Hua H., Zhang Y.Q., Dabernat S., Kritzik M., Dietz D., Sterling L., Sarvetnick N. (2006). BMP4 regulates pancreatic progenitor cell expansion through Id2. J. Biol. Chem..

[259] Sui L., Geens M., Sermon K., Bouwens L., Mfopou J.K. (2013). Role of BMP signaling in pancreatic progenitor differentiation from human embryonic stem cells. Stem Cell Rev. Rep..

[260] Chmielowiec J., Szlachcic W.J., Yang D., Scavuzzo M.A., Wamble K., Sarrion-Perdigones A., Sabek O.M., Venken K.J.T., Borowiak M. (2022). Human pancreatic microenvironment promotes β-cell differentiation via non-canonical WNT5A/JNK and BMP signaling. Nat. Commun..

[261] Chung W.S., Andersson O., Row R., Kimelman D., Stainier D.Y. (2010). Suppression of Alk8-mediated Bmp signaling cell-autonomously induces pancreatic β-cells in zebrafish. Proc. Natl. Acad. Sci. USA.

[262] Pauk M., Bordukalo-Niksic T., Brkljacic J., Paralkar V.M., Brault A.L., Dumic-Cule I., Borovecki F., Grgurevic L., Vukicevic S. (2019). A novel role of bone morphogenetic protein 6 (BMP6) in glucose homeostasis. Acta Diabetol..

[263] Tritschler S., Thomas M., Böttcher A., Ludwig B., Schmid J., Schubert U., Kemter E., Wolf E., Lickert H., Theis F.J. (2022). A transcriptional cross species map of pancreatic islet cells. Mol. Metab..

[264] Nica A.C., Ongen H., Irminger J.C., Bosco D., Berney T., Antonarakis S.E., Halban P.A., Dermitzakis E.T. (2013). Cell-type, allelic, and genetic signatures in the human pancreatic β cell transcriptome. Genome Res..

[265] Muraro M.J., Dharmadhikari G., Grün D., Groen N., Dielen T., Jansen E., van Gurp L., Engelse M.A., Carlotti F., de Koning E.J. (2016). A single-cell transcriptome atlas of the human pancreas. Cell Syst..

[266] Jiang F.X., Stanley E.G., Gonez L.J., Harrison L.C. (2002). Bone morphogenetic proteins promote development of fetal pancreas epithelial colonies containing insulin-positive cells. J. Cell Sci..

[267] Kim H.T., Desouza A.H., Umhoefer H., Han J., Anzia L., Sacotte S.J., Williams R.A., Blumer J.T., Bartosiak J.T., Fontaine D.A. (2022). Cholecystokinin attenuates β-cell apoptosis in both mouse and human islets. Transl. Res..

[268] Lavine J.A., Kibbe C.R., Baan M., Sirinvaravong S., Umhoefer H.M., Engler K.A., Meske L.M., Sacotte K.A., Erhardt D.P., Davis D.B. (2015). Cholecystokinin expression in the β-cell leads to increased β-cell area in aged mice and protects from streptozotocin-induced diabetes and apoptosis. Am. J. Physiol. Endocrinol. Metab..

[269] Linnemann A.K., Neuman J.C., Battiola T.J., Wisinski J.A., Kimple M.E., Davis D.B. (2015). Glucagon-like peptide-1 regulates cholecystokinin production in β-cells to protect from apoptosis. Mol. Endocrinol..

[270] Rezende L.F., Stoppiglia L.F., Souza K.L., Negro A., Langone F., Boschero A.C. (2007). Ciliary neurotrophic factor promotes survival of neonatal rat islets via the BCL-2 anti-apoptotic pathway. J. Endocrinol..

[271] Lemper M., De Groef S., Stangé G., Baeyens L., Heimberg H. (2016). A combination of cytokines EGF and CNTF protects the functional beta cell mass in mice with short-term hyperglycaemia. Diabetologia.

[272] Guney M.A., Petersen C.P., Boustani A., Duncan M.R., Gunasekaran U., Menon R., Warfield C., Grotendorst G.R., Means A.L., Economides A.N. (2011). Connective tissue growth factor acts within both endothelial cells and β cells to promote proliferation of developing β cells. Proc. Natl. Acad. Sci. USA.

[273] Crawford L.A., Guney M.A., Oh Y.A., Deyoung R.A., Valenzuela D.M., Murphy A.J., Yancopoulos G.D., Lyons K.M., Brigstock D.R., Economides A. (2009). Connective tissue growth factor (CTGF) inactivation leads to defects in islet cell lineage allocation and β-cell proliferation during embryogenesis. Mol. Endocrinol..

[274] Pasek R.C., Dunn J.C., Elsakr J.M., Aramandla M., Matta A.R., Gannon M. (2017). Vascular-derived connective tissue growth factor (Ctgf) is critical for pregnancy-induced β cell hyperplasia in adult mice. Islets.

[275] Riley K.G., Pasek R.C., Maulis M.F., Peek J., Thorel F., Brigstock D.R., Herrera P.L., Gannon M. (2015). Connective tissue growth factor modulates adult β-cell maturity and proliferation to promote β-cell regeneration in mice. Diabetes.

[276] Hakonen E., Ustinov J., Mathijs I., Palgi J., Bouwens L., Miettinen P.J., Otonkoski T. (2011). Epidermal growth factor (EGF)-receptor signalling is needed for murine β cell mass expansion in response to high-fat diet and pregnancy but not after pancreatic duct ligation. Diabetologia.

[277] Hakonen E., Ustinov J., Palgi J., Miettinen P.J., Otonkoski T. (2014). EGFR signaling promotes β-cell proliferation and survivin expression during pregnancy. PLoS ONE.

[278] Hanley S.C., Assouline-Thomas B., Makhlin J., Rosenberg L. (2011). Epidermal growth factor induces adult human islet cell dedifferentiation. J. Endocrinol..

[279] Zarrouki B., Benterki I., Fontés G., Peyot M.L., Seda O., Prentki M., Poitout V. (2014). Epidermal growth factor receptor signaling promotes pancreatic β-cell proliferation in response to nutrient excess in rats through mTOR and FOXM1. Diabetes.

[280] Kuntz E., Broca C., Komurasaki T., Kaltenbacher M.C., Gross R., Pinget M., Damgé C. (2005). Effect of epiregulin on pancreatic β cell growth and insulin secretion. Growth Factors.

[281] Song N.J., Lee A., Yasmeen R., Shen Q., Yang K., Kumar S.B., Muhanna D., Arnipalli S., Noria S.F., Needleman B.J. (2022). Epiregulin as an alternative ligand for leptin receptor alleviates glucose intolerance without change in obesity. Cells.

[282] Maachi H., Fergusson G., Ethier M., Brill G.N., Katz L.S., Honig L.B., Metukuri M.R., Scott D.K., Ghislain J., Poitout V. (2020). HB-EGF Signaling Is Required for Glucose-Induced Pancreatic β-Cell Proliferation in Rats. Diabetes.

[283] Mashima H., Ohnishi H., Wakabayashi K., Mine T., Miyagawa J., Hanafusa T., Seno M., Yamada H., Kojima I. (1996). Betacellulin and activin A coordinately convert amylase-secreting pancreatic AR42J cells into insulin-secreting cells. J. Clin. Investig..

[284] Li L., Seno M., Yamada H., Kojima I. (2003). Betacellulin improves glucose metabolism by promoting conversion of intraislet precursor cells to β-cells in streptozotocin-treated mice. Am. J. Physiol. Endocrinol. Metab..

[285] Yamamoto Y., Yamada S., Kodera T., Hara A., Motoyoshi K., Tanaka Y., Nagaoka T., Seno M., Kojima I. (2008). Reversal of streptozotocin-induced hyperglycemia by continuous supply of betacellulin in mice. Growth Factors.

[286] Oh Y.S., Shin S., Li H.Y., Park E.Y., Lee S.M., Choi C.S., Lim Y., Jung H.S., Jun H.S. (2015). Betacellulin ameliorates hyperglycemia in obese diabetic *db*/*db* mice. J. Mol. Med..

[287] Miettinen P., Ormio P., Hakonen E., Banerjee M., Otonkoski T. (2008). EGF receptor in pancreatic β-cell mass regulation. Biochem. Soc. Trans..

[288] Miettinen P.J., Ustinov J., Ormio P., Gao R., Palgi J., Hakonen E., Juntti-Berggren L., Berggren P.O., Otonkoski T. (2006). Downregulation of EGF receptor signaling in pancreatic islets causes diabetes due to impaired postnatal β-cell growth. Diabetes.

[289] Thowfeequ S., Ralphs K.L., Yu W.Y., Slack J.M., Tosh D. (2007). Betacellulin inhibits amylase and glucagon production and promotes β cell differentiation in mouse embryonic pancreas. Diabetologia.

[290] Huotari M.A., Palgi J., Otonkoski T. (1998). Growth factor-mediated proliferation and differentiation of insulin-producing INS-1 and RINm5F cells: Identification of betacellulin as a novel beta-cell mitogen. Endocrinology.

[291] Suarez-Pinzon W.L., Lakey J.R., Brand S.J., Rabinovitch A. (2005). Combination therapy with epidermal growth factor and gastrin induces neogenesis of human islet β-cells from pancreatic duct cells and an increase in functional β-cell mass. J. Clin. Endocrinol. Metab..

[292] Krakowski M.L., Kritzik M.R., Jones E.M., Krahl T., Lee J., Arnush M., Gu D., Mroczkowski B., Sarvetnick N. (1999). Transgenic expression of epidermal growth factor and keratinocyte growth factor in β-cells results in substantial morphological changes. J. Endocrinol..

[293] Song I., Patel O., Himpe E., Muller C.J., Bouwens L. (2015). β cell mass restoration in alloxan-diabetic mice treated with EGF and gastrin. PLoS ONE.

[294] Rooman I., Bouwens L. (2004). Combined gastrin and epidermal growth factor treatment induces islet regeneration and restores normoglycaemia in C57Bl6/J mice treated with alloxan. Diabetologia.

[295] Baeyens L., De Breuck S., Lardon J., Mfopou J.K., Rooman I., Bouwens L. (2005). In vitro generation of insulin-producing beta cells from adult exocrine pancreatic cells. Diabetologia.

[296] Lingohr M.K., Dickson L.M., McCuaig J.F., Hugl S.R., Twardzik D.R., Rhodes C.J. (2002). Activation of IRS-2-mediated signal transduction by IGF-1, but not TGF-alpha or EGF, augments pancreatic β-cell proliferation. Diabetes.

[297] Hart A.W., Baeza N., Apelqvist A., Edlund H. (2000). Attenuation of FGF signalling in mouse β-cells leads to diabetes. Nature.

[298] Wente W., Efanov A.M., Brenner M., Kharitonenkov A., Köster A., Sandusky G.E., Sewing S., Treinies I., Zitzer H., Gromada J. (2006). Fibroblast growth factor-21 improves pancreatic β-cell function and survival by activation of extracellular signal-regulated kinase 1/2 and Akt signaling pathways. Diabetes.

[299] Movassat J., Beattie G.M., Lopez A.D., Portha B., Hayek A. (2003). Keratinocyte growth factor and β-cell differentiation in human fetal pancreatic endocrine precursor cells. Diabetologia.

[300] Hebrok M., Kim S.K., Melton D.A. (1998). Notochord repression of endodermal Sonic hedgehog permits pancreas development. Genes Dev..

[301] Ndlovu R., Deng L.C., Wu J., Li X.K., Zhang J.S. (2018). Fibroblast growth factor 10 in pancreas development and pancreatic cancer. Front. Genet..

[302] Uzan B., Figeac F., Portha B., Movassat J. (2009). Mechanisms of KGF mediated signaling in pancreatic duct cell proliferation and differentiation. PLoS ONE.

[303] Wagner M., Koschnick S., Beilke S., Frey M., Adler G., Schmid R.M. (2008). Selective expansion of the β-cell compartment in the pancreas of keratinocyte growth factor transgenic mice. Am. J. Physiol. Gastrointest. Liver Physiol..

[304] Harmon E.B., Apelqvist A.A., Smart N.G., Gu X., Osborne D.H., Kim S.K. (2004). GDF11 modulates NGN3^+^ islet progenitor cell number and promotes β-cell differentiation in pancreas development. Development.

[305] Dichmann D.S., Yassin H., Serup P. (2006). Analysis of pancreatic endocrine development in GDF11-deficient mice. Dev. Dyn..

[306] Bootcov M.R., Bauskin A.R., Valenzuela S.M., Moore A.G., Bansal M., He X.Y., Zhang H.P., Donnellan M., Mahler S., Pryor K. (1997). MIC-1, a novel macrophage inhibitory cytokine, is a divergent member of the TGF-beta superfamily. Proc. Natl. Acad. Sci. USA.

[307] Carstensen M., Herder C., Brunner E.J., Strassburger K., Tabak A.G., Roden M., Witte D.R. (2010). Macrophage inhibitory cytokine-1 is increased in individuals before type 2 diabetes diagnosis but is not an independent predictor of type 2 diabetes: The Whitehall II study. Eur. J. Endocrinol..

[308] Chung H.K., Ryu D., Kim K.S., Chang J.Y., Kim Y.K., Yi H.S., Kang S.G., Choi M.J., Lee S.E., Jung S.B. (2017). Growth differentiation factor 15 is a myomitokine governing systemic energy homeostasis. J. Cell Biol..

[309] Jung S.B., Choi M.J., Ryu D., Yi H.S., Lee S.E., Chang J.Y., Chung H.K., Kim Y.K., Kang S.G., Lee J.H. (2018). Reduced oxidative capacity in macrophages results in systemic insulin resistance. Nat. Commun..

[310] Lee S.E., Kang S.G., Choi M.J., Jung S.B., Ryu M.J., Chung H.K., Chang J.Y., Kim Y.K., Lee J.H., Kim K.S. (2017). Growth differentiation factor 15 mediates systemic glucose regulatory action of T-helper type 2 cytokines. Diabetes.

[311] Kang Y.E., Kim H.J., Shong M. (2019). Regulation of systemic glucose homeostasis by T helper type 2 cytokines. Diabetes Metab. J..

[312] Al-Kuraishy H.M., Al-Gareeb A.I., Alexiou A., Papadakis M., Nadwa E.H., Albogami S.M., Alorabi M., Saad H.M., Batiha G.E. (2022). Metformin and growth differentiation factor 15 (GDF15) in type 2 diabetes mellitus: A hidden treasure. J. Diabetes.

[313] Vila G., Riedl M., Anderwald C., Resl M., Handisurya A., Clodi M., Prager G., Ludvik B., Krebs M., Luger A. (2011). The relationship between insulin resistance and the cardiovascular biomarker growth differentiation factor-15 in obese patients. Clin. Chem..

[314] Friedrichsen B.N., Galsgaard E.D., Nielsen J.H., Møldrup A. (2001). Growth hormone- and prolactin-induced proliferation of insulinoma cells, INS-1, depends on activation of STAT5 (signal transducer and activator of transcription 5). Mol. Endocrinol..

[315] Brelje T.C., Stout L.E., Bhagroo N.V., Sorenson R.L. (2004). Distinctive roles for prolactin and growth hormone in the activation of Signal transducer and activator of transcription 5 in pancreatic islets of Langerhans. Endocrinology.

[316] Huang Y., Chang Y. (2014). Regulation of pancreatic islet beta-cell mass by growth factor and hormone signaling. Prog. Mol. Biol. Transl. Sci..

[317] Ma F., Wei Z., Shi C., Gan Y., Lu J., Frank S.J., Balducci J., Huang Y. (2011). Signaling cross talk between growth hormone (GH) and insulin-like growth factor-I (IGF-I) in pancreatic islet β-cells. Mol. Endocrinol..

[318] Pacini G., Thomaseth K., Ahrén B. (2010). Dissociated effects of glucose-dependent insulinotropic polypeptide vs glucagon-like peptide-1 on β-cell secretion and insulin clearance in mice. Metabolism.

[319] Pacini G., Ahrén B. (2017). Glucagon-like peptide-1 and glucose-dependent insulinotropic peptide: Effects alone and in combination on insulin secretion and glucose disappearance in mice. Physiol. Rep..

[320] Campbell J.E., Drucker D.J. (2013). Pharmacology, physiology, and mechanisms of incretin hormone action. Cell Metab..

[321] Lavine J.A., Attie A.D. (2010). Gastrointestinal hormones and the regulation of β-cell mass. Ann. N. Y. Acad. Sci..

[322] McIntosh C.H., Widenmaier S., Kim S.J. (2009). Glucose-dependent insulinotropic polypeptide (Gastric Inhibitory Polypeptide; GIP). Vitam Horm.

[323] Meier J.J., Hücking K., Holst J.J., Deacon C.F., Schmiegel W.H., Nauck M.A. (2001). Reduced insulinotropic effect of gastric inhibitory polypeptide in first-degree relatives of patients with type 2 diabetes. Diabetes.

[324] Nauck M.A., Heimesaat M.M., Orskov C., Holst J.J., Ebert R., Creutzfeldt W. (1993). Preserved incretin activity of glucagon-like peptide 1 [7-36 amide] but not of synthetic human gastric inhibitory polypeptide in patients with type-2 diabetes mellitus. J. Clin. Investig..

[325] Seino Y., Fukushima M., Yabe D. (2010). GIP and GLP-1, the two incretin hormones: Similarities and differences. J. Diabetes Investig.

[326] Lyssenko V., Eliasson L., Kotova O., Pilgaard K., Wierup N., Salehi A., Wendt A., Jonsson A., De Marinis Y.Z., Berglund L.M. (2011). Pleiotropic effects of GIP on islet function involve osteopontin. Diabetes.

[327] Sandoval D.A., D’Alessio D.A. (2015). Physiology of proglucagon peptides: Role of glucagon and GLP-1 in health and disease. Physiol. Rev..

[328] Drucker D.J. (2006). The biology of incretin hormones. Cell Metab..

[329] Edholm T., Cejvan K., Abdel-Halim S.M., Efendic S., Schmidt P.T., Hellström P.M. (2009). The incretin hormones GIP and GLP-1 in diabetic rats: Effects on insulin secretion and small bowel motility. Neurogastroenterol. Motil..

[330] DeFronzo R.A., Ratner R.E., Han J., Kim D.D., Fineman M.S., Baron A.D. (2005). Effects of exenatide (exendin-4) on glycemic control and weight over 30 weeks in metformin-treated patients with type 2 diabetes. Diabetes Care.

[331] Movassat J., Beattie G.M., Lopez A.D., Hayek A. (2002). Exendin 4 up-regulates expression of PDX 1 and hastens differentiation and maturation of human fetal pancreatic cells. J. Clin. Endocrinol. Metab..

[332] Farnsworth N.L., Walter R., Piscopio R.A., Schleicher W.E., Benninger R.K.P. (2019). Exendin-4 overcomes cytokine-induced decreases in gap junction coupling via protein kinase A and Epac2 in mouse and human islets. J. Physiol..

[333] Perfetti R., Zhou J., Doyle M.E., Egan J.M. (2000). Glucagon-like peptide-1 induces cell proliferation and pancreatic-duodenum homeobox-1 expression and increases endocrine cell mass in the pancreas of old, glucose-intolerant rats. Endocrinology.

[334] Tourrel C., Bailbé D., Meile M.J., Kergoat M., Portha B. (2001). Glucagon-like peptide-1 and exendin-4 stimulate β-cell neogenesis in streptozotocin-treated newborn rats resulting in persistently improved glucose homeostasis at adult age. Diabetes.

[335] Parkes D.G., Pittner R., Jodka C., Smith P., Young A. (2001). Insulinotropic actions of exendin-4 and glucagon-like peptide-1 in vivo and in vitro. Metabolism.

[336] Mashima H., Shibata H., Mine T., Kojima I. (1996). Formation of insulin-producing cells from pancreatic acinar AR42J cells by hepatocyte growth factor. Endocrinology.

[337] Fiaschi-Taesch N., Stewart A.F., Garcia-Ocaña A. (2007). Improving islet transplantation by gene delivery of hepatocyte growth factor (HGF) and its downstream target, protein kinase B (PKB)/Akt. Cell Biochem. Biophys..

[338] Otonkoski T., Cirulli V., Beattie M., Mally M.I., Soto G., Rubin J.S., Hayek A. (1996). A role for hepatocyte growth factor/scatter factor in fetal mesenchyme-induced pancreatic β-cell growth. Endocrinology.

[339] Otonkoski T., Beattie G.M., Rubin J.S., Lopez A.D., Baird A., Hayek A. (1994). Hepatocyte growth factor/scatter factor has insulinotropic activity in human fetal pancreatic cells. Diabetes.

[340] Beattie G.M., Montgomery A.M., Lopez A.D., Hao E., Perez B., Just M.L., Lakey J.R., Hart M.E., Hayek A. (2002). A novel approach to increase human islet cell mass while preserving β-cell function. Diabetes.

[341] Hayek A., Beattie G.M., Cirulli V., Lopez A.D., Ricordi C., Rubin J.S. (1995). Growth factor/matrix-induced proliferation of human adult β-cells. Diabetes.

[342] Garcia-Ocaña A., Takane K.K., Syed M.A., Philbrick W.M., Vasavada R.C., Stewart A.F. (2000). Hepatocyte growth factor overexpression in the islet of transgenic mice increases β cell proliferation, enhances islet mass, and induces mild hypoglycemia. J. Biol. Chem..

[343] García-Ocaña A., Vasavada R.C., Cebrian A., Reddy V., Takane K.K., López-Talavera J.C., Stewart A.F. (2001). Transgenic overexpression of hepatocyte growth factor in the beta-cell markedly improves islet function and islet transplant outcomes in mice. Diabetes.

[344] Garcia-Ocaña A., Takane K.K., Reddy V.T., Lopez-Talavera J.C., Vasavada R.C., Stewart A.F. (2003). Adenovirus-mediated hepatocyte growth factor expression in mouse islets improves pancreatic islet transplant performance and reduces beta cell death. J. Biol. Chem..

[345] Fiaschi-Taesch N.M., Berman D.M., Sicari B.M., Takane K.K., Garcia-Ocaña A., Ricordi C., Kenyon N.S., Stewart A.F. (2008). Hepatocyte growth factor enhances engraftment and function of nonhuman primate islets. Diabetes.

[346] Gahr S., Merger M., Bollheimer L.C., Hammerschmied C.G., Schölmerich J., Hügl S.R. (2002). Hepatocyte growth factor stimulates proliferation of pancreatic β-cells particularly in the presence of subphysiological glucose concentrations. J. Mol. Endocrinol..

[347] Lopez-Talavera J.C., Garcia-Ocaña A., Sipula I., Takane K.K., Cozar-Castellano I., Stewart A.F. (2004). Hepatocyte growth factor gene therapy for pancreatic islets in diabetes: Reducing the minimal islet transplant mass required in a glucocorticoid-free rat model of allogeneic portal vein islet transplantation. Endocrinology.

[348] Yeung T.Y., Seeberger K.L., Kin T., Adesida A., Jomha N., Shapiro A.M., Korbutt G.S. (2012). Human mesenchymal stem cells protect human islets from pro-inflammatory cytokines. PLoS ONE.

[349] Dai C., Li Y., Yang J., Liu Y. (2003). Hepatocyte growth factor preserves β cell mass and mitigates hyperglycemia in streptozotocin-induced diabetic mice. J. Biol. Chem..

[350] Stewart A.F., Hussain M.A., García-Ocaña A., Vasavada R.C., Bhushan A., Bernal-Mizrachi E., Kulkarni R.N. (2015). Human β-cell proliferation and intracellular signaling: Part 3. Diabetes.

[351] Agudo J., Ayuso E., Jimenez V., Salavert A., Casellas A., Tafuro S., Haurigot V., Ruberte J., Segovia J.C., Bueren J. (2008). IGF-I mediates regeneration of endocrine pancreas by increasing beta cell replication through cell cycle protein modulation in mice. Diabetologia.

[352] Kulkarni R.N., Holzenberger M., Shih D.Q., Ozcan U., Stoffel M., Magnuson M.A., Kahn C.R. (2002). β-cell-specific deletion of the Igf1 receptor leads to hyperinsulinemia and glucose intolerance but does not alter β-cell mass. Nat. Genet..

[353] Taylor-Fishwick D.A., Bowman A., Hamblet N., Bernard P., Harlan D.M., Vinik A.I. (2006). Islet neogenesis associated protein transgenic mice are resistant to hyperglycemia induced by streptozotocin. J. Endocrinol..

[354] Pittenger G.L., Taylor-Fishwick D., Vinik A.I. (2009). The role of islet neogeneis-associated protein (INGAP) in pancreatic islet neogenesis. Curr. Protein Pept. Sci..

[355] Lipsett M., Hanley S., Castellarin M., Austin E., Suarez-Pinzon W.L., Rabinovitch A., Rosenberg L. (2007). The role of islet neogenesis-associated protein (INGAP) in islet neogenesis. Cell Biochem. Biophys..

[356] South J.C.M., Blackburn E., Brown I.R., Gullick W.J. (2013). The neuregulin system of ligands and their receptors in rat islets of Langerhans. Endocrinology.

[357] Liu Y., Chen M. (2022). Neuregulin 4 as a novel adipokine in energy metabolism. Front. Physiol..

[358] Katakam A.K., Chipitsyna G., Gong Q., Vancha A.R., Gabbeta J., Arafat H.A. (2005). Streptozotocin (STZ) mediates acute upregulation of serum and pancreatic osteopontin (OPN): A novel islet-protective effect of OPN through inhibition of STZ-induced nitric oxide production. J. Endocrinol..

[359] Cai M., Bompada P., Salehi A., Acosta J.R., Prasad R.B., Atac D., Laakso M., Groop L., De Marinis Y. (2018). Role of osteopontin and its regulation in pancreatic islet. Biochem. Biophys. Res. Commun..

[360] Liu X., Zhang F., Chai Y., Wang L., Yu B. (2020). The role of bone-derived PDGF-AA in age-related pancreatic β cell proliferation and function. Biochem. Biophys. Res. Commun..

[361] Chen H., Gu X., Liu Y., Wang J., Wirt S.E., Bottino R., Schorle H., Sage J., Kim S.K. (2011). PDGF signalling controls age-dependent proliferation in pancreatic β-cells. Nature.

[362] Yang W., Jiang Y., Wang Y., Zhang T., Liu Q., Wang C., Swisher G., Wu N., Chao C., Prasadan K. (2020). Placental growth factor in β cells plays an essential role in gestational β-cell growth. BMJ Open Diabetes Res. Care.

[363] Vasavada R.C., Garcia-Ocaña A., Zawalich W.S., Sorenson R.L., Dann P., Syed M., Ogren L., Talamantes F., Stewart A.F. (2000). Targeted expression of placental lactogen in the βcells of transgenic mice results in β cell proliferation, islet mass augmentation, and hypoglycemia. J. Biol. Chem..

[364] Hügl S.R., Merger M. (2007). Prolactin stimulates proliferation of the glucose-dependent beta-cell line INS-1 via different IRS-proteins. Jop.

[365] Xu Y., Wang X., Gao L., Zhu J., Zhang H., Shi H., Woo M., Wu X. (2015). Prolactin-stimulated survivin induction is required for β cell mass expansion during pregnancy in mice. Diabetologia.

[366] Zhang B., Hosaka M., Sawada Y., Torii S., Mizutani S., Ogata M., Izumi T., Takeuchi T. (2003). Parathyroid hormone-related protein induces insulin expression through activation of MAP kinase-specific phosphatase-1 that dephosphorylates c-Jun NH2-terminal kinase in pancreatic β-cells. Diabetes.

[367] Villanueva-Peñacarrillo M.L., Cancelas J., de Miguel F., Redondo A., Valín A., Valverde I., Esbrit P. (1999). Parathyroid hormone-related peptide stimulates DNA synthesis and insulin secretion in pancreatic islets. J. Endocrinol..

[368] Guthalu Kondegowda N., Joshi-Gokhale S., Harb G., Williams K., Zhang X.Y., Takane K.K., Zhang P., Scott D.K., Stewart A.F., Garcia-Ocaña A. (2010). Parathyroid hormone-related protein enhances human β-cell proliferation and function with associated induction of cyclin-dependent kinase 2 and cyclin E expression. Diabetes.

[369] Williams K., Abanquah D., Joshi-Gokhale S., Otero A., Lin H., Guthalu N.K., Zhang X., Mozar A., Bisello A., Stewart A.F. (2011). Systemic and acute administration of parathyroid hormone-related peptide (1-36) stimulates endogenous β cell proliferation while preserving function in adult mice. Diabetologia.

[370] Mozar A., Lin H., Williams K., Chin C., Li R., Kondegowda N.G., Stewart A.F., Garcia-Ocaña A., Vasavada R.C. (2016). Parathyroid hormone-related peptide (1-36) enhances β cell regeneration and increases beta cell mass in a mouse model of partial pancreatectomy. PLoS ONE.

[371] Alagpulinsa D.A., Cao J.J.L., Sobell D., Poznansky M.C. (2019). Harnessing CXCL12 signaling to protect and preserve functional β-cell mass and for cell replacement in type 1 diabetes. Pharmacol. Ther..

[372] Alagpulinsa D.A., Cao J.J.L., Driscoll R.K., Sîrbulescu R.F., Penson M.F.E., Sremac M., Engquist E.N., Brauns T.A., Markmann J.F., Melton D.A. (2019). Alginate-microencapsulation of human stem cell-derived β cells with CXCL12 prolongs their survival and function in immunocompetent mice without systemic immunosuppression. Am. J. Transplant..

[373] Cunha D.A., Cito M., Carlsson P.O., Vanderwinden J.M., Molkentin J.D., Bugliani M., Marchetti P., Eizirik D.L., Cnop M. (2016). Thrombospondin 1 protects pancreatic β-cells from lipotoxicity via the PERK-NRF2 pathway. Cell Death Differ..

[374] Olerud J., Mokhtari D., Johansson M., Christoffersson G., Lawler J., Welsh N., Carlsson P.O. (2011). Thrombospondin-1: An islet endothelial cell signal of importance for β-cell function. Diabetes.

[375] Crawford S.E., Stellmach V., Murphy-Ullrich J.E., Ribeiro S.M., Lawler J., Hynes R.O., Boivin G.P., Bouck N. (1998). Thrombospondin-1 is a major activator of TGF-β1 in vivo. Cell.

[376] Reinert R.B., Cai Q., Hong J.Y., Plank J.L., Aamodt K., Prasad N., Aramandla R., Dai C., Levy S.E., Pozzi A. (2014). Vascular endothelial growth factor coordinates islet innervation via vascular scaffolding. Development.

[377] Cross S.E., Richards S.K., Clark A., Benest A.V., Bates D.O., Mathieson P.W., Johnson P.R., Harper S.J., Smith R.M. (2007). Vascular endothelial growth factor as a survival factor for human islets: Effect of immunosuppressive drugs. Diabetologia.

[378] Xiong Y., Scerbo M.J., Seelig A., Volta F., O’Brien N., Dicker A., Padula D., Lickert H., Gerdes J.M., Berggren P.O. (2020). Islet vascularization is regulated by primary endothelial cilia via VEGF-A-dependent signaling. eLife.

[379] Park K.S., Kim Y.S., Kim J.H., Choi B., Kim S.H., Tan A.H., Lee M.S., Lee M.K., Kwon C.H., Joh J.W. (2010). Trophic molecules derived from human mesenchymal stem cells enhance survival, function, and angiogenesis of isolated islets after transplantation. Transplantation.

[380] Cheng Y., Liu Y.F., Zhang J.L., Li T.M., Zhao N. (2007). Elevation of vascular endothelial growth factor production and its effect on revascularization and function of graft islets in diabetic rats. World J. Gastroenterol..

[381] Levitt H.E., Cyphert T.J., Pascoe J.L., Hollern D.A., Abraham N., Lundell R.J., Rosa T., Romano L.C., Zou B., O’Donnell C.P. (2011). Glucose stimulates human β cell replication in vivo in islets transplanted into NOD-severe combined immunodeficiency (SCID) mice. Diabetologia.

[382] Schmidt S.F., Madsen J.G., Frafjord K.Ø., Poulsen L.l., Salö S., Boergesen M., Loft A., Larsen B.D., Madsen M.S., Holst J.J. (2016). Integrative genomics outlines a biphasic glucose response and a ChREBP-RORγ axis regulating proliferation in β cells. Cell Rep..

[383] Metukuri M.R., Zhang P., Basantani M.K., Chin C., Stamateris R.E., Alonso L.C., Takane K.K., Gramignoli R., Strom S.C., O’Doherty R.M. (2012). ChREBP mediates glucose-stimulated pancreatic β-cell proliferation. Diabetes.

[384] Furth-Lavi J., Hija A., Tornovsky-Babeay S., Mazouz A., Dahan T., Stolovich-Rain M., Klochendler A., Dor Y., Avrahami D., Glaser B. (2022). Glycemic control releases regenerative potential of pancreatic β cells blocked by severe hyperglycemia. Cell Rep..

[385] Brun T., Li N., Jourdain A.A., Gaudet P., Duhamel D., Meyer J., Bosco D., Maechler P. (2015). Diabetogenic milieus induce specific changes in mitochondrial transcriptome and differentiation of human pancreatic islets. Hum. Mol. Genet..

[386] Robertson R.P. (2004). Chronic oxidative stress as a central mechanism for glucose toxicity in pancreatic islet β cells in diabetes. J. Biol. Chem..

[387] Reich E., Tamary A., Sionov R.V., Melloul D. (2012). Involvement of thioredoxin-interacting protein (TXNIP) in glucocorticoid-mediated β cell death. Diabetologia.

[388] Poungvarin N., Lee J.K., Yechoor V.K., Li M.V., Assavapokee T., Suksaranjit P., Thepsongwajja J.J., Saha P.K., Oka K., Chan L. (2012). Carbohydrate response element-binding protein (ChREBP) plays a pivotal role in beta cell glucotoxicity. Diabetologia.

[389] Assmann A., Ueki K., Winnay J.N., Kadowaki T., Kulkarni R.N. (2009). Glucose effects on beta-cell growth and survival require activation of insulin receptors and insulin receptor substrate 2. Mol. Cell. Biol..

[390] Zakaria A., Berthault C., Cosson B., Jung V., Guerrera I.C., Rachdi L., Scharfmann R. (2021). Glucose treatment of human pancreatic β-cells enhances translation of mRNAs involved in energetics and insulin secretion. J. Biol. Chem..

[391] Choi E.H., Park S.J. (2023). TXNIP: A key protein in the cellular stress response pathway and a potential therapeutic target. Exp. Mol. Med..

[392] Richards P., Rachdi L., Oshima M., Marchetti P., Bugliani M., Armanet M., Postic C., Guilmeau S., Scharfmann R. (2018). MondoA is an essential glucose-responsive transcription factor in human pancreatic β-cells. Diabetes.

[393] Bulfoni M., Bouyioukos C., Zakaria A., Nigon F., Rapone R., Del Maestro L., Ait-Si-Ali S., Scharfmann R., Cosson B. (2022). Glucose controls co-translation of structurally related mRNAs via the mTOR and eIF2 pathways in human pancreatic β cells. Front. Endocrinol..

[394] Ebrahim N., Shakirova K., Dashinimaev E. (2022). PDX1 is the cornerstone of pancreatic β-cell functions and identity. Front. Mol. Biosci..

[395] Melloul D. (2004). Transcription factors in islet development and physiology: Role of PDX-1 in β-cell function. Ann. N. Y. Acad. Sci..

[396] Marshak S., Benshushan E., Shoshkes M., Leibovitz G., Kaiser N., Gross D., Bertuzzi F., Cerasi E., Melloul D. (2001). β-cell-specific expression of insulin and PDX-1 genes. Diabetes.

[397] Gipson G.R., Nolan K., Kattamuri C., Kenny A.P., Agricola Z., Edwards N.A., Zinski J., Czepnik M., Mullins M.C., Zorn A.M. (2023). Formation and characterization of BMP2/GDF5 and BMP4/GDF5 heterodimers. BMC Biol..

[398] Mankoo B.S., Skuntz S., Harrigan I., Grigorieva E., Candia A., Wright C.V., Arnheiter H., Pachnis V. (2003). The concerted action of Meox homeobox genes is required upstream of genetic pathways essential for the formation, patterning and differentiation of somites. Development.

[399] Reijntjes S., Stricker S., Mankoo B.S. (2007). A comparative analysis of Meox1 and Meox2 in the developing somites and limbs of the chick embryo. Int. J. Dev. Biol..

[400] Quinn L.M., Latham S.E., Kalionis B. (2000). The homeobox genes MSX2 and MOX2 are candidates for regulating epithelial-mesenchymal cell interactions in the human placenta. Placenta.

[401] Mizusawa N., Hasegawa T., Ohigashi I., Tanaka-Kosugi C., Harada N., Itakura M., Yoshimoto K. (2004). Differentiation phenotypes of pancreatic islet β- and α-cells are closely related with homeotic genes and a group of differentially expressed genes. Gene.

[402] Wang J., Webb G., Cao Y., Steiner D.F. (2003). Contrasting patterns of expression of transcription factors in pancreatic α and β cells. Proc. Natl. Acad. Sci. USA.

[403] Nombela-Arrieta C., Ritz J., Silberstein L.E. (2011). The elusive nature and function of mesenchymal stem cells. Nat. Rev. Mol. Cell. Biol..

[404] Tavakoli S., Ghaderi Jafarbeigloo H.R., Shariati A., Jahangiryan A., Jadidi F., Jadidi Kouhbanani M.A., Hassanzadeh A., Zamani M., Javidi K., Naimi A. (2020). Mesenchymal stromal cells; a new horizon in regenerative medicine. J. Cell Physiol..

[405] Dominici M., Le Blanc K., Mueller I., Slaper-Cortenbach I., Marini F., Krause D., Deans R., Keating A., Prockop D., Horwitz E. (2006). Minimal criteria for defining multipotent mesenchymal stromal cells. The International Society for Cellular Therapy position statement. Cytotherapy.

[406] Boxall S.A., Jones E. (2012). Markers for characterization of bone marrow multipotential stromal cells. Stem Cells Int..

[407] Lv F.J., Tuan R.S., Cheung K.M., Leung V.Y. (2014). Concise review: The surface markers and identity of human mesenchymal stem cells. Stem Cells.

[408] Saalbach A., Anderegg U. (2019). Thy-1: More than a marker for mesenchymal stromal cells. FASEB J..

[409] Di Nicola M., Carlo-Stella C., Magni M., Milanesi M., Longoni P.D., Matteucci P., Grisanti S., Gianni A.M. (2002). Human bone marrow stromal cells suppress T-lymphocyte proliferation induced by cellular or nonspecific mitogenic stimuli. Blood.

[410] Aggarwal S., Pittenger M.F. (2005). Human mesenchymal stem cells modulate allogeneic immune cell responses. Blood.

[411] Majumdar M.K., Keane-Moore M., Buyaner D., Hardy W.B., Moorman M.A., McIntosh K.R., Mosca J.D. (2003). Characterization and functionality of cell surface molecules on human mesenchymal stem cells. J. Biomed. Sci..

[412] Tse W.T., Pendleton J.D., Beyer W.M., Egalka M.C., Guinan E.C. (2003). Suppression of allogeneic T-cell proliferation by human marrow stromal cells: Implications in transplantation. Transplantation.

[413] Műzes G., Sipos F. (2022). Mesenchymal stem cell-derived secretome: A potential therapeutic option for autoimmune and immune-mediated inflammatory diseases. Cells.

[414] Maitra B., Szekely E., Gjini K., Laughlin M.J., Dennis J., Haynesworth S.E., Koç O.N. (2004). Human mesenchymal stem cells support unrelated donor hematopoietic stem cells and suppress T-cell activation. Bone Marrow Transplant..

[415] Aksu A.E., Horibe E., Sacks J., Ikeguchi R., Breitinger J., Scozio M., Unadkat J., Feili-Hariri M. (2008). Co-infusion of donor bone marrow with host mesenchymal stem cells treats GVHD and promotes vascularized skin allograft survival in rats. Clin. Immunol..

[416] Laranjeira P., Pedrosa M., Pedreiro S., Gomes J., Martinho A., Antunes B., Ribeiro T., Santos F., Trindade H., Paiva A. (2015). Effect of human bone marrow mesenchymal stromal cells on cytokine production by peripheral blood naive, memory, and effector T cells. Stem Cell Res. Ther..

[417] Ben-Ami E., Berrih-Aknin S., Miller A. (2011). Mesenchymal stem cells as an immunomodulatory therapeutic strategy for autoimmune diseases. Autoimmun. Rev..

[418] Wang M., Yuan Q., Xie L. (2018). Mesenchymal stem cell-based immunomodulation: Properties and clinical application. Stem Cells Int..

[419] Wu X., Jiang J., Gu Z., Zhang J., Chen Y., Liu X. (2020). Mesenchymal stromal cell therapies: Immunomodulatory properties and clinical progress. Stem Cell Res. Ther..

[420] Wang Y., Fang J., Liu B., Shao C., Shi Y. (2022). Reciprocal regulation of mesenchymal stem cells and immune responses. Cell Stem Cell.

[421] Sotiropoulou P.A., Perez S.A., Gritzapis A.D., Baxevanis C.N., Papamichail M. (2006). Interactions between human mesenchymal stem cells and natural killer cells. Stem Cells.

[422] Lu D., Xu Y., Liu Q., Zhang Q. (2021). Mesenchymal stem cell-macrophage crosstalk and maintenance of inflammatory microenvironment homeostasis. Front. Cell Dev. Biol..

[423] Zheng G., Ge M., Qiu G., Shu Q., Xu J. (2015). Mesenchymal stromal cells affect disease outcomes via macrophage polarization. Stem Cells Int..

[424] Mrahleh M.A., Matar S., Jafar H., Wehaibi S., Aslam N., Awidi A. (2021). Human Wharton’s jelly-derived mesenchymal stromal cells primed by tumor necrosis factor-α and interferon-γ modulate the innate and adaptive immune cells of type 1 diabetic patients. Front. Immunol..

[425] Favaro E., Carpanetto A., Caorsi C., Giovarelli M., Angelini C., Cavallo-Perin P., Tetta C., Camussi G., Zanone M.M. (2016). Human mesenchymal stem cells and derived extracellular vesicles induce regulatory dendritic cells in type 1 diabetic patients. Diabetologia.

[426] Dokić J., Tomić S., Marković M., Milosavljević P., Colić M. (2013). Mesenchymal stem cells from periapical lesions modulate differentiation and functional properties of monocyte-derived dendritic cells. Eur. J. Immunol..

[427] Djouad F., Charbonnier L.-M., Bouffi C., Louis-Plence P., Bony C., Apparailly F., Cantos C., Jorgensen C., Noël D. (2007). Mesenchymal stem cells inhibit the differentiation of dendritic cells through an interleukin-6-dependent mechanism. Stem Cells.

[428] Nauta A.J., Kruisselbrink A.B., Lurvink E., Willemze R., Fibbe W.E. (2006). Mesenchymal stem cells inhibit generation and function of both CD34^+^-derived and monocyte-derived dendritic cells. J. Immunol..

[429] Jiang X.X., Zhang Y., Liu B., Zhang S.X., Wu Y., Yu X.D., Mao N. (2005). Human mesenchymal stem cells inhibit differentiation and function of monocyte-derived dendritic cells. Blood.

[430] Jiang D., Muschhammer J., Qi Y., Kügler A., de Vries J.C., Saffarzadeh M., Sindrilaru A., Beken S.V., Wlaschek M., Kluth M.A. (2016). Suppression of neutrophil-mediated tissue damage-A novel skill of mesenchymal stem cells. Stem Cells.

[431] Salami F., Tavassoli A., Mehrzad J., Parham A. (2018). Immunomodulatory effects of mesenchymal stem cells on leukocytes with emphasis on neutrophils. Immunobiology.

[432] Brandau S., Jakob M., Bruderek K., Bootz F., Giebel B., Radtke S., Mauel K., Jäger M., Flohé S.B., Lang S. (2014). Mesenchymal stem cells augment the anti-bacterial activity of neutrophil granulocytes. PLoS ONE.

[433] Prockop D.J. (1997). Marrow stromal cells as stem cells for nonhematopoietic tissues. Science.

[434] Caplan A.I. (2017). Mesenchymal stem cells: Time to change the name!. Stem Cells Transl. Med..

[435] Chen Q., Shou P., Zheng C., Jiang M., Cao G., Yang Q., Cao J., Xie N., Velletri T., Zhang X. (2016). Fate decision of mesenchymal stem cells: Adipocytes or osteoblasts?. Cell Death Differ..

[436] Crisan M., Yap S., Casteilla L., Chen C.W., Corselli M., Park T.S., Andriolo G., Sun B., Zheng B., Zhang L. (2008). A perivascular origin for mesenchymal stem cells in multiple human organs. Cell Stem Cell.

[437] Karnieli O., Izhar-Prato Y., Bulvik S., Efrat S. (2007). Generation of insulin-producing cells from human bone marrow mesenchymal stem cells by genetic manipulation. Stem Cells.

[438] Yianni V., Sharpe P.T. (2019). Perivascular-derived mesenchymal stem cells. J. Dent. Res..

[439] Tan L., Liu X., Dou H., Hou Y. (2022). Characteristics and regulation of mesenchymal stem cell plasticity by the microenvironment—Specific factors involved in the regulation of MSC plasticity. Genes Dis..

[440] Refaie A.F., Elbassiouny B.L., Kloc M., Sabek O.M., Khater S.M., Ismail A.M., Mohamed R.H., Ghoneim M.A. (2021). From mesenchymal stromal/stem cells to insulin-producing cells: Immunological considerations. Front. Immunol..

[441] Xie Q.P., Huang H., Xu B., Dong X., Gao S.L., Zhang B., Wu Y.L. (2009). Human bone marrow mesenchymal stem cells differentiate into insulin-producing cells upon microenvironmental manipulation in vitro. Differentiation.

[442] Ghoneim M.A., Refaie A.F., Elbassiouny B.L., Gabr M.M., Zakaria M.M. (2020). From mesenchymal stromal/stem cells to insulin-producing cells: Progress and challenges. Stem Cell Rev. Rep..

[443] Gabr M.M., Zakaria M.M., Refaie A.F., Ismail A.M., Abou-El-Mahasen M.A., Ashamallah S.A., Khater S.M., El-Halawani S.M., Ibrahim R.Y., Uin G.S. (2013). Insulin-producing cells from adult human bone marrow mesenchymal stem cells control streptozotocin-induced diabetes in nude mice. Cell Transplant..

[444] Gabr M.M., Sobh M.M., Zakaria M.M., Refaie A.F., Ghoneim M.A. (2008). Transplantation of insulin-producing clusters derived from adult bone marrow stem cells to treat diabetes in rats. Exp. Clin. Transplant..

[445] Limbert C., Päth G., Ebert R., Rothhammer V., Kassem M., Jakob F., Seufert J. (2011). PDX1- and NGN3-mediated in vitro reprogramming of human bone marrow-derived mesenchymal stromal cells into pancreatic endocrine lineages. Cytotherapy.

[446] Wang H., Yang Y., Ho G., Lin X., Wu W., Li W., Lin L., Feng X., Huo X., Jiang J. (2013). Programming of human umbilical cord mesenchymal stem cells in vitro to promote pancreatic gene expression. Mol. Med. Rep..

[447] Bhonde R.R., Sheshadri P., Sharma S., Kumar A. (2014). Making surrogate β-cells from mesenchymal stromal cells: Perspectives and future endeavors. Int. J. Biochem. Cell Biol..

[448] Ouyang J., Huang W., Yu W., Xiong W., Mula R.V., Zou H., Yu Y. (2014). Generation of insulin-producing cells from rat mesenchymal stem cells using an aminopyrrole derivative XW4.4. Chem. Biol. Interact..

[449] Sun Y., Chen L., Hou X.G., Hou W.K., Dong J.J., Sun L., Tang K.X., Wang B., Song J., Li H. (2007). Differentiation of bone marrow-derived mesenchymal stem cells from diabetic patients into insulin-producing cells in vitro. Chin. Med. J..

[450] Chang C.F., Hsu K.H., Chiou S.H., Ho L.L., Fu Y.S., Hung S.C. (2008). Fibronectin and pellet suspension culture promote differentiation of human mesenchymal stem cells into insulin producing cells. J. Biomed. Mater. Res. A.

[451] Ghoneim M.A., Gabr M.M., Refaie A.F., El-Halawani S.M., Al-Issawi M.M., Elbassiouny B.L., Kader M., Ismail A.M., Zidan M.F., Karras M.S. (2022). Transplantation of insulin-producing cells derived from human mesenchymal stromal/stem cells into diabetic humanized mice. Stem Cell Res. Ther..

[452] Moshtagh P.R., Emami S.H., Sharifi A.M. (2013). Differentiation of human adipose-derived mesenchymal stem cell into insulin-producing cells: An in vitro study. J. Physiol. Biochem.

[453] Kassem D.H., Kamal M.M., El-Kholy Ael L., El-Mesallamy H.O. (2016). Exendin-4 enhances the differentiation of Wharton’s jelly mesenchymal stem cells into insulin-producing cells through activation of various β-cell markers. Stem Cell Res. Ther..

[454] El-Asfar R.K., Kamal M.M., Abd El-Razek R.S., El-Demerdash E., El-Mesallamy H.O. (2018). Obestatin can potentially differentiate Wharton’s jelly mesenchymal stem cells into insulin-producing cells. Cell Tissue Res..

[455] Chandra V., Swetha G., Muthyala S., Jaiswal A.K., Bellare J.R., Nair P.D., Bhonde R.R. (2011). Islet-like cell aggregates generated from human adipose tissue derived stem cells ameliorate experimental diabetes in mice. PLoS ONE.

[456] Khorsandi L., Nejad-Dehbashi F., Ahangarpour A., Hashemitabar M. (2015). Three-dimensional differentiation of bone marrow-derived mesenchymal stem cells into insulin-producing cells. Tissue Cell.

[457] Daryabor G., Shiri E.H., Kamali-Sarvestani E. (2019). A simple method for the generation of insulin producing cells from bone marrow mesenchymal stem cells. In Vitro Cell. Dev. Biol. Anim..

[458] Xin Y., Jiang X., Wang Y., Su X., Sun M., Zhang L., Tan Y., Wintergerst K.A., Li Y., Li Y. (2016). Insulin-producing cells differentiated from human bone marrow mesenchymal stem cells in vitro ameliorate streptozotocin-induced diabetic hyperglycemia. PLoS ONE.

[459] Lee S.A., Kim S., Kim S.Y., Park J.Y., Nan J., Park H.S., Lee H., Lee Y.D., Lee H., Kang S. (2023). Direct differentiation of bone marrow mononucleated cells into insulin-producing cells using 4 specific soluble factors. Stem Cells Transl. Med..

[460] Liu S.H., Lee L.T. (2012). Efficient differentiation of mouse embryonic stem cells into insulin-producing cells. Exp. Diabetes Res..

[461] Ye D.Z., Tai M.H., Linning K.D., Szabo C., Olson L.K. (2006). MafA expression and insulin promoter activity are induced by nicotinamide and related compounds in INS-1 pancreatic β-cells. Diabetes.

[462] Silva I.B.B., Kimura C.H., Colantoni V.P., Sogayar M.C. (2022). Stem cells differentiation into insulin-producing cells (IPCs): Recent advances and current challenges. Stem Cell Res. Ther..

[463] Scuteri A., Donzelli E., Rodriguez-Menendez V., Ravasi M., Monfrini M., Bonandrini B., Figliuzzi M., Remuzzi A., Tredici G. (2014). A double mechanism for the mesenchymal stem cells’ positive effect on pancreatic islets. PLoS ONE.

[464] Di Vincenzo M., Martino M., Lariccia V., Giancola G., Licini C., Di Benedetto G., Arnaldi G., Orciani M. (2022). Mesenchymal stem cells exposed to persistently high glucocorticoid levels develop insulin-resistance and altered lipolysis: A promising in vitro model to study cushing’s syndrome. Front. Endocrinol..

[465] Navarro-Tableros V., Gai C., Gomez Y., Giunti S., Pasquino C., Deregibus M.C., Tapparo M., Pitino A., Tetta C., Brizzi M.F. (2019). Islet-like structures generated in vitro from adult human liver stem cells revert hyperglycemia in diabetic SCID mice. Stem Cell Rev. Rep..

[466] Navarro-Tableros V., Gomez Y., Brizzi M.F., Camussi G. (2020). Generation of human stem cell-derived pancreatic organoids (POs) for regenerative medicine. Adv. Exp. Med. Biol..

[467] El-Jawhari J.J., El-Sherbiny Y., McGonagle D., Jones E. (2021). Multipotent mesenchymal stromal cells in rheumatoid arthritis and systemic lupus erythematosus; from a leading role in pathogenesis to potential therapeutic saviors?. Front. Immunol..

[468] Huang Y., Wu Q., Tam P.K.H. (2022). Immunomodulatory mechanisms of mesenchymal stem cells and their potential clinical applications. Int. J. Mol. Sci..

[469] Markov A., Thangavelu L., Aravindhan S., Zekiy A.O., Jarahian M., Chartrand M.S., Pathak Y., Marofi F., Shamlou S., Hassanzadeh A. (2021). Mesenchymal stem/stromal cells as a valuable source for the treatment of immune-mediated disorders. Stem Cell Res. Ther..

[470] Freedman M.S., Bar-Or A., Atkins H.L., Karussis D., Frassoni F., Lazarus H., Scolding N., Slavin S., Le Blanc K., Uccelli A. (2010). The therapeutic potential of mesenchymal stem cell transplantation as a treatment for multiple sclerosis: Consensus report of the International MSCT Study Group. Mult. Scler. J..

[471] Paganelli A., Tarentini E., Benassi L., Kaleci S., Magnoni C. (2020). Mesenchymal stem cells for the treatment of psoriasis: A comprehensive review. Clin. Exp. Dermatol..

[472] Orozco L., Munar A., Soler R., Alberca M., Soler F., Huguet M., Sentís J., Sánchez A., García-Sancho J. (2013). Treatment of knee osteoarthritis with autologous mesenchymal stem cells: A pilot study. Transplantation.

[473] Chahal J., Gómez-Aristizábal A., Shestopaloff K., Bhatt S., Chaboureau A., Fazio A., Chisholm J., Weston A., Chiovitti J., Keating A. (2019). Bone marrow mesenchymal stromal cell treatment in patients with osteoarthritis results in overall improvement in pain and symptoms and reduces synovial inflammation. Stem Cells Transl. Med..

[474] Gerdoni E., Gallo B., Casazza S., Musio S., Bonanni I., Pedemonte E., Mantegazza R., Frassoni F., Mancardi G., Pedotti R. (2007). Mesenchymal stem cells effectively modulate pathogenic immune response in experimental autoimmune encephalomyelitis. Ann. Neurol..

[475] Shi M.Y., Liu L., Yang F.Y. (2022). Strategies to improve the effect of mesenchymal stem cell therapy on inflammatory bowel disease. World J. Stem Cells.

[476] Merimi M., El-Majzoub R., Lagneaux L., Moussa Agha D., Bouhtit F., Meuleman N., Fahmi H., Lewalle P., Fayyad-Kazan M., Najar M. (2021). The therapeutic potential of mesenchymal stromal cells for regenerative medicine: Current knowledge and future understandings. Front. Cell Dev. Biol..

[477] Heo J.S., Kim S., Yang C.E., Choi Y., Song S.Y., Kim H.O. (2021). Human adipose mesenchymal stem cell-derived exosomes: A key player in wound healing. Tissue Eng. Regen. Med..

[478] Han Y., Li X., Zhang Y., Han Y., Chang F., Ding J. (2019). Mesenchymal stem cells for regenerative medicine. Cells.

[479] Pileggi A. (2012). Mesenchymal stem cells for the treatment of diabetes. Diabetes.

[480] Madani S., Amanzadi M., Aghayan H.R., Setudeh A., Rezaei N., Rouhifard M., Larijani B. (2022). Investigating the safety and efficacy of hematopoietic and mesenchymal stem cell transplantation for treatment of T1DM: A systematic review and meta-analysis. Syst. Rev..

[481] Zhang J., Zheng Y., Huang L., He J. (2023). Research progress on mesenchymal stem cells for the treatment of diabetes and its complications. Int. J. Endocrinol..

[482] Izadi M., Sadr Hashemi Nejad A., Moazenchi M., Masoumi S., Rabbani A., Kompani F., Hedayati Asl A.A., Abbasi Kakroodi F., Jaroughi N., Mohseni Meybodi M.A. (2022). Mesenchymal stem cell transplantation in newly diagnosed type-1 diabetes patients: A phase I/II randomized placebo-controlled clinical trial. Stem Cell Res. Ther..

[483] Zhang Y., Chen W., Feng B., Cao H. (2020). The clinical efficacy and safety of stem cell therapy for diabetes mellitus: A systematic review and meta-analysis. Aging Dis..

[484] Vanikar A.V., Dave S.D., Thakkar U.G., Trivedi H.L. (2010). Cotransplantation of adipose tissue-derived insulin-secreting mesenchymal stem cells and hematopoietic stem cells: A novel therapy for insulin-dependent diabetes mellitus. Stem Cells Int..

[485] Trivedi H.L., Vanikar A.V., Thakker U., Firoze A., Dave S.D., Patel C.N., Patel J.V., Bhargava A.B., Shankar V. (2008). Human adipose tissue-derived mesenchymal stem cells combined with hematopoietic stem cell transplantation synthesize insulin. Transplant. Proc..

[486] Lian X.F., Lu D.H., Liu H.L., Liu Y.J., Han X.Q., Yang Y., Lin Y., Zeng Q.X., Huang Z.J., Xie F. (2022). Effectiveness and safety of human umbilical cord-mesenchymal stem cells for treating type 2 diabetes mellitus. World J. Diabetes.

[487] Cai J., Wu Z., Xu X., Liao L., Chen J., Huang L., Wu W., Luo F., Wu C., Pugliese A. (2016). Umbilical cord mesenchymal stromal cell with autologous bone marrow cell transplantation in established type 1 diabetes: A pilot randomized controlled open-label clinical study to assess safety and impact on insulin secretion. Diabetes Care.

[488] Carlsson P.O., Schwarcz E., Korsgren O., Le Blanc K. (2015). Preserved β-cell function in type 1 diabetes by mesenchymal stromal cells. Diabetes.

[489] Hu J., Yu X., Wang Z., Wang F., Wang L., Gao H., Chen Y., Zhao W., Jia Z., Yan S. (2013). Long term effects of the implantation of Wharton’s jelly-derived mesenchymal stem cells from the umbilical cord for newly-onset type 1 diabetes mellitus. Endocr. J..

[490] Carlsson P.O., Espes D., Sisay S., Davies L.C., Smith C.I.E., Svahn M.G. (2023). Umbilical cord-derived mesenchymal stromal cells preserve endogenous insulin production in type 1 diabetes: A Phase I/II randomised double-blind placebo-controlled trial. Diabetologia.

[491] Yang G., Fan X., Liu Y., Jie P., Mazhar M., Liu Y., Dechsupa N., Wang L. (2023). Immunomodulatory mechanisms and therapeutic potential of mesenchymal stem cells. Stem Cell Rev. Rep..

[492] Lin P., Correa D., Kean T.J., Awadallah A., Dennis J.E., Caplan A.I. (2014). Serial transplantation and long-term engraftment of intra-arterially delivered clonally derived mesenchymal stem cells to injured bone marrow. Mol. Ther..

[493] Chapel A., Bertho J.M., Bensidhoum M., Fouillard L., Young R.G., Frick J., Demarquay C., Cuvelier F., Mathieu E., Trompier F. (2003). Mesenchymal stem cells home to injured tissues when co-infused with hematopoietic cells to treat a radiation-induced multi-organ failure syndrome. J. Gene Med..

[494] Boumaza I., Srinivasan S., Witt W.T., Feghali-Bostwick C., Dai Y., Garcia-Ocana A., Feili-Hariri M. (2009). Autologous bone marrow-derived rat mesenchymal stem cells promote PDX-1 and insulin expression in the islets, alter T cell cytokine pattern and preserve regulatory T cells in the periphery and induce sustained normoglycemia. J. Autoimmun..

[495] Liu P., Cao B., Zhou Y., Zhang H., Wang C. (2023). Human umbilical cord-derived mesenchymal stem cells alleviate oxidative stress-induced islet impairment via the Nrf2/HO-1 axis. J. Mol. Cell. Biol..

[496] Ilieva A., Yuan S., Wang R.N., Agapitos D., Hill D.J., Rosenberg L. (1999). Pancreatic islet cell survival following islet isolation: The role of cellular interactions in the pancreas. J. Endocrinol..

[497] Giuliani M., Moritz W., Bodmer E., Dindo D., Kugelmeier P., Lehmann R., Gassmann M., Groscurth P., Weber M. (2005). Central necrosis in isolated hypoxic human pancreatic islets: Evidence for postisolation ischemia. Cell Transplant..

[498] Kin T., Senior P., O’Gorman D., Richer B., Salam A., Shapiro A.M. (2008). Risk factors for islet loss during culture prior to transplantation. Transpl. Int..

[499] Teo A.K.K., Lim C.S., Cheow L.F., Kin T., Shapiro J.A., Kang N.Y., Burkholder W., Lau H.H. (2018). Single-cell analyses of human islet cells reveal de-differentiation signatures. Cell Death Discov.

[500] Hubber E.L., Rackham C.L., Jones P.M. (2021). Protecting islet functional viability using mesenchymal stromal cells. Stem Cells Transl. Med..

[501] de Souza B.M., Bouças A.P., Oliveira F.D., Reis K.P., Ziegelmann P., Bauer A.C., Crispim D. (2017). Effect of co-culture of mesenchymal stem/stromal cells with pancreatic islets on viability and function outcomes: A systematic review and meta-analysis. Islets.

[502] Ding Y., Xu D., Feng G., Bushell A., Muschel R.J., Wood K.J. (2009). Mesenchymal stem cells prevent the rejection of fully allogenic islet grafts by the immunosuppressive activity of matrix metalloproteinase-2 and -9. Diabetes.

[503] Christoffersson G., Waldén T., Sandberg M., Opdenakker G., Carlsson P.O., Phillipson M. (2015). Matrix metalloproteinase-9 is essential for physiological β cell function and islet vascularization in adult mice. Am. J. Pathol..

[504] Park K.S., Kim Y.S., Kim J.H., Choi B.K., Kim S.H., Oh S.H., Ahn Y.R., Lee M.S., Lee M.K., Park J.B. (2009). Influence of human allogenic bone marrow and cord blood-derived mesenchymal stem cell secreting trophic factors on ATP (adenosine-5′-triphosphate)/ADP (adenosine-5′-diphosphate) ratio and insulin secretory function of isolated human islets from cadaveric donor. Transplant. Proc..

[505] Jung E.J., Kim S.C., Wee Y.M., Kim Y.H., Choi M.Y., Jeong S.H., Lee J., Lim D.G., Han D.J. (2011). Bone marrow-derived mesenchymal stromal cells support rat pancreatic islet survival and insulin secretory function in vitro. Cytotherapy.

[506] Wu H., Lu W., Mahato R.I. (2011). Mesenchymal stem cells as a gene delivery vehicle for successful islet transplantation. Pharm. Res..

[507] Wu H., Wen D., Mahato R.I. (2013). Third-party mesenchymal stem cells improved human islet transplantation in a humanized diabetic mouse model. Mol. Ther..

[508] Karaoz E., Okcu A., Ünal Z.S., Subasi C., Saglam O., Duruksu G. (2013). Adipose tissue-derived mesenchymal stromal cells efficiently differentiate into insulin-producing cells in pancreatic islet microenvironment both in vitro and in vivo. Cytotherapy.

[509] Karaoz E., Genç Z.S., Demircan P., Aksoy A., Duruksu G. (2010). Protection of rat pancreatic islet function and viability by coculture with rat bone marrow-derived mesenchymal stem cells. Cell Death Dis..

[510] Gao X., Song L., Shen K., Wang H., Qian M., Niu W., Qin X. (2014). Bone marrow mesenchymal stem cells promote the repair of islets from diabetic mice through paracrine actions. Mol. Cell Endocrinol..

[511] Rahavi H., Hashemi S.M., Soleimani M., Mohammadi J., Tajik N. (2015). Adipose tissue-derived mesenchymal stem cells exert in vitro immunomodulatory and beta cell protective functions in streptozotocin-induced diabetic mice model. J. Diabetes Res..

[512] Montanari E., Meier R.P.H., Mahou R., Seebach J.D., Wandrey C., Gerber-Lemaire S., Buhler L.H., Gonelle-Gispert C. (2017). Multipotent mesenchymal stromal cells enhance insulin secretion from human islets via N-cadherin interaction and prolong function of transplanted encapsulated islets in mice. Stem Cell Res. Ther..

[513] Okcu A., Yazir Y., Şimşek T., Mert S., Duruksu G., Öztürk A., Kiliç K.C., Akpinar G., Kasap M. (2023). Investigation of the effect of pancreatic decellularized matrix on encapsulated Islets of Langerhans with mesenchymal stem cells. Tissue Cell.

[514] Banerjee M., Kumar A., Bhonde R.R. (2005). Reversal of experimental diabetes by multiple bone marrow transplantation. Biochem. Biophys. Res. Commun..

[515] Ezquer F.E., Ezquer M.E., Parrau D.B., Carpio D., Yañez A.J., Conget P.A. (2008). Systemic administration of multipotent mesenchymal stromal cells reverts hyperglycemia and prevents nephropathy in type 1 diabetic mice. Biol. Blood Marrow Transplant..

[516] Fiorina P., Jurewicz M., Augello A., Vergani A., Dada S., La Rosa S., Selig M., Godwin J., Law K., Placidi C. (2009). Immunomodulatory function of bone marrow-derived mesenchymal stem cells in experimental autoimmune type 1 diabetes. J. Immunol..

[517] Ianus A., Holz G.G., Theise N.D., Hussain M.A. (2003). In vivo derivation of glucose-competent pancreatic endocrine cells from bone marrow without evidence of cell fusion. J. Clin. Investig..

[518] Gao X., Song L., Shen K., Wang H., Niu W., Qin X. (2008). Transplantation of bone marrow derived cells promotes pancreatic islet repair in diabetic mice. Biochem. Biophys. Res. Commun..

[519] Hasegawa Y., Ogihara T., Yamada T., Ishigaki Y., Imai J., Uno K., Gao J., Kaneko K., Ishihara H., Sasano H. (2007). Bone marrow (BM) transplantation promotes β-cell regeneration after acute injury through BM cell mobilization. Endocrinology.

[520] Hess D., Li L., Martin M., Sakano S., Hill D., Strutt B., Thyssen S., Gray D.A., Bhatia M. (2003). Bone marrow-derived stem cells initiate pancreatic regeneration. Nat. Biotechnol..

[521] Lee R.H., Seo M.J., Reger R.L., Spees J.L., Pulin A.A., Olson S.D., Prockop D.J. (2006). Multipotent stromal cells from human marrow home to and promote repair of pancreatic islets and renal glomeruli in diabetic NOD/scid mice. Proc. Natl. Acad. Sci. USA.

[522] Zhao M., Amiel S.A., Ajami S., Jiang J., Rela M., Heaton N., Huang G.C. (2008). Amelioration of streptozotocin-induced diabetes in mice with cells derived from human marrow stromal cells. PLoS ONE.

[523] Dong Q.Y., Chen L., Gao G.Q., Wang L., Song J., Chen B., Xu Y.X., Sun L. (2008). Allogeneic diabetic mesenchymal stem cells transplantation in streptozotocin-induced diabetic rat. Clin. Investig. Med..

[524] Figliuzzi M., Cornolti R., Perico N., Rota C., Morigi M., Remuzzi G., Remuzzi A., Benigni A. (2009). Bone marrow-derived mesenchymal stem cells improve islet graft function in diabetic rats. Transplant. Proc..

[525] Solari M.G., Srinivasan S., Boumaza I., Unadkat J., Harb G., Garcia-Ocana A., Feili-Hariri M. (2009). Marginal mass islet transplantation with autologous mesenchymal stem cells promotes long-term islet allograft survival and sustained normoglycemia. J. Autoimmun..

[526] Jurewicz M., Yang S., Augello A., Godwin J.G., Moore R.F., Azzi J., Fiorina P., Atkinson M., Sayegh M.H., Abdi R. (2010). Congenic mesenchymal stem cell therapy reverses hyperglycemia in experimental type 1 diabetes. Diabetes.

[527] Ito T., Itakura S., Todorov I., Rawson J., Asari S., Shintaku J., Nair I., Ferreri K., Kandeel F., Mullen Y. (2010). Mesenchymal stem cell and islet co-transplantation promotes graft revascularization and function. Transplantation.

[528] Sakata N., Chan N.K., Chrisler J., Obenaus A., Hathout E. (2010). Bone marrow cell cotransplantation with islets improves their vascularization and function. Transplantation.

[529] Ohmura Y., Tanemura M., Kawaguchi N., Machida T., Tanida T., Deguchi T., Wada H., Kobayashi S., Marubashi S., Eguchi H. (2010). Combined transplantation of pancreatic islets and adipose tissue-derived stem cells enhances the survival and insulin function of islet grafts in diabetic mice. Transplantation.

[530] Rackham C.L., Chagastelles P.C., Nardi N.B., Hauge-Evans A.C., Jones P.M., King A.J. (2011). Co-transplantation of mesenchymal stem cells maintains islet organisation and morphology in mice. Diabetologia.

[531] Cavallari G., Olivi E., Bianchi F., Neri F., Foroni L., Valente S., La Manna G., Nardo B., Stefoni S., Ventura C. (2012). Mesenchymal stem cells and islet cotransplantation in diabetic rats: Improved islet graft revascularization and function by human adipose tissue-derived stem cells preconditioned with natural molecules. Cell Transplant..

[532] Milanesi A., Lee J.W., Li Z., Da Sacco S., Villani V., Cervantes V., Perin L., Yu J.S. (2012). β-Cell regeneration mediated by human bone marrow mesenchymal stem cells. PLoS ONE.

[533] Katuchova J., Tothova T., Farkasova Iannaccone S., Toporcer T., Harvanova D., Hildebrand T., Kilik R., Bacenkova D., Frohlichova L., Rosocha J. (2012). Impact of different pancreatic microenvironments on improvement in hyperglycemia and insulin deficiency in diabetic rats after transplantation of allogeneic mesenchymal stromal cells. J. Surg. Res..

[534] Bell G.I., Broughton H.C., Levac K.D., Allan D.A., Xenocostas A., Hess D.A. (2012). Transplanted human bone marrow progenitor subtypes stimulate endogenous islet regeneration and revascularization. Stem Cells Dev..

[535] Si Y., Zhao Y., Hao H., Liu J., Guo Y., Mu Y., Shen J., Cheng Y., Fu X., Han W. (2012). Infusion of mesenchymal stem cells ameliorates hyperglycemia in type 2 diabetic rats: Identification of a novel role in improving insulin sensitivity. Diabetes.

[536] Tsai P.J., Wang H.S., Shyr Y.M., Weng Z.C., Tai L.C., Shyu J.F., Chen T.H. (2012). Transplantation of insulin-producing cells from umbilical cord mesenchymal stem cells for the treatment of streptozotocin-induced diabetic rats. J. Biomed. Sci..

[537] Hao H., Liu J., Shen J., Zhao Y., Liu H., Hou Q., Tong C., Ti D., Dong L., Cheng Y. (2013). Multiple intravenous infusions of bone marrow mesenchymal stem cells reverse hyperglycemia in experimental type 2 diabetes rats. Biochem. Biophys. Res. Commun..

[538] Bhang S.H., Jung M.J., Shin J.Y., La W.G., Hwang Y.H., Kim M.J., Kim B.S., Lee D.Y. (2013). Mutual effect of subcutaneously transplanted human adipose-derived stem cells and pancreatic islets within fibrin gel. Biomaterials.

[539] Borg D.J., Weigelt M., Wilhelm C., Gerlach M., Bickle M., Speier S., Bonifacio E., Hommel A. (2014). Mesenchymal stromal cells improve transplanted islet survival and islet function in a syngeneic mouse model. Diabetologia.

[540] Hirabaru M., Kuroki T., Adachi T., Kitasato A., Ono S., Tanaka T., Matsushima H., Sakai Y., Soyama A., Hidaka M. (2015). A method for performing islet transplantation using tissue-engineered sheets of islets and mesenchymal stem cells. Tissue Eng. Part C Methods.

[541] Hu J., Wang Y., Wang F., Wang L., Yu X., Sun R., Wang Z., Wang L., Gao H., Fu Z. (2015). Effect and mechanisms of human Wharton’s jelly-derived mesenchymal stem cells on type 1 diabetes in NOD model. Endocrine.

[542] Yaochite J.N., Caliari-Oliveira C., de Souza L.E., Neto L.S., Palma P.V., Covas D.T., Malmegrim K.C., Voltarelli J.C., Donadi E.A. (2015). Therapeutic efficacy and biodistribution of allogeneic mesenchymal stem cells delivered by intrasplenic and intrapancreatic routes in streptozotocin-induced diabetic mice. Stem Cell Res. Ther..

[543] Tsai P.J., Wang H.S., Lin G.J., Chou S.C., Chu T.H., Chuan W.T., Lu Y.J., Weng Z.C., Su C.H., Hsieh P.S. (2015). Undifferentiated Wharton’s Jelly mesenchymal stem cell transplantation induces insulin-producing cell differentiation and suppression of t-cell-mediated autoimmunity in nonobese diabetic mice. Cell Transplant..

[544] Ben Nasr M., Vergani A., Avruch J., Liu L., Kefaloyianni E., D’Addio F., Tezza S., Corradi D., Bassi R., Valderrama-Vasquez A. (2015). Co-transplantation of autologous MSCs delays islet allograft rejection and generates a local immunoprivileged site. Acta Diabetol..

[545] Li L.R., Jia X.L., Hui H., Zhang J., Liu Y., Cui W.J., Xu Q.Y., Zhu D.L. (2016). Liraglutide enhances the efficacy of human mesenchymal stem cells in preserving islet β-cell function in severe non-obese diabetic mice. Mol. Med..

[546] Mohammadi Ayenehdeh J., Niknam B., Rasouli S., Hashemi S.M., Rahavi H., Rezaei N., Soleimani M., Liaeiha A., Niknam M.H., Tajik N. (2017). Immunomodulatory and protective effects of adipose tissue-derived mesenchymal stem cells in an allograft islet composite transplantation for experimental autoimmune type 1 diabetes. Immunol. Lett..

[547] Sun L.L., Liu T.J., Li L., Tang W., Zou J.J., Chen X.F., Zheng J.Y., Jiang B.G., Shi Y.Q. (2017). Transplantation of betatrophin-expressing adipose-derived mesenchymal stem cells induces β-cell proliferation in diabetic mice. Int. J. Mol. Med..

[548] Xiang C., Xie Q.P. (2018). Protection of mouse pancreatic islet function by co-culture with hypoxia pre-treated mesenchymal stromal cells. Mol. Med. Rep..

[549] Wang M., Song L., Strange C., Dong X., Wang H. (2018). Therapeutic effects of adipose stem cells from diabetic mice for the treatment of type 2 diabetes. Mol. Ther..

[550] Navaei-Nigjeh M., Moloudizargari M., Baeeri M., Gholami M., Lotfibakhshaiesh N., Soleimani M., Vasheghani-Farahani E., Ai J., Abdollahi M. (2018). Reduction of marginal mass required for successful islet transplantation in a diabetic rat model using adipose tissue-derived mesenchymal stromal cells. Cytotherapy.

[551] Ren G., Rezaee M., Razavi M., Taysir A., Wang J., Thakor A.S. (2019). Adipose tissue-derived mesenchymal stem cells rescue the function of islets transplanted in sub-therapeutic numbers via their angiogenic properties. Cell Tissue Res..

[552] Khatri R., Mazurek S., Petry S.F., Linn T. (2020). Mesenchymal stem cells promote pancreatic β-cell regeneration through downregulation of FoxO1 pathway. Stem Cell Res. Ther..

[553] Isildar B., Ozkan S., Ercin M., Gezginci-Oktayoglu S., Oncul M., Koyuturk M. (2022). 2D and 3D cultured human umbilical cord-derived mesenchymal stem cell-conditioned medium has a dual effect in type 1 diabetes model in rats: Immunomodulation and β-cell regeneration. Inflamm. Regen..

[554] Ahmed O.M., Saleh A.S., Ahmed E.A., Ghoneim M.M., Ebrahim H.A., Abdelgawad M.A., Abdel-Gabbar M. (2023). Efficiency of bone marrow-derived mesenchymal stem cells and hesperetin in the treatment of streptozotocin-induced type 1 diabetes in Wistar rats. Pharmaceuticals.

[555] Bell G.I., Meschino M.T., Hughes-Large J.M., Broughton H.C., Xenocostas A., Hess D.A. (2012). Combinatorial human progenitor cell transplantation optimizes islet regeneration through secretion of paracrine factors. Stem Cells Dev..

[556] Ye L., Li L., Wan B., Yang M., Hong J., Gu W., Wang W., Ning G. (2017). Immune response after autologous hematopoietic stem cell transplantation in type 1 diabetes mellitus. Stem Cell Res. Ther..

[557] Mesples A., Majeed N., Zhang Y., Hu X. (2013). Early immunotherapy using autologous adult stem cells reversed the effect of anti-pancreatic islets in recently diagnosed type 1 diabetes mellitus: Preliminary results. Med. Sci. Monit..

[558] Voltarelli J.C., Couri C.E., Stracieri A.B., Oliveira M.C., Moraes D.A., Pieroni F., Coutinho M., Malmegrim K.C., Foss-Freitas M.C., Simões B.P. (2007). Autologous nonmyeloablative hematopoietic stem cell transplantation in newly diagnosed type 1 diabetes mellitus. JAMA.

[559] Couri C.E., Oliveira M.C., Stracieri A.B., Moraes D.A., Pieroni F., Barros G.M., Madeira M.I., Malmegrim K.C., Foss-Freitas M.C., Simões B.P. (2009). C-peptide levels and insulin independence following autologous nonmyeloablative hematopoietic stem cell transplantation in newly diagnosed type 1 diabetes mellitus. JAMA.

[560] Snarski E., Torosian T., Paluszewska M., Urbanowska E., Milczarczyk A., Jedynasty K., Franek E., Jedrzejczak W.W. (2009). Alleviation of exogenous insulin requirement in type 1 diabetes mellitus after immunoablation and transplantation of autologous hematopoietic stem cells. Pol. Arch. Med. Wewn..

[561] Snarski E., Milczarczyk A., Torosian T., Paluszewska M., Urbanowska E., Król M., Boguradzki P., Jedynasty K., Franek E., Wiktor-Jedrzejczak W. (2011). Independence of exogenous insulin following immunoablation and stem cell reconstitution in newly diagnosed diabetes type I. Bone Marrow Transplant..

[562] Jiang R., Han Z., Zhuo G., Qu X., Li X., Wang X., Shao Y., Yang S., Han Z.C. (2011). Transplantation of placenta-derived mesenchymal stem cells in type 2 diabetes: A pilot study. Front. Med..

[563] Li L., Shen S., Ouyang J., Hu Y., Hu L., Cui W., Zhang N., Zhuge Y.Z., Chen B., Xu J. (2012). Autologous hematopoietic stem cell transplantation modulates immunocompetent cells and improves β-cell function in Chinese patients with new onset of type 1 diabetes. J. Clin. Endocrinol. Metab..

[564] Gu W., Hu J., Wang W., Li L., Tang W., Sun S., Cui W., Ye L., Zhang Y., Hong J. (2012). Diabetic ketoacidosis at diagnosis influences complete remission after treatment with hematopoietic stem cell transplantation in adolescents with type 1 diabetes. Diabetes Care.

[565] Shen S., Li L., Ouyang J., Xu J., Zhu D. (2012). Remission induced by autologous hematopoietic stem cell transplantation in one newly diagnosed type 1 diabetes patient with diabetic ketoacidosis: A case report. J. Diabetes.

[566] Kong D., Zhuang X., Wang D., Qu H., Jiang Y., Li X., Wu W., Xiao J., Liu X., Liu J. (2014). Umbilical cord mesenchymal stem cell transfusion ameliorated hyperglycemia in patients with type 2 diabetes mellitus. Clin. Lab..

[567] Bhansali A., Asokumar P., Walia R., Bhansali S., Gupta V., Jain A., Sachdeva N., Sharma R.R., Marwaha N., Khandelwal N. (2014). Efficacy and safety of autologous bone marrow-derived stem cell transplantation in patients with type 2 diabetes mellitus: A randomized placebo-controlled study. Cell Transplant..

[568] D’Addio F., Valderrama Vasquez A., Ben Nasr M., Franek E., Zhu D., Li L., Ning G., Snarski E., Fiorina P. (2014). Autologous nonmyeloablative hematopoietic stem cell transplantation in new-onset type 1 diabetes: A multicenter analysis. Diabetes.

[569] Dave S.D., Trivedi H.L., Gopal S.C., Chandra T. (2014). Combined therapy of insulin-producing cells and haematopoietic stem cells offers better diabetic control than only haematopoietic stem cells’ infusion for patients with insulin-dependent diabetes. BMJ Case Rep..

[570] Thakkar U.G., Trivedi H.L., Vanikar A.V., Dave S.D. (2015). Insulin-secreting adipose-derived mesenchymal stromal cells with bone marrow-derived hematopoietic stem cells from autologous and allogenic sources for type 1 diabetes mellitus. Cytotherapy.

[571] Snarski E., Milczarczyk A., Hałaburda K., Torosian T., Paluszewska M., Urbanowska E., Król M., Boguradzki P., Jedynasty K., Franek E. (2016). Immunoablation and autologous hematopoietic stem cell transplantation in the treatment of new-onset type 1 diabetes mellitus: Long-term observations. Bone Marrow Transplant..

[572] Cantú-Rodríguez O.G., Lavalle-González F., Herrera-Rojas M., Jaime-Pérez J.C., Hawing-Zárate J., Gutiérrez-Aguirre C.H., Mancias-Guerra C., González-Llano O., Zapata-Garrido A., Villarreal-Pérez J.Z. (2016). Long-term insulin independence in type 1 diabetes mellitus using a simplified autologous stem cell transplant. J. Clin. Endocrinol. Metab..

[573] Li L., Hui H., Jia X., Zhang J., Liu Y., Xu Q., Zhu D. (2016). Infusion with human bone marrow-derived mesenchymal stem cells improves β-cell function in patients and non-obese mice with severe diabetes. Sci. Rep..

[574] Bhansali S., Dutta P., Kumar V., Yadav M.K., Jain A., Mudaliar S., Bhansali S., Sharma R.R., Jha V., Marwaha N. (2017). Efficacy of autologous bone marrow-derived mesenchymal stem cell and mononuclear cell transplantation in Type 2 Diabetes Mellitus: A randomized, placebo-controlled comparative study. Stem Cells Dev..

[575] Gu B., Miao H., Zhang J., Hu J., Zhou W., Gu W., Wang W., Ning G. (2018). Clinical benefits of autologous haematopoietic stem cell transplantation in type 1 diabetes patients. Diabetes Metab..

[576] Ulyanova O., Askarov M., Kozina L., Karibekov T., Shaimardanova G., Zhakupova A., Danilova D., Serebrennikova D. (2019). Autologous mesenchymal stem cell transplant in patients with type 1 diabetes mellitus. Exp. Clin. Transplant..

[577] Lu J., Shen S.M., Ling Q., Wang B., Li L.R., Zhang W., Qu D.D., Bi Y., Zhu D.L. (2021). One repeated transplantation of allogeneic umbilical cord mesenchymal stromal cells in type 1 diabetes: An open parallel controlled clinical study. Stem Cell Res. Ther..

[578] Zang L., Li Y., Hao H., Liu J., Cheng Y., Li B., Yin Y., Zhang Q., Gao F., Wang H. (2022). Efficacy and safety of umbilical cord-derived mesenchymal stem cells in Chinese adults with type 2 diabetes: A single-center, double-blinded, randomized, placebo-controlled phase II trial. Stem Cell Res. Ther..

[579] Mesples A.D., Cox D.C.T., Lundy H.D., Antonio-Collie S., Diggiss C.W., Lakey J.R.T. (2023). Monitoring of autoantibodies following autologous hematopoietic stem cell transplantation in 6 children with recently diagnosed type 1 diabetes mellitus. Med. Sci. Monit..

[580] Kupcova Skalnikova H. (2013). Proteomic techniques for characterisation of mesenchymal stem cell secretome. Biochimie.

[581] Brandhorst H., Brandhorst D., Abraham A., Acreman S., Schive S.W., Scholz H., Johnson P.R.V. (2020). Proteomic profiling reveals the ambivalent character of the mesenchymal stem cell secretome: Assessing the effect of preconditioned media on isolated human Islets. Cell Transplant..

[582] Lee M.J., Kim J., Kim M.Y., Bae Y.S., Ryu S.H., Lee T.G., Kim J.H. (2010). Proteomic analysis of tumor necrosis factor-α-induced secretome of human adipose tissue-derived mesenchymal stem cells. J. Proteome Res..

[583] Zhou Y., Yamamoto Y., Xiao Z., Ochiya T. (2019). The immunomodulatory functions of mesenchymal stromal/stem cells mediated via paracrine activity. J. Clin. Med..

[584] Meng X., Sun B., Xiao Z. (2019). Comparison in transcriptome and cytokine profiles of mesenchymal stem cells from human umbilical cord and cord blood. Gene.

[585] Boomsma R.A., Geenen D.L. (2012). Mesenchymal stem cells secrete multiple cytokines that promote angiogenesis and have contrasting effects on chemotaxis and apoptosis. PLoS ONE.

[586] Shen C., Lie P., Miao T., Yu M., Lu Q., Feng T., Li J., Zu T., Liu X., Li H. (2015). Conditioned medium from umbilical cord mesenchymal stem cells induces migration and angiogenesis. Mol. Med. Rep..

[587] Bai L., Li D., Li J., Luo Z., Yu S., Cao S., Shen L., Zuo Z., Ma X. (2016). Bioactive molecules derived from umbilical cord mesenchymal stem cells. Acta Histochem..

[588] Päth G., Perakakis N., Mantzoros C.S., Seufert J. (2019). Stem cells in the treatment of diabetes mellitus—Focus on mesenchymal stem cells. Metabolism.

[589] Drobiova H., Sindhu S., Ahmad R., Haddad D., Al-Mulla F., Al Madhoun A. (2023). Wharton’s jelly mesenchymal stem cells: A concise review of their secretome and prospective clinical applications. Front. Cell Dev. Biol..

[590] Dietrich I., Girdlestone J., Giele H. (2022). Differential cytokine expression in direct and indirect co-culture of islets and mesenchymal stromal cells. Cytokine.

[591] Ahangar P., Mills S.J., Cowin A.J. (2020). Mesenchymal stem cell secretome as an emerging cell-free alternative for improving wound repair. Int. J. Mol. Sci..

[592] Kumar P., Kandoi S., Misra R., Vijayalakshmi S., Rajagopal K., Verma R.S. (2019). The mesenchymal stem cell secretome: A new paradigm towards cell-free therapeutic mode in regenerative medicine. Cytokine Growth Factor Rev..

[593] Shigemoto-Kuroda T., Oh J.Y., Kim D.K., Jeong H.J., Park S.Y., Lee H.J., Park J.W., Kim T.W., An S.Y., Prockop D.J. (2017). MSC-derived extracellular vesicles attenuate immune responses in two autoimmune murine models: Type 1 Diabetes and uveoretinitis. Stem Cell Rep..

[594] Park C.W., Kim K.S., Bae S., Son H.K., Myung P.K., Hong H.J., Kim H. (2009). Cytokine secretion profiling of human mesenchymal stem cells by antibody array. Int. J. Stem Cells.

[595] Crigler L., Robey R.C., Asawachaicharn A., Gaupp D., Phinney D.G. (2006). Human mesenchymal stem cell subpopulations express a variety of neuro-regulatory molecules and promote neuronal cell survival and neuritogenesis. Exp. Neurol..

[596] Nemeth K., Keane-Myers A., Brown J.M., Metcalfe D.D., Gorham J.D., Bundoc V.G., Hodges M.G., Jelinek I., Madala S., Karpati S. (2010). Bone marrow stromal cells use TGF-beta to suppress allergic responses in a mouse model of ragweed-induced asthma. Proc. Natl. Acad. Sci. USA.

[597] Liang C., Jiang E., Yao J., Wang M., Chen S., Zhou Z., Zhai W., Ma Q., Feng S., Han M. (2018). Interferon-γ mediates the immunosuppression of bone marrow mesenchymal stem cells on T-lymphocytes in vitro. Hematology.

[598] Ejtehadifar M., Shamsasenjan K., Movassaghpour A., Akbarzadehlaleh P., Dehdilani N., Abbasi P., Molaeipour Z., Saleh M. (2015). The effect of hypoxia on mesenchymal stem cell biology. Adv. Pharm. Bull..

[599] Lee E.Y., Xia Y., Kim W.S., Kim M.H., Kim T.H., Kim K.J., Park B.S., Sung J.H. (2009). Hypoxia-enhanced wound-healing function of adipose-derived stem cells: Increase in stem cell proliferation and up-regulation of VEGF and bFGF. Wound Repair Regen..

[600] Paquet J., Deschepper M., Moya A., Logeart-Avramoglou D., Boisson-Vidal C., Petite H. (2015). Oxygen tension regulates human mesenchymal stem cell paracrine functions. Stem Cells Transl. Med..

[601] Nakanishi C., Nagaya N., Ohnishi S., Yamahara K., Takabatake S., Konno T., Hayashi K., Kawashiri M.A., Tsubokawa T., Yamagishi M. (2011). Gene and protein expression analysis of mesenchymal stem cells derived from rat adipose tissue and bone marrow. Circ. J..

[602] Wu Y., Chen L., Scott P.G., Tredget E.E. (2007). Mesenchymal stem cells enhance wound healing through differentiation and angiogenesis. Stem Cells.

[603] Rossignol J., Boyer C., Lévèque X., Fink K.D., Thinard R., Blanchard F., Dunbar G.L., Lescaudron L. (2011). Mesenchymal stem cell transplantation and DMEM administration in a 3NP rat model of Huntington’s disease: Morphological and behavioral outcomes. Behav. Brain Res..

[604] Luo Q., Zhang B., Kuang D., Song G. (2016). Role of stromal-derived factor-1 in mesenchymal stem cell paracrine-mediated tissue repair. Curr Stem Cell Res. Ther..

[605] Kinnaird T., Stabile E., Burnett M.S., Lee C.W., Barr S., Fuchs S., Epstein S.E. (2004). Marrow-derived stromal cells express genes encoding a broad spectrum of arteriogenic cytokines and promote in vitro and in vivo arteriogenesis through paracrine mechanisms. Circ. Res..

[606] Hematti P., Kim J., Stein A.P., Kaufman D. (2013). Potential role of mesenchymal stromal cells in pancreatic islet transplantation. Transplant. Rev..

[607] Buravkova L.B., Andreeva E.R., Gogvadze V., Zhivotovsky B. (2014). Mesenchymal stem cells and hypoxia: Where are we?. Mitochondrion.

[608] Yusoff F.M., Nakashima A., Kawano K.I., Kajikawa M., Kishimoto S., Maruhashi T., Ishiuchi N., Abdul Wahid S.F.S., Higashi Y. (2022). Implantation of hypoxia-induced mesenchymal stem cell advances therapeutic angiogenesis. Stem Cells Int..

[609] Gala D.N., Fabian Z. (2021). To breathe or not to breathe: The role of oxygen in bone marrow-derived mesenchymal stromal cell senescence. Stem Cells Int..

[610] Sazli B.I., Lindarto D., Hasan R., Putra A., Pranoto A., Sembiring R.J., Ilyas S., Syafril S. (2023). Secretome of hypoxia-preconditioned mesenchymal stem cells enhance angiogenesis in diabetic rats with peripheral artery disease. Med. Arch..

[611] Kwon Y.W., Heo S.C., Jeong G.O., Yoon J.W., Mo W.M., Lee M.J., Jang I.H., Kwon S.M., Lee J.S., Kim J.H. (2013). Tumor necrosis factor-α-activated mesenchymal stem cells promote endothelial progenitor cell homing and angiogenesis. Biochim. Biophys. Acta.

[612] Wang M., Crisostomo P.R., Herring C., Meldrum K.K., Meldrum D.R. (2006). Human progenitor cells from bone marrow or adipose tissue produce VEGF, HGF, and IGF-I in response to TNF by a p38 MAPK-dependent mechanism. Am. J. Physiol. Regul. Integr. Comp. Physiol..

[613] Kilroy G.E., Foster S.J., Wu X., Ruiz J., Sherwood S., Heifetz A., Ludlow J.W., Stricker D.M., Potiny S., Green P. (2007). Cytokine profile of human adipose-derived stem cells: Expression of angiogenic, hematopoietic, and pro-inflammatory factors. J. Cell Physiol..

[614] Lu Y., Jin X., Chen Y., Li S., Yuan Y., Mai G., Tian B., Long D., Zhang J., Zeng L. (2010). Mesenchymal stem cells protect islets from hypoxia/reoxygenation-induced injury. Cell Biochem. Funct..

[615] Laporte C., Tubbs E., Cristante J., Gauchez A.S., Pesenti S., Lamarche F., Cottet-Rousselle C., Garrel C., Moisan A., Moulis J.M. (2019). Human mesenchymal stem cells improve rat islet functionality under cytokine stress with combined upregulation of heme oxygenase-1 and ferritin. Stem Cell Res. Ther..

[616] da Silva Meirelles L., Fontes A.M., Covas D.T., Caplan A.I. (2009). Mechanisms involved in the therapeutic properties of mesenchymal stem cells. Cytokine Growth Factor Rev..

[617] Li M., Sun X., Kuang X., Liao Y., Li H., Luo D. (2014). Mesenchymal stem cells suppress CD8^+^ T cell-mediated activation by suppressing natural killer group 2, member D protein receptor expression and secretion of prostaglandin E2, indoleamine 2, 3-dioxygenase and transforming growth factor-β. Clin. Exp. Immunol..

[618] Meisel R., Zibert A., Laryea M., Göbel U., Däubener W., Dilloo D. (2004). Human bone marrow stromal cells inhibit allogeneic T-cell responses by indoleamine 2,3-dioxygenase-mediated tryptophan degradation. Blood.

[619] DelaRosa O., Lombardo E., Beraza A., Mancheño-Corvo P., Ramirez C., Menta R., Rico L., Camarillo E., García L., Abad J.L. (2009). Requirement of IFN-γ-mediated indoleamine 2,3-dioxygenase expression in the modulation of lymphocyte proliferation by human adipose-derived stem cells. Tissue Eng. Part A.

[620] Sato K., Ozaki K., Oh I., Meguro A., Hatanaka K., Nagai T., Muroi K., Ozawa K. (2007). Nitric oxide plays a critical role in suppression of T-cell proliferation by mesenchymal stem cells. Blood.

[621] Chabannes D., Hill M., Merieau E., Rossignol J., Brion R., Soulillou J.P., Anegon I., Cuturi M.C. (2007). A role for heme oxygenase-1 in the immunosuppressive effect of adult rat and human mesenchymal stem cells. Blood.

[622] Sala E., Genua M., Petti L., Anselmo A., Arena V., Cibella J., Zanotti L., D’Alessio S., Scaldaferri F., Luca G. (2015). Mesenchymal stem cells reduce colitis in mice via release of TSG6, independently of their localization to the intestine. Gastroenterology.

[623] Kim D.S., Jang I.K., Lee M.W., Ko Y.J., Lee D.H., Lee J.W., Sung K.W., Koo H.H., Yoo K.H. (2018). Enhanced immunosuppressive properties of human mesenchymal stem cells primed by interferon-γ. EBioMedicine.

[624] Yu Y., Yoo S.M., Park H.H., Baek S.Y., Kim Y.J., Lee S., Kim Y.L., Seo K.W., Kang K.S. (2019). Preconditioning with interleukin-1β and interferon-γenhances the efficacy of human umbilical cord blood-derived mesenchymal stem cells-based therapy via enhancing prostaglandin E2 secretion and indoleamine 2,3-dioxygenase activity in dextran sulfate sodium-induced colitis. J. Tissue Eng. Regen. Med..

[625] Sheng H., Wang Y., Jin Y., Zhang Q., Zhang Y., Wang L., Shen B., Yin S., Liu W., Cui L. (2008). A critical role of IFNγ in priming MSC-mediated suppression of T cell proliferation through up-regulation of B7-H1. Cell Res..

[626] Giri J., Das R., Nylen E., Chinnadurai R., Galipeau J. (2020). CCL2 and CXCL12 Derived from mesenchymal stromal cells cooperatively polarize IL-10^+^ tissue macrophages to mitigate gut injury. Cell Rep..

[627] Luz-Crawford P., Noël D., Fernandez X., Khoury M., Figueroa F., Carrión F., Jorgensen C., Djouad F. (2012). Mesenchymal stem cells repress Th17 molecular program through the PD-1 pathway. PLoS ONE.

[628] Davies L.C., Heldring N., Kadri N., Le Blanc K. (2017). Mesenchymal stromal cell secretion of Programmed Death-1 ligands regulates T cell mediated immunosuppression. Stem Cells.

[629] Selleri S., Dieng M.M., Nicoletti S., Louis I., Beausejour C., Le Deist F., Haddad E. (2013). Cord-blood-derived mesenchymal stromal cells downmodulate CD4^+^ T-cell activation by inducing IL-10-producing Th1 cells. Stem Cells Dev..

[630] Kou M., Huang L., Yang J., Chiang Z., Chen S., Liu J., Guo L., Zhang X., Zhou X., Xu X. (2022). Mesenchymal stem cell-derived extracellular vesicles for immunomodulation and regeneration: A next generation therapeutic tool?. Cell Death Dis..

[631] Karnas E., Dudek P., Zuba-Surma E.K. (2023). Stem cell- derived extracellular vesicles as new tools in regenerative medicine—Immunomodulatory role and future perspectives. Front. Immunol..

[632] Reis M., Mavin E., Nicholson L., Green K., Dickinson A.M., Wang X.N. (2018). Mesenchymal stromal cell-derived extracellular vesicles attenuate dendritic cell maturation and function. Front. Immunol..

[633] Favaro E., Carpanetto A., Lamorte S., Fusco A., Caorsi C., Deregibus M.C., Bruno S., Amoroso A., Giovarelli M., Porta M. (2014). Human mesenchymal stem cell-derived microvesicles modulate T cell response to islet antigen glutamic acid decarboxylase in patients with type 1 diabetes. Diabetologia.

[634] Nojehdehi S., Soudi S., Hesampour A., Rasouli S., Soleimani M., Hashemi S.M. (2018). Immunomodulatory effects of mesenchymal stem cell-derived exosomes on experimental type-1 autoimmune diabetes. J. Cell Biochem..

[635] Shi H., Hao X., Sun Y., Zhang H., Zhao Y., Wang B., Lu J., Hou W., Yan Y., Yu X. (2023). Bone marrow mesenchymal stem cell-derived exosomes reduce insulin resistance and obesity in mice via the PI3K/AKT signaling pathway. FEBS Open Bio.

[636] Fuloria S., Subramaniyan V., Dahiya R., Dahiya S., Sudhakar K., Kumari U., Sathasivam K., Meenakshi D.U., Wu Y.S., Sekar M. (2021). Mesenchymal stem cell-derived extracellular vesicles: Regenerative potential and challenges. Biology.

[637] Luo H., Birjandi A.A., Ren F., Sun T., Sharpe P.T., Sun H., An Z. (2024). Advances in oral mesenchymal stem cell-derived extracellular vesicles in health and disease. Genes Dis..

[638] Ding J.Y., Chen M.J., Wu L.F., Shu G.F., Fang S.J., Li Z.Y., Chu X.R., Li X.K., Wang Z.G., Ji J.S. (2023). Mesenchymal stem cell-derived extracellular vesicles in skin wound healing: Roles, opportunities and challenges. Mil. Med. Res..

[639] Kosanović M., Milutinović B., Kutzner T.J., Mouloud Y., Bozic M. (2023). Clinical prospect of mesenchymal stromal/stem cell-derived extracellular vesicles in kidney disease: Challenges and the way forward. Pharmaceutics.

[640] Wiest E.F., Zubair A.C. (2020). Challenges of manufacturing mesenchymal stromal cell-derived extracellular vesicles in regenerative medicine. Cytotherapy.

[641] An T., Chen Y., Tu Y., Lin P. (2021). Mesenchymal stromal cell-derived extracellular vesicles in the treatment of diabetic foot ulcers: Application and challenges. Stem Cell Rev. Rep..

[642] Jin J., Shi Y., Gong J., Zhao L., Li Y., He Q., Huang H. (2019). Exosome secreted from adipose-derived stem cells attenuates diabetic nephropathy by promoting autophagy flux and inhibiting apoptosis in podocyte. Stem Cell Res. Ther..

[643] Ebrahim N., Ahmed I.A., Hussien N.I., Dessouky A.A., Farid A.S., Elshazly A.M., Mostafa O., Gazzar W.B.E., Sorour S.M., Seleem Y. (2018). Mesenchymal stem cell-derived exosomes ameliorated diabetic nephropathy by autophagy induction through the mTOR signaling pathway. Cells.

[644] Jiang Z.Z., Liu Y.M., Niu X., Yin J.Y., Hu B., Guo S.C., Fan Y., Wang Y., Wang N.S. (2016). Exosomes secreted by human urine-derived stem cells could prevent kidney complications from type I diabetes in rats. Stem Cell Res. Ther..

[645] Li F.X., Lin X., Xu F., Shan S.K., Guo B., Lei L.M., Zheng M.H., Wang Y., Xu Q.S., Yuan L.Q. (2021). The role of mesenchymal stromal cells-derived small extracellular vesicles in diabetes and its chronic complications. Front. Endocrinol..

[646] Djouad F., Jackson W.M., Bobick B.E., Janjanin S., Song Y., Huang G.T., Tuan R.S. (2010). Activin A expression regulates multipotency of mesenchymal progenitor cells. Stem Cell Res. Ther..

[647] Park S.E., Lee J., Chang E.H., Kim J.H., Sung J.H., Na D.L., Chang J.W. (2016). Activin A secreted by human mesenchymal stem cells induces neuronal development and neurite outgrowth in an in vitro model of Alzheimer’s disease: Neurogenesis induced by MSCs via activin A. Arch Pharm. Res..

[648] Szabat M., Johnson J.D., Piret J.M. (2010). Reciprocal modulation of adult β cell maturity by activin A and follistatin. Diabetologia.

[649] Setiawan A.M., Kamarudin T.A., Abd Ghafar N. (2022). The role of BMP4 in adipose-derived stem cell differentiation: A minireview. Front. Cell Dev. Biol..

[650] Wszoła M., Nitarska D., Cywoniuk P., Gomółka M., Klak M. (2021). Stem cells as a source of pancreatic cells for production of 3D bioprinted bionic pancreas in the treatment of type 1 diabetes. Cells.

[651] Zhang D., Jiang W., Liu M., Sui X., Yin X., Chen S., Shi Y., Deng H. (2009). Highly efficient differentiation of human ES cells and iPS cells into mature pancreatic insulin-producing cells. Cell Res.

[652] Sasaki M., Abe R., Fujita Y., Ando S., Inokuma D., Shimizu H. (2008). Mesenchymal stem cells are recruited into wounded skin and contribute to wound repair by transdifferentiation into multiple skin cell type. J. Immunol..

[653] Fiedler J., Röderer G., Günther K.P., Brenner R.E. (2002). BMP-2, BMP-4, and PDGF-bb stimulate chemotactic migration of primary human mesenchymal progenitor cells. J. Cell Biochem..

[654] Ren G., Zhao X., Wang Y., Zhang X., Chen X., Xu C., Yuan Z.R., Roberts A.I., Zhang L., Zheng B. (2012). CCR2-dependent recruitment of macrophages by tumor-educated mesenchymal stromal cells promotes tumor development and is mimicked by TNFα. Cell Stem Cell.

[655] Papa S., Vismara I., Mariani A., Barilani M., Rimondo S., De Paola M., Panini N., Erba E., Mauri E., Rossi F. (2018). Mesenchymal stem cells encapsulated into biomimetic hydrogel scaffold gradually release CCL2 chemokine in situ preserving cytoarchitecture and promoting functional recovery in spinal cord injury. J. Control Release.

[656] Kwon M.Y., Ghanta S., Ng J., Tsoyi K., Lederer J.A., Bronson R.T., El-Chemaly S., Chung S.W., Liu X., Perrella M.A. (2020). Expression of stromal cell-derived factor-1 by mesenchymal stromal cells impacts neutrophil function during sepsis. Crit. Care Med..

[657] Hocking A.M. (2015). The role of chemokines in mesenchymal stem cell homing to wounds. Adv. Wound Care.

[658] Mirabdollahi M., Haghjooy Javanmard S., Sadeghi-Aliabadi H. (2019). In vitro assessment of cytokine expression profile of MCF-7 cells in response to hWJ-MSCs secretome. Adv. Pharm. Bull..

[659] Kim D.H., Lee D., Chang E.H., Kim J.H., Hwang J.W., Kim J.Y., Kyung J.W., Kim S.H., Oh J.S., Shim S.M. (2015). GDF-15 secreted from human umbilical cord blood mesenchymal stem cells delivered through the cerebrospinal fluid promotes hippocampal neurogenesis and synaptic activity in an Alzheimer’s disease model. Stem Cells Dev..

[660] Kim D.H., Lee D., Lim H., Choi S.J., Oh W., Yang Y.S., Chang J.H., Jeon H.B. (2018). Effect of growth differentiation factor-15 secreted by human umbilical cord blood-derived mesenchymal stem cells on amyloid β levels in in vitro and in vivo models of Alzheimer’s disease. Biochem. Biophys. Res. Commun..

[661] Kim D.H., Lim H., Lee D., Choi S.J., Oh W., Yang Y.S., Oh J.S., Hwang H.H., Jeon H.B. (2018). Thrombospondin-1 secreted by human umbilical cord blood-derived mesenchymal stem cells rescues neurons from synaptic dysfunction in Alzheimer’s disease model. Sci. Rep..

[662] Hedbacker K., Birsoy K., Wysocki R.W., Asilmaz E., Ahima R.S., Farooqi I.S., Friedman J.M. (2010). Antidiabetic effects of IGFBP2, a leptin-regulated gene. Cell Metab..

[663] Lu J., Liu K.C., Schulz N., Karampelias C., Charbord J., Hilding A., Rautio L., Bertolino P., Östenson C.G., Brismar K. (2016). IGFBP1 increases β-cell regeneration by promoting α- to β-cell transdifferentiation. EMBO J..

[664] Fu Y., Karbaat L., Wu L., Leijten J., Both S.K., Karperien M. (2017). Trophic effects of mesenchymal stem cells in tissue regeneration. Tissue Eng. Part B Rev..

[665] Doni A., Stravalaci M., Inforzato A., Magrini E., Mantovani A., Garlanda C., Bottazzi B. (2019). The long Pentraxin PTX3 as a link between innate immunity, tissue remodeling, and cancer. Front. Immunol..

[666] Qi Y., Jiang D., Sindrilaru A., Stegemann A., Schatz S., Treiber N., Rojewski M., Schrezenmeier H., Vander Beken S., Wlaschek M. (2014). TSG-6 released from intradermally injected mesenchymal stem cells accelerates wound healing and reduces tissue fibrosis in murine full-thickness skin wounds. J. Investig. Dermatol..

[667] Occleston N.L., Laverty H.G., O’Kane S., Ferguson M.W. (2008). Prevention and reduction of scarring in the skin by Transforming Growth Factor beta 3 (TGFbeta3): From laboratory discovery to clinical pharmaceutical. J. Biomater. Sci. Polym. Ed..

[668] Martin K.E., Hunckler M.D., Chee E., Caplin J.D., Barber G.F., Kalelkar P.P., Schneider R.S., García A.J. (2023). Hydrolytic hydrogels tune mesenchymal stem cell persistence and immunomodulation for enhanced diabetic cutaneous wound healing. Biomaterials.

[669] Jiang D., Qi Y., Walker N.G., Sindrilaru A., Hainzl A., Wlaschek M., MacNeil S., Scharffetter-Kochanek K. (2013). The effect of adipose tissue derived MSCs delivered by a chemically defined carrier on full-thickness cutaneous wound healing. Biomaterials.

[670] Mazini L., Rochette L., Admou B., Amal S., Malka G. (2020). Hopes and limits of adipose-derived stem cells (ADSCs) and mesenchymal stem Cells (MSCs) in wound healing. Int. J. Mol. Sci..

[671] Oskowitz A., McFerrin H., Gutschow M., Carter M.L., Pochampally R. (2011). Serum-deprived human multipotent mesenchymal stromal cells (MSCs) are highly angiogenic. Stem Cell Res.

[672] Hung S.C., Pochampally R.R., Chen S.C., Hsu S.C., Prockop D.J. (2007). Angiogenic effects of human multipotent stromal cell conditioned medium activate the PI3K-Akt pathway in hypoxic endothelial cells to inhibit apoptosis, increase survival, and stimulate angiogenesis. Stem Cells.

[673] Prieto C.P., Ortiz M.C., Villanueva A., Villarroel C., Edwards S.S., Elliott M., Lattus J., Aedo S., Meza D., Lois P. (2017). Netrin-1 acts as a non-canonical angiogenic factor produced by human Wharton’s jelly mesenchymal stem cells (WJ-MSC). Stem Cell Res. Ther..

[674] Watt S.M., Gullo F., van der Garde M., Markeson D., Camicia R., Khoo C.P., Zwaginga J.J. (2013). The angiogenic properties of mesenchymal stem/stromal cells and their therapeutic potential. Br. Med. Bull..

[675] Németh K., Leelahavanichkul A., Yuen P.S., Mayer B., Parmelee A., Doi K., Robey P.G., Leelahavanichkul K., Koller B.H., Brown J.M. (2009). Bone marrow stromal cells attenuate sepsis via prostaglandin E_2_-dependent reprogramming of host macrophages to increase their interleukin-10 production. Nat. Med..

[676] Yang N., Baban B., Isales C.M., Shi X.M. (2015). Crosstalk between bone marrow-derived mesenchymal stem cells and regulatory T cells through a glucocorticoid-induced leucine zipper/developmental endothelial locus-1-dependent mechanism. FASEB J..

[677] Luz-Crawford P., Espinosa-Carrasco G., Ipseiz N., Contreras R., Tejedor G., Medina D.A., Vega-Letter A.M., Ngo D., Morand E.F., Pène J. (2018). Gilz-Activin A as a novel signaling axis orchestrating mesenchymal stem cell and Th17 cell interplay. Theranostics.

[678] Krampera M., Cosmi L., Angeli R., Pasini A., Liotta F., Andreini A., Santarlasci V., Mazzinghi B., Pizzolo G., Vinante F. (2006). Role for interferon-γ in the immunomodulatory activity of human bone marrow mesenchymal stem cells. Stem Cells.

[679] Wu H., Gong J., Liu Y. (2018). Indoleamine 2, 3-dioxygenase regulation of immune response (Review). Mol. Med. Rep..

[680] Hegyi B., Kudlik G., Monostori E., Uher F. (2012). Activated T-cells and pro-inflammatory cytokines differentially regulate prostaglandin E2 secretion by mesenchymal stem cells. Biochem. Biophys. Res. Commun..

[681] Yañez R., Oviedo A., Aldea M., Bueren J.A., Lamana M.L. (2010). Prostaglandin E2 plays a key role in the immunosuppressive properties of adipose and bone marrow tissue-derived mesenchymal stromal cells. Exp. Cell Res..

[682] Bouffi C., Bony C., Courties G., Jorgensen C., Noël D. (2010). IL-6-dependent PGE2 secretion by mesenchymal stem cells inhibits local inflammation in experimental arthritis. PLoS ONE.

[683] Eggenhofer E., Luk F., Dahlke M.H., Hoogduijn M.J. (2014). The life and fate of mesenchymal stem cells. Front. Immunol..

[684] Danchuk S., Ylostalo J.H., Hossain F., Sorge R., Ramsey A., Bonvillain R.W., Lasky J.A., Bunnell B.A., Welsh D.A., Prockop D.J. (2011). Human multipotent stromal cells attenuate lipopolysaccharide-induced acute lung injury in mice via secretion of tumor necrosis factor-α-induced protein 6. Stem Cell Res. Ther..

[685] de Araújo Farias V., Carrillo-Gálvez A.B., Martín F., Anderson P. (2018). TGF-β and mesenchymal stromal cells in regenerative medicine, autoimmunity and cancer. Cytokine Growth Factor Rev.

[686] Liu F., Qiu H., Xue M., Zhang S., Zhang X., Xu J., Chen J., Yang Y., Xie J. (2019). MSC-secreted TGF-β regulates lipopolysaccharide-stimulated macrophage M2-like polarization via the Akt/FoxO1 pathway. Stem Cell Res. Ther..

[687] He Z., Hua J., Qian D., Gong J., Lin S., Xu C., Wei G., Meng H., Yang T., Zhou B. (2016). Intravenous hMSCs ameliorate acute pancreatitis in mice via secretion of tumor necrosis factor-α stimulated gene/protein 6. Sci. Rep..

[688] Ren G., Su J., Zhang L., Zhao X., Ling W., L’Huillie A., Zhang J., Lu Y., Roberts A.I., Ji W. (2009). Species variation in the mechanisms of mesenchymal stem cell-mediated immunosuppression. Stem Cells.

[689] Najar M., Rouas R., Raicevic G., Boufker H.I., Lewalle P., Meuleman N., Bron D., Toungouz M., Martiat P., Lagneaux L. (2009). Mesenchymal stromal cells promote or suppress the proliferation of T lymphocytes from cord blood and peripheral blood: The importance of low cell ratio and role of interleukin-6. Cytotherapy.

[690] Qu X., Liu X., Cheng K., Yang R., Zhao R.C. (2012). Mesenchymal stem cells inhibit Th17 cell differentiation by IL-10 secretion. Exp. Hematol..

[691] English K., Barry F.P., Field-Corbett C.P., Mahon B.P. (2007). IFN-γ and TNF-α differentially regulate immunomodulation by murine mesenchymal stem cells. Immunol. Lett..

[692] Rizzo R., Lanzoni G., Stignani M., Campioni D., Alviano F., Ricci F., Tazzari P.L., Melchiorri L., Scalinci S.Z., Cuneo A. (2011). A simple method for identifying bone marrow mesenchymal stromal cells with a high immunosuppressive potential. Cytotherapy.

[693] Kyurkchiev D., Bochev I., Ivanova-Todorova E., Mourdjeva M., Oreshkova T., Belemezova K., Kyurkchiev S. (2014). Secretion of immunoregulatory cytokines by mesenchymal stem cells. World J. Stem Cells.

[694] Bartosh T.J., Ylostalo J.H. (2019). Efficacy of 3D culture priming is maintained in human mesenchymal stem cells after extensive expansion of the cells. Cells.

[695] Ylostalo J.H., Bartosh T.J., Tiblow A., Prockop D.J. (2014). Unique characteristics of human mesenchymal stromal/progenitor cells pre-activated in 3-dimensional cultures under different conditions. Cytotherapy.

[696] Ylöstalo J.H., Bartosh T.J., Coble K., Prockop D.J. (2012). Human mesenchymal stem/stromal cells cultured as spheroids are self-activated to produce prostaglandin E2 that directs stimulated macrophages into an anti-inflammatory phenotype. Stem Cells.

[697] Choi H., Lee R.H., Bazhanov N., Oh J.Y., Prockop D.J. (2011). Anti-inflammatory protein TSG-6 secreted by activated MSCs attenuates zymosan-induced mouse peritonitis by decreasing TLR2/NF-κB signaling in resident macrophages. Blood.

[698] Lee R.H., Pulin A.A., Seo M.J., Kota D.J., Ylostalo J., Larson B.L., Semprun-Prieto L., Delafontaine P., Prockop D.J. (2009). Intravenous hMSCs improve myocardial infarction in mice because cells embolized in lung are activated to secrete the anti-inflammatory protein TSG-6. Cell Stem Cell.

[699] Oh J.Y., Ko J.H., Lee H.J., Yu J.M., Choi H., Kim M.K., Wee W.R., Prockop D.J. (2014). Mesenchymal stem/stromal cells inhibit the NLRP3 inflammasome by decreasing mitochondrial reactive oxygen species. Stem Cells.

[700] Zhang R., Liu Q., Zhou S., He H., Zhao M., Ma W. (2023). Mesenchymal stem cell suppresses the efficacy of CAR-T toward killing lymphoma cells by modulating the microenvironment through stanniocalcin-1. eLife.

[701] Block G.J., Ohkouchi S., Fung F., Frenkel J., Gregory C., Pochampally R., DiMattia G., Sullivan D.E., Prockop D.J. (2009). Multipotent stromal cells are activated to reduce apoptosis in part by upregulation and secretion of stanniocalcin-1. Stem Cells.

[702] Kulesza A., Paczek L., Burdzinska A. (2023). The role of COX-2 and PGE2 in the regulation of immunomodulation and other functions of mesenchymal stromal cells. Biomedicines.

[703] Chatterjee D., Marquardt N., Tufa D.M., Hatlapatka T., Hass R., Kasper C., von Kaisenberg C., Schmidt R.E., Jacobs R. (2014). Human umbilical cord-derived mesenchymal stem cells utilize activin-A to suppress interferon-γ production by natural killer cells. Front. Immunol..

[704] Ren G., Zhang L., Zhao X., Xu G., Zhang Y., Roberts A.I., Zhao R.C., Shi Y. (2008). Mesenchymal stem cell-mediated immunosuppression occurs via concerted action of chemokines and nitric oxide. Cell Stem Cell.

[705] Huber S., Stahl F.R., Schrader J., Lüth S., Presser K., Carambia A., Flavell R.A., Werner S., Blessing M., Herkel J. (2009). Activin A promotes the TGF-β-induced conversion of CD4^+^CD25^-^ T cells into Foxp3^+^ induced regulatory T cells. J. Immunol..

[706] Rovere P., Peri G., Fazzini F., Bottazzi B., Doni A., Bondanza A., Zimmermann V.S., Garlanda C., Fascio U., Sabbadini M.G. (2000). The long pentraxin PTX3 binds to apoptotic cells and regulates their clearance by antigen-presenting dendritic cells. Blood.

[707] Bourhis M., Palle J., Galy-Fauroux I., Terme M. (2021). Direct and indirect modulation of T Cells by VEGF-A counteracted by anti-angiogenic treatment. Front. Immunol..

[708] Hu X., Wu R., Shehadeh L.A., Zhou Q., Jiang C., Huang X., Zhang L., Gao F., Liu X., Yu H. (2014). Severe hypoxia exerts parallel and cell-specific regulation of gene expression and alternative splicing in human mesenchymal stem cells. BMC Genom..

[709] Raugh A., Jing Y., Bettini M.L., Bettini M. (2023). The Amphiregulin/EGFR axis has limited contribution in controlling autoimmune diabetes. Res. Sq. Prepr..

[710] Ibáñez L., Nácher-Juan J., Terencio M.C., Ferrándiz M.L., Alcaraz M.J. (2022). Osteostatin inhibits M-CSF+RANKL-induced human osteoclast differentiation by modulating NFATc1. Int. J. Mol. Sci..

[711] Su Z., He L., Shang H., Dai T., Xu F., Zhao J. (2020). Overexpression of bone morphogenetic protein-1 promotes osteogenesis of bone marrow mesenchymal stem cells in vitro. Med. Sci. Monit..

[712] Hopkins D.R., Keles S., Greenspan D.S. (2007). The bone morphogenetic protein 1/Tolloid-like metalloproteinases. Matrix Biol..

[713] Kim B., Huang G., Ho W.B., Greenspan D.S. (2011). Bone morphogenetic protein-1 processes insulin-like growth factor-binding protein 3. J. Biol. Chem..

[714] Jiang H., Hong T., Wang T., Wang X., Cao L., Xu X., Zheng M. (2019). Gene expression profiling of human bone marrow mesenchymal stem cells during osteogenic differentiation. J. Cell Physiol..

[715] Pepin É., Al-Mass A., Attané C., Zhang K., Lamontagne J., Lussier R., Madiraju S.R., Joly E., Ruderman N.B., Sladek R. (2016). Pancreatic β-cell dysfunction in diet-induced obese mice: Roles of AMP-kinase, protein kinase Cε, mitochondrial and cholesterol metabolism, and alterations in gene expression. PLoS ONE.

[716] Hoxha E., Marcinnò A., Montarolo F., Masante L., Balbo I., Ravera F., Laezza F., Tempia F. (2019). Emerging roles of Fgf14 in behavioral control. Behav. Brain Res..

[717] Johansson U., Olsson A., Gabrielsson S., Nilsson B., Korsgren O. (2003). Inflammatory mediators expressed in human islets of Langerhans: Implications for islet transplantation. Biochem. Biophys. Res. Commun..

[718] Clarkin C.E., Mahmoud M., Liu B., Sobamowo E.O., King A., Arthur H., Jones P.M., Wheeler-Jones C.P. (2016). Modulation of endoglin expression in islets of langerhans by VEGF reveals a novel regulator of islet endothelial cell function. BMC Res. Notes.

[719] Christofori G., Naik P., Hanahan D. (1995). Vascular endothelial growth factor and its receptors, flt-1 and flk-1, are expressed in normal pancreatic islets and throughout islet cell tumorigenesis. Mol. Endocrinol..

[720] Zhou Y., Zeng J., Tu Y., Li L., Du S., Zhu L., Cang X., Lu J., Zhu M., Liu X. (2021). CSF1/CSF1R-mediated crosstalk between choroidal vascular endothelial cells and macrophages promotes choroidal neovascularization. Investig. Ophthalmol. Vis. Sci..

[721] Corliss B.A., Azimi M.S., Munson J.M., Peirce S.M., Murfee W.L. (2016). Macrophages: An Inflammatory Link Between Angiogenesis and Lymphangiogenesis. Microcirculation.

[722] Shibata H., Yasuda H., Sekine N., Mine T., Totsuka Y., Kojima I. (1993). Activin A increases intracellular free calcium concentrations in rat pancreatic islets. FEBS Lett..

[723] Wada M., Shintani Y., Kosaka M., Sano T., Hizawa K., Saito S. (1996). Immunohistochemical localization of activin A and follistatin in human tissues. Endocr. J..

[724] Eberhard D. (2013). Neuron and β-cell evolution: Learning about neurons is learning about β-cells. Bioessays.

[725] Ross-Munro E., Kwa F., Kreiner J., Khore M., Miller S.L., Tolcos M., Fleiss B., Walker D.W. (2020). Midkine: The who, what, where, and when of a promising neurotrophic therapy for perinatal brain injury. Front. Neurol..

[726] Sevillano J., Liang A., Strutt B., Hill T.G., Szlapinski S., Ramos-Álvarez M.P., Hill D.J. (2022). Pleiotrophin expression and actions in pancreatic β-cells. Front. Endocrinol..

[727] Li Z., Mehta S.S., Prasadan K., Hembree M., Holcomb G.W., Ostlie D.J., Snyder C.L., Gittes G.K. (2003). Pleiotrophin signaling in pancreatic organogenesis and differentiation. J. Surg. Res..

[728] Sevillano J., Sánchez-Alonso M.G., Zapatería B., Calderón M., Alcalá M., Limones M., Pita J., Gramage E., Vicente-Rodríguez M., Horrillo D. (2019). Pleiotrophin deletion alters glucose homeostasis, energy metabolism and brown fat thermogenic function in mice. Diabetologia.

[729] Ballesteros-Pla C., Sánchez-Alonso M.G., Pizarro-Delgado J., Zuccaro A., Sevillano J., Ramos-Álvarez M.P. (2023). Pleiotrophin and metabolic disorders: Insights into its role in metabolism. Front. Endocrinol..

[730] Verhoeff K., Cuesta-Gomez N., Jasra I., Marfil-Garza B., Dadheech N., Shapiro A.M.J. (2022). Optimizing generation of stem cell-derived islet cells. Stem Cell Rev. Rep..

[731] Wang X., Gao M., Wang Y., Zhang Y. (2022). The progress of pluripotent stem cell-derived pancreatic β-cells regeneration for diabetic therapy. Front. Endocrinol..

[732] Rezania A., Bruin J.E., Arora P., Rubin A., Batushansky I., Asadi A., O’Dwyer S., Quiskamp N., Mojibian M., Albrecht T. (2014). Reversal of diabetes with insulin-producing cells derived in vitro from human pluripotent stem cells. Nat. Biotechnol..

[733] Yabe S.G., Fukuda S., Takeda F., Nashiro K., Shimoda M., Okochi H. (2017). Efficient generation of functional pancreatic β-cells from human induced pluripotent stem cells. J. Diabetes.

[734] Sui L., Leibel R.L., Egli D. (2018). Pancreatic β cell differentiation from human pluripotent stem cells. Curr. Protoc. Hum. Genet..

[735] Nair G.G., Liu J.S., Russ H.A., Tran S., Saxton M.S., Chen R., Juang C., Li M.L., Nguyen V.Q., Giacometti S. (2019). Recapitulating endocrine cell clustering in culture promotes maturation of human stem-cell-derived β cells. Nat. Cell Biol..

[736] Velazco-Cruz L., Song J., Maxwell K.G., Goedegebuure M.M., Augsornworawat P., Hogrebe N.J., Millman J.R. (2019). Acquisition of dynamic function in human stem cell-derived β cells. Stem Cell Rep..

[737] Mfopou J.K., Chen B., Mateizel I., Sermon K., Bouwens L. (2010). Noggin, retinoids, and fibroblast growth factor regulate hepatic or pancreatic fate of human embryonic stem cells. Gastroenterology.

[738] Kroon E., Martinson L.A., Kadoya K., Bang A.G., Kelly O.G., Eliazer S., Young H., Richardson M., Smart N.G., Cunningham J. (2008). Pancreatic endoderm derived from human embryonic stem cells generates glucose-responsive insulin-secreting cells in vivo. Nat. Biotechnol..

[739] Kunisada Y., Tsubooka-Yamazoe N., Shoji M., Hosoya M. (2012). Small molecules induce efficient differentiation into insulin-producing cells from human induced pluripotent stem cells. Stem Cell Res..

[740] Jin W., Jiang W. (2022). Stepwise differentiation of functional pancreatic β cells from human pluripotent stem cells. Cell Regen..

[741] Russ H.A., Parent A.V., Ringler J.J., Hennings T.G., Nair G.G., Shveygert M., Guo T., Puri S., Haataja L., Cirulli V. (2015). Controlled induction of human pancreatic progenitors produces functional β-like cells in vitro. EMBO J..

[742] Assmann A., Hinault C., Kulkarni R.N. (2009). Growth factor control of pancreatic islet regeneration and function. Pediatr. Diabetes.

[743] Oliver-Krasinski J.M., Stoffers D.A. (2008). On the origin of the beta cell. Genes Dev..

