# Supplementary material for: A Supportive Role of Mesenchymal Stem Cells on Insulin-Producing Langerhans Islets with a Specific Emphasis on The Secretome
Source: Biomedicines. 2023 Sep 18;11(9):2558. doi: 10.3390/biomedicines11092558 (PMC10527322; doi:10.3390/biomedicines11092558)
Supplement: Supplementary file 1 [file biomedicines-11-02558-s001.zip › biomedicines-2587247-supplementary material-updated.pdf]

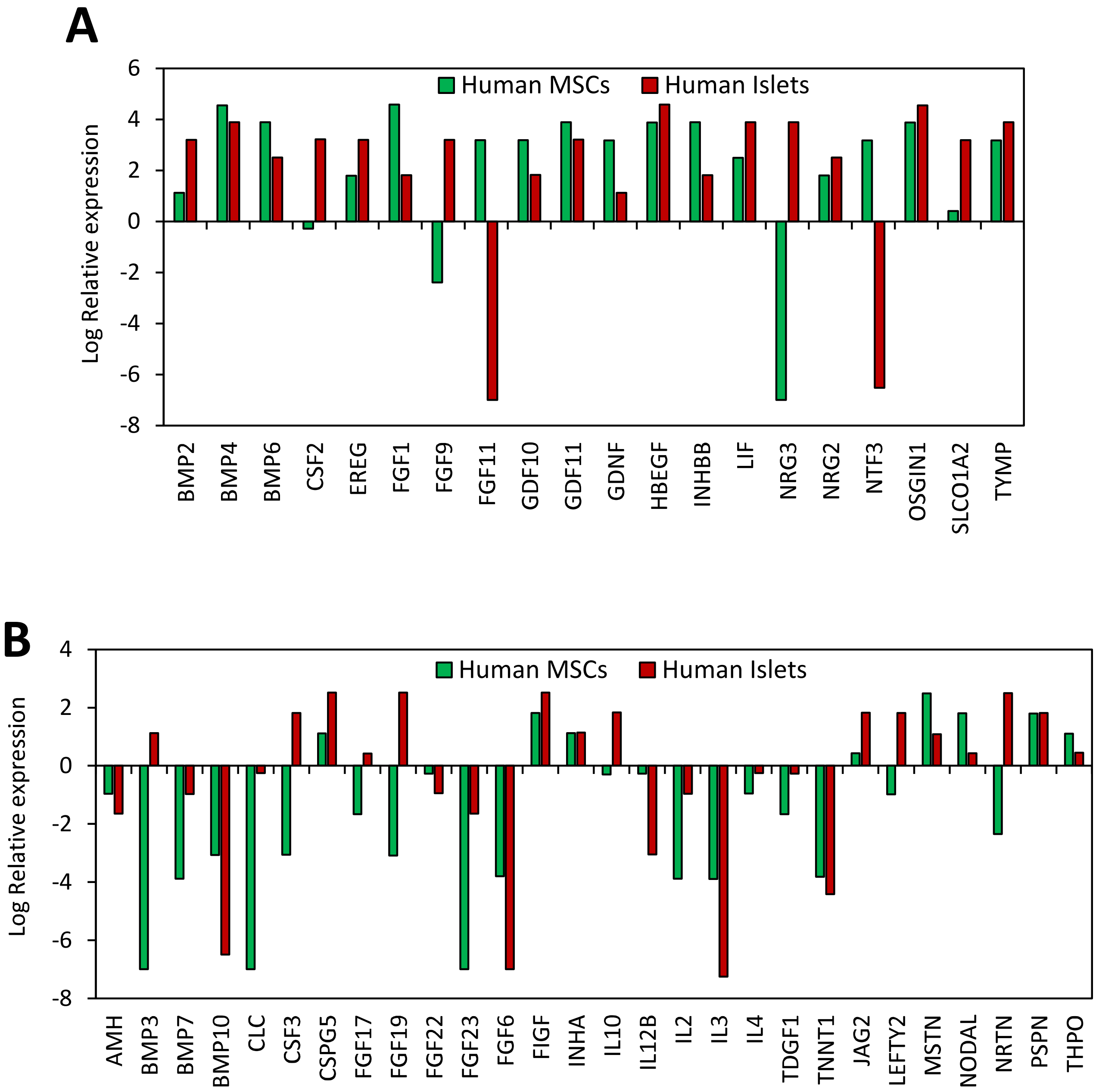

Supplementary Figure S1. A-B. Genes expressed at low and very low levels in human MSCs and human islets according to the human growth factor RT Profiler PCR Array.

Supplementary Material and Methods

Cell culture

Human islets were obtained from PRODO Laboratories Inc. (Irvine, California, USA) and were incubated overnight in CMRL-1066 (Biological Industries, Beth HaEmek, Israel) supplemented with 10% heat-inactivated fetal calf serum (FCS) (Biological Industries, Beth HaEmek, Israel), 2 mM L-glutamine, 1 mM sodium pyruvate, non-essential amino acids, and 10 U/mL penicillin, 100 μg/mL streptomycin (Biological Industries, Beth HaEmek, Israel), and 50 μg/mL gentamicin (Sigma, St. Louis, USA). Human bone marrow-derived mesenchymal stem cells (MSCs) were obtained from Lonza (Catalog number PT-2501, Walkersville, MD, USA) and cultivated in low glucose DMEM (Biological Industries, Beth HaEmek, Israel) supplemented with 10% heat-inactivated FCS, 2 mM L-glutamine, 1 mM sodium pyruvate, non-essential amino acids and penicillin-streptomycin. All cell cultures were incubated at 37 °C in a humidified incubator supplemented with 5% CO_2.

DNA Microarrays

The human growth factor RT Profiler PCR Array (PAHS-041A, Qiagen, Maryland, USA) was used according to the manufacturer's instructions to profiling 84 genes related to growth factors and cytokines in human MSCs and human islets.
